# Metal-Free Graphene-Based Derivatives as Oxygen Reduction Reaction Electrocatalysts in Energy Conversion and Storage Systems: An Overview

**DOI:** 10.3390/molecules30102248

**Published:** 2025-05-21

**Authors:** Laura Crociani

**Affiliations:** Institute of Condensed Matter Chemistry and Technologies for Energy, ICMATE, National Research Council of Italy, CNR, Corso Stati Uniti, 4, 35127 Padua, Italy; laura.crociani@cnr.it

**Keywords:** graphene, oxygen reduction reaction, electrocatalysis, fuel cells, metal–air batteries

## Abstract

Oxygen reduction reaction (ORR) is one of the most important reactions in electrochemical energy storage and conversion devices. To overcome the slow kinetics, minimize the overpotential, and make this reaction feasible, efficient, and stable, electrocatalysts are needed. Metal-free graphene-based systems are considered promising and cost-effective ORR catalysts with adjustable structures. This review is meant to give a rational overview of the graphene-based metal-free ORR electrocatalysts, illustrating the huge amount of related research developed particularly in the field of fuel cells and metal–air batteries, with particular attention to the synthesis procedures. The novelty of this review is that, beyond general aspects regarding the synthesis and characterization of graphene, above 90% of the various graphene (doped and undoped species, composites)-based ORR electrocatalysts have been reported, which represents an unprecedented thorough collection of both experimental and theoretical studies. Hundreds of references are included in the review; therefore, it can be considered as a *vademecum* in the field.

## 1. Introduction

The oxygen reduction reaction (ORR) is one of the processes at the base of the functioning of energy conversion and storage systems such as fuel cells and metal–air batteries, and the key energy conversion reaction involved in the production of hydrogen peroxide.

The electrochemical reduction of molecular oxygen can proceed through two or four-electron transfers in aqueous electrolyte. In alkaline and neutral media, the reaction mechanism can be described as follows [1]:
Four-electron pathway: O_2_ + 2H_2_O + 4e- → 4OH- E = 0.401 VTwo-electron pathway: O_2_ + H_2_O + 2e- → HO_2_-+ OH-E = −0.065 V
HO_2_- + H_2_O + 2e- → 3OH- E = 0.867 V
2HO_2_- → 2OH- + O_2_


In acidic electrolyte, the final products are H_2_O and H_2_O_2_ for the four and the two-electron pathway, respectively.
Four-electron pathway: O_2_ + 4H^+^ + 4e- → 2H_2_O E = +1.229 VTwo-electron pathway: O_2_ + 2H^+^ + 2e- → H_2_O_2_
E = +0.67 V
H_2_O_2_ + 2H^+^ + 2e- → 2H_2_O E = +1.77 V

This reaction occurring at the cathode, however, presents a high activation energy related to the strong bond energy (498 kJmol^−1^) of the O=O bond, so that the improvement of kinetics and reduction in overpotentials require the utilization of electrocatalysts lowering the energy barrier for the bond activation and cleavage.

The electrochemical assessment of the ORR electrocatalyst performance occurs through the determination of key kinetic parameters of ORR, such as onset potential, half-wave potential, kinetic current density, mass and specific activity, Tafel slope, and conductivity using conventional voltammetry techniques, while the Rotating Disk Electrode (RDE) and rotating ring-disk electrode (RRDE) techniques under hydrodynamic conditions analyze the influence of kinetics and the mass-transfer effect on ORR [2]. Additionally, the number of electrons transferred (n) can be preliminarily obtained by the Koutecky–Levich equation shown below:

$$\frac{1}{I}=-\frac{1}{I_{K}}+\frac{1}{0.62\mathrm{FnAB}v^{1/6}D^{2/3}\omega^{1/2}{C_{O}}_{2}}$$

where I is the reaction current; I_k_ is the kinetic current; n is the number of exchanged electrons per O_2_ molecule; F is Faraday constant; A is the electrode area; D is the diffusion coefficient of O_2_ in the electrolyte; v is the kinematic viscosity of the electrolyte; C_O2_ is the bulk concentration of O_2_; and ω is the rotation rate of the electrode [3].

To date, the most commonly used ORR electrocatalysts are platinum-based catalysts consisting of Pt or Pt alloy nanoparticles supported on porous carbon species. However, despite their high activity, platinum-based catalysts present some drawbacks related to the high cost of platinum and limited stability under operating conditions.

Therefore, research has moved to other noble and non-noble metal-based catalysts as well as metal-free species, particularly metal-free carbon materials.

Among the various forms of carbon materials, graphene, a monolayer of graphite containing a honeycomb structure of carbon atoms, represents an emerging material generating huge interest because of its enormous surface area (2630 m^2^g^−1^), excellent electrical conductivity (106 Scm^−1^), good thermal conductivity (∼5000 Wm^−1^K^−1^), high charge mobility (200,000 cm^2^V^−1^s^−1^), great mechanical strength (breaking strength of 42 Nm^−1^ and Young’s modulus of 1.0 TPa), low optical absorbance (2.3%) and density (<1 gcm^−3^), and unusual flexibility [4].

For electrochemical applications, graphene-based electrodes are expected, theoretically, to react much faster, showing that graphene has faster electron mobility in comparison to all other potential materials [5].

Owing to the unique features of graphene, a great deal of research has been focused on its electrochemical applications as a promising material for electrodes, particularly in fuel cells [6], aqueous metal–air batteries [7], and lithium batteries [8,9,10].

Fuel cells, which produce energy upon the electrochemical oxidation of a fuel and reduction of oxygen, have been developed to respond to the increasing global energy demand facing the increasing scarcity of fossil fuels, and to meet the requirement for environmental sustainability [11].

**Figure 1:**
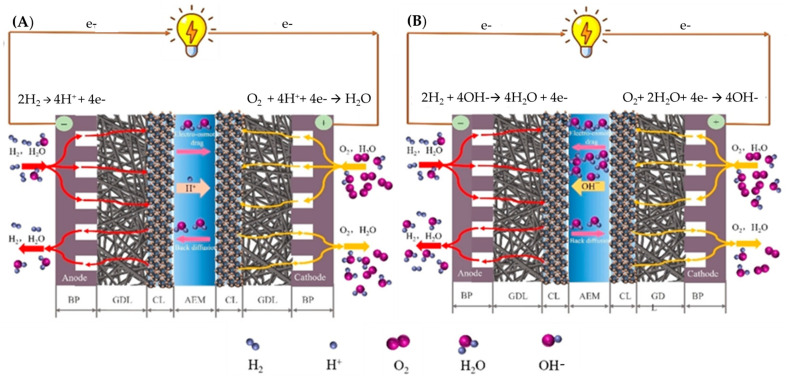
Schematic representation of (**A**) PEMFC and (**B**) AEMFC: the reactions occurring at both anode and cathode during their operation with H_2_ are reported [13]. © 2023 The Chemical Society of Japan and Wiley-VCH GmbH.

Among fuel cells, polymer electrolyte membrane fuel cells specifically operating at low temperature (below 120 °C) convert the energy of a fuel such as hydrogen or methanol into electricity with low or zero emissions and high efficiency. They can be classified in terms of the electrolyte in the cell [12] into Proton Exchange Membrane Fuel Cell (PEMFC) (Figure 1A) or Anionic Exchange Membrane Fuel Cell (AEMFC) (Figure 1B). In acidic electrolytes, proton transfer from the anode to the cathode is promoted, while in alkaline electrolytes, hydroxyl ion is transferred from the cathode to the anode, so that the type of components, such as the catalyst layers and the membrane, may differ depending on the electrolyte.

**Figure 2:**
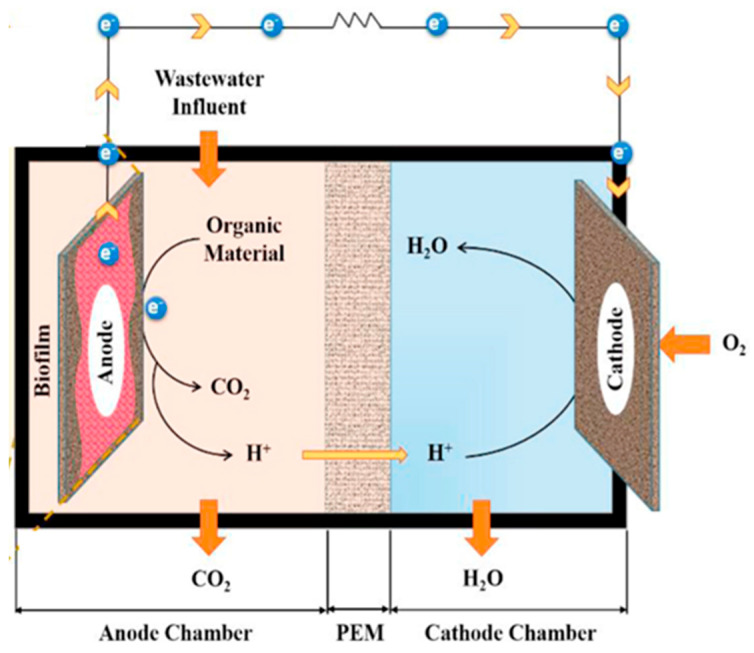
Microbial fuel cell scheme from [14]. © 2016 Elsevier Ltd. (2016).

A special type of polymer electrolyte membrane fuel cell, very attractive for its environmental applications, is the microbial fuel cell (MFC), which converts the chemical energy of organic matter such as carbohydrates and organic acids into electricity directly using microorganisms. The wastewater is used as fuel for producing electrical power (Figure 2) [14].

MFCs can, therefore, be applied for wastewater treatment with the double benefit of recovering energy from wastewater as well as reducing the biochemical oxygen demand and excess sludge production.

Besides fuel cells, which are important energy conversion systems, other power supply devices include the aqueous metal batteries, which constitute an emerging energy storage technology for renewable energy. In particular, multivalent ion batteries, such as zinc (Zn), calcium (Ca), aluminum (Al), and magnesium (Mg) ion batteries, are of particular interest, considering the natural abundance of Al, Ca, Mg, and Zn in the Earth’s crust, their low redox potentials, and relevant energy density due to the transfer of several electrons per metallic cation in the electrochemical reactions involving the metals [15].

**Figure 3:**
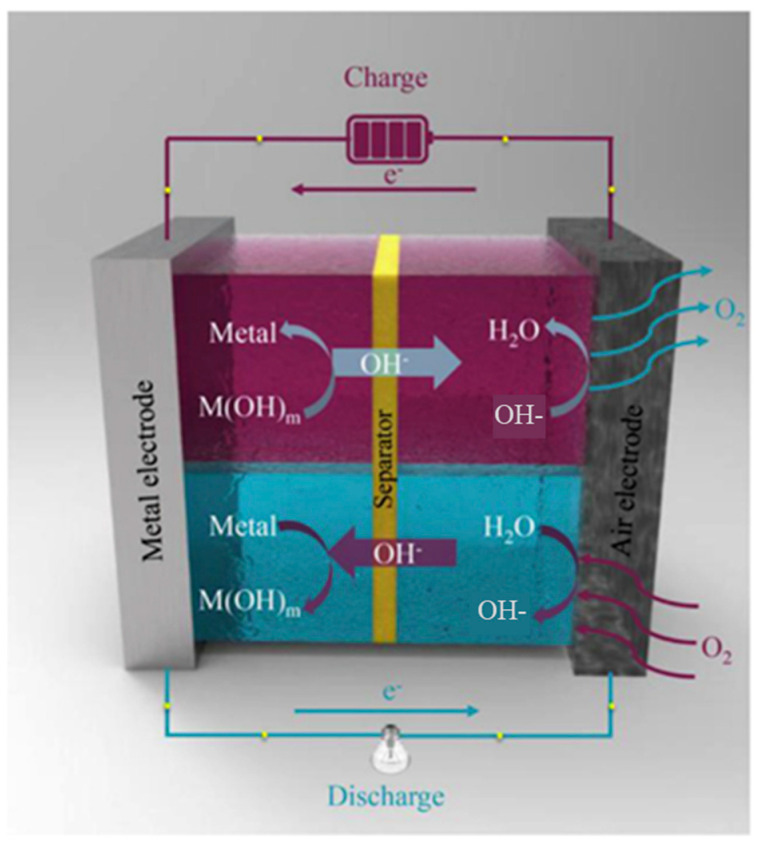
Schematic operating process of aqueous metal–air batteries from [16]. © 2019 Elsevier B.V. (2019).

The basic operating scheme is presented in Figure 3; during the discharge process, the metal releases electrons and metal hydroxide is formed; electrons are supplied to the cathode for the oxygen reduction.

The overall reaction occurring in the battery is as follows:

$$M+\frac{n}{4}O_{2}+\frac{n}{2}\;H_{2}O\to {\text{M(OH)}}_{n}$$

Hence, on the basis of the reported electrochemical applications where the oxygen reduction reaction is a fundamental process, the goal of this review is to present the development of metal-free graphene as ORR electrocatalysts, comprehending sections describing the synthetic methods, specific characterizations, and the doped and undoped graphene-based specifically as ORR electrocatalysts, ECs. Tables containing ORR parameters such as onset potential (E_onset_) and catalyst loading of the electrocatalysts in alkaline, acidic, and neutral electrolytes are also presented. A list of the used abbreviations is provided in the supporting information.

## 2. Graphene Synthesis Methods

Since the first synthesis of graphene via mechanical exfoliation (repeated peeling) of small mesas of highly oriented pyrolytic graphite (HOPG) [17], the huge interest in such a material has pushed research in finding preparation methods that can produce a low-cost graphene in good yields and in the most effective way in terms of composition, morphology, structure, and properties such as stacked layer, lateral size, defect and impurity contents, surface chemistry, and solubility, as well as electrical and thermal conductivities.

Several techniques have been used, depending on the synthetic strategy, which can be based on a “top-down” or a “bottom-up” approach.

The top-down technique proceeds as follows: (i) isolation of graphene from the stacked parent materials by solid-phase, liquid-phase, or electrochemical exfoliation of pristine graphite and graphite intercalated compounds, and (ii) reduction of graphene oxide (GO) obtained via exfoliation of graphite. The bottom-up approach involves building up graphene from molecular precursors, typically including arc discharge, chemical vapor deposition (CVD), epitaxial growth, on-surface synthesis, laser irradiation, and pyrolysis [18].

In general, top-down methods are more advantageous from the viewpoints of scalability and cost-effectiveness, whereas bottom-up processes are appropriate for producing large-area, high-quality films [19].

Some synthesis routes possessing different scalability and generating graphene with different characteristics are listed in Table 1, including their advantages and disadvantages [20].

**Table 1:** Summary of techniques for synthesizing graphene.

Approach	Technique and Brief Description	Advantage/Disadvantage
**Top-down**	Micromechanical exfoliation: exfoliation of layers from graphite by Scotch tape	Low output but simple process with high quality
	Chemical reduction of graphite derivative: exfoliation of graphite derivative in suspension and chemical reduction	Large-scale production with significant defects
**Bottom-up**	Chemical vapor deposition: decomposition of hydrocarbon on a metal substrate at high temperature	Large area graphene, but very poor yield
	On-surface synthesis: covalent fusion of molecular building blocks onto a metallic surface	Atomic precision in regularly ordered porous graphene sheets, nanographenes, and graphene nanoribbons, but poor scalability
	Laser irradiation: conversion of carbon-based precursors induced by laser	Fast, low-cost, and energy-saving process, but inhomogeneity and difficult control of morphology
	Pyrolysis: thermal conversion of carbon-based precursors	Large-area monolayer graphene films onto a variety of substrates. Limited yield, energy consumption
	Epitaxial growth: evaporation of SiC on Si-wafer at high temperature	Micron-length graphene with few defects, but poor scalability and costly method

### 2.1. Top-Down Graphene Synthesis

In the top-down approach, the final graphene/heteroatom-doped graphene is obtained starting from a precursor that already includes one-atom-thick layers, such as HOPG and graphene oxide, through both physical and chemical exfoliation processes, and, where necessary, chemical reduction.

Mechanical exfoliation of pristine graphite using Scotch tape produced high-quality graphene layers with good crystalline structure, low defect density, and high electrical conductivity, but at low yields [13].

**Figure 4:**
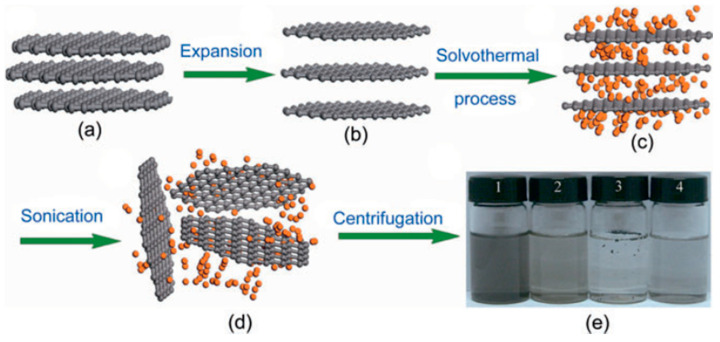
Schematic illustration of solvothermal-assisted exfoliation and dispersion of graphene sheets in acetonitrile: (**a**) pristine expandable graphite; (**b**) ExG obtained by heating at 1000 °C under an atmosphere of forming gas (5% H_2_ and 95% Ar); (**c**) insertion of acetonitrile molecules into the interlayers of ExG; (**d**) exfoliated graphene sheets dispersed in acetonitrile; (**e**) optical images of four samples obtained under different processes and centrifugation conditions [22].

Since large-scale exfoliation is needed, more effective techniques were developed, including liquid exfoliation based on the use of organic solvents like N-methyl-pyrrolidone (NMP) and dimethylformamide (DMF): in this way, the energy required to exfoliate graphene could be balanced by the solvent–graphene interaction for solvents whose surface energies match that of graphene [21]. It was also observed that further improvement of the yield was obtained if exfoliation of graphite in polar solvents is assisted by other processes, such as a solvothermal process. Qian et al. [22] readily produced monolayer and bilayer graphene by solvothermal-assisted exfoliation and dispersion in acetonitrile (Figure 4), followed by centrifugation in 10–12 wt% yield by using expanded graphite (ExG) as the starting material.

In fact, expanded graphite has the advantage of a larger interlayer spacing than pristine graphite, which weakens the van der Waals interactions and facilitates exfoliation [23]. However, because of several drawbacks in using polar organic solvents (e.g., toxicity, hazard, etc.), research has been advanced to develop water-based processes using surfactants [24] such as sodium dodecylbenzene sulfonate [25], sodium cholate [21], and alkaline lignin [26], or ionic liquids [27] such as 1-butyl-3-methylimidazolium peroxydisulfate [28] and 1-ethyl-3-methylimidazolium tetrafluoroborate [29], as well as butyltrimethylammonium bis(trifluoromethylsulfonyl)imide [30].

Ionic liquids have also been used in the fabrication of graphene nanosheets via electrochemical exfoliation of graphite [31,32]. This process is considered a potentially scalable method for synthesizing graphene in a relatively short time at ambient temperature, generating gram-scale quantities of graphene materials with tunable quality using operating voltages, graphite precursors, and electrolytes [33].

Natural graphite [31,34] and synthetic graphite [32,35] can be used in different forms (powder [36], foil [37], rod [38,39], flake [40], flexible sheet [41], or plat [42,43]) and the electrolytes can be a mixture of water and ionic liquids [44,45], aqueous solution of inorganic salts [46], alkaline electrolyte [47], or mineral acids [47]; under such conditions, graphene is obtained via anodic exfoliation of the precursors. On the other hand, cathodic exfoliation of graphite occurs in organic solvents, such as propylene carbonate [48], dimethyl sulfoxide [49], and N-methyl-2-pyrrolidone [50], containing lithium or alkylammonium salts.

An extension to liquid-phase exfoliation consists of using supercritical fluids (SCFs), which have both gas- and liquid-like physicochemical properties: at the supercritical state, i.e., when temperature and pressure are above the critical points, SCFs have near-zero surface tension, low viscosity, high solvating power, and diffusion coefficients, which are advantageous for penetrating graphite’s interlayer spaces and facilitating exfoliation. Compared to ordinary liquids, the density and solvent strength of the SCF can be tuned by changing pressure and/or temperature.

The choice of SCF is crucial as the interaction between solvent and graphite layers should be sufficient to offset the van der Waals attraction between graphitic layers, thereby making the graphene layers loose [51]; actually, intercalation and exfoliation under SCF conditions imply rapid penetration of solvent molecules through the interlayers of graphite, achieving delamination of graphitic materials.

SCFs comprehend supercritical organic solvents, like DMF, NMP, and alcohols [52], and dimethyl sulfoxide [53]. Tomai et al. [54] prepared nanographene with a diameter of less than 100 nm from platelet carbon nanofibers as an alternative to graphite in supercritical ethanol. More recently, water, whose use would be advantageous due to its low cost, minimal safety concerns, and relatively low environmental impact, has been utilized alone in supercritical conditions as reported by Ibarra et al. [55], who prepared large graphene layers with sizes of around 5 μm from graphite powder. Most graphene exfoliation using supercritical fluids has been carried out using CO_2_ since first synthesis by Pu et al. 2010 [56,57,58]. Supercritical CO_2_ is widely used as a nonflammable, nontoxic, environmentally friendly solvent with an easily accessible critical point (TC = 31.1 °C and PC = 73.8 b). However, the non-polar nature of CO_2_ and the rapid escape of CO_2_ molecules from the intercalated layers make exfoliation of graphite with ScCO_2_ alone less effective than exfoliation with organic solvents [51].

Therefore, various enhancements were applied to improve the quality and yield of exfoliated graphene using the scCO_2_ exfoliation process, including mixing CO_2_ with liquids [59,60] or using assistant methods such as ultrasonic treatment [61], rotor/stator mixing [62], and ball milling [63].

**Figure 5:**
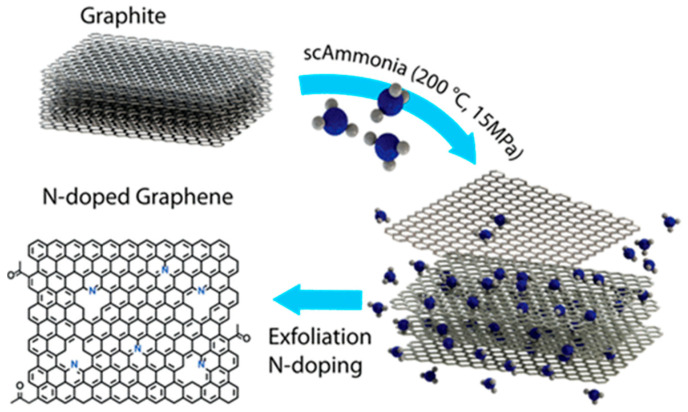
Exfoliation and N doping using supercritical ammonia. Reprinted with permission from [64]. Copyright {2016} American Chemical Society.

It is also interesting to report that exfoliation and N doping can be achieved simultaneously using supercritical ammonia (scNH_3_) [64] (Figure 5).

N-doped graphene consisting of a few layers (<5) with a sizeable lateral size was produced with a 40% yield.

**Figure 6:**
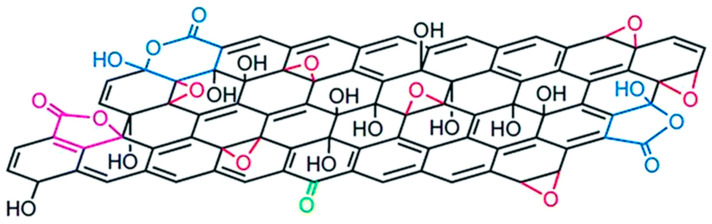
Structure of GO [66] reprinted with permission from Springer Nature Chemistry © 2009 Macmillan Publishers Limited.

In addition to physical methods, exfoliation of graphite can also be realized chemically to produce graphene oxide [65] characterized by various oxygen moieties (Figure 6), which in turn can be chemically converted to reduced graphene oxide (rGO), with restoration (albeit partially) of the sp^2^ carbon configuration in the graphene state.

This method is unique and attractive because single-layer graphene can be produced on a large scale and at a relatively low cost [67]. Graphene oxide is therefore an important precursor that can be obtained from graphite using suitable oxidizing agents, which mainly consist of KMnO_4_ and NaNO_3_ in H_2_SO_4_ solution according to the well-known Hummers’ method developed in 1958 [68].

However, toxic gases such as NO_2_ and N_2_O_4_ are released during this highly efficient procedure, and the residual Na^+^ and NO^3^- ions are difficult to remove from the wastewater formed from both the GO synthesis and purification processes.

In 2010, Marcano et al. [69] eliminated NaNO_3_ and introduced H_3_PO_4_ in the oxidizing system since graphene oxide nanoribbons were obtained from multiwalled carbon nanotubes and KMnO_4_ with more intact graphitic basal planes upon addition of H_3_PO_4_ [70].

**Figure 7:**
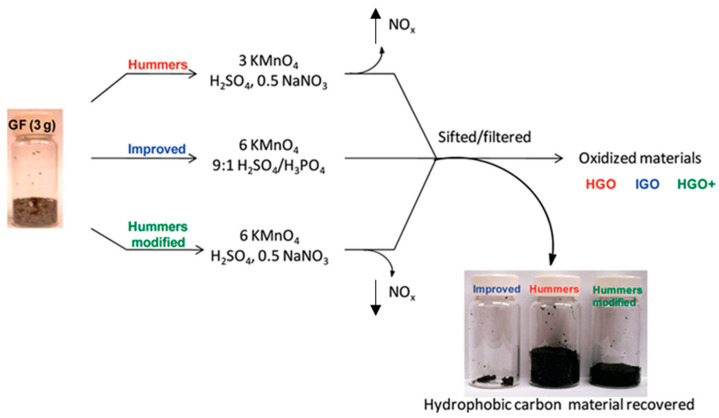
Representation of the procedures followed, starting with graphite flakes. Images of the oxidized hydrophobic carbon material collected during purification using IGO, HGO, and HGO+ (in the glass bottles) are presented. Reprinted with permission from [69]. Copyright {2010} American Chemical Society.

An oxidation procedure for graphite flakes was developed using a larger amount of KMnO_4_ and a 9:1 mixture of concentrated H_2_SO_4_/H_3_PO_4_ (Figure 7). This is called the “improved method”, giving “improved” graphene oxide (IGO); graphene oxide was also prepared using the classical Hummers’ method (HGO) and a modified Hummers’ method (HGO+).

As can be seen in the figure, a very small amount of under-oxidized material is produced with the IGO method, indicative of its increased efficiency with respect to the other methods. Moreover, the reaction is not largely exothermic, and no toxic gas is produced. This IGO-generated product is more oxidized and possesses a more regular structure than the other materials produced using HDO and HGO+.

**Figure 8:**
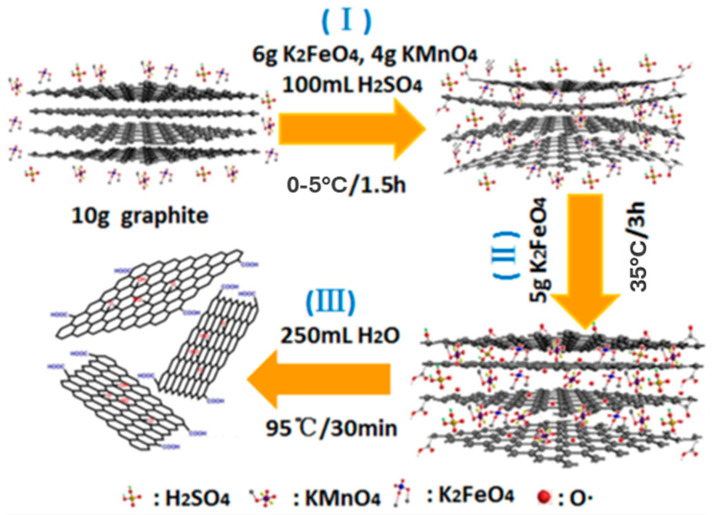
Illustration of the preparation of GO based on a newly improved Hummers’ method from [76].

Besides the use of a greater amount of KMnO_4_ instead of NaNO_3_ [71,72], further modification of Hummers’ method was accomplished by introducing a pre-oxidation step before KMnO_4_ oxidation (in the absence of NaNO_3_) [73] or by replacing KMnO_4_ with K_2_FeO_4_ while NaNO_3_ was removed [74,75]. Yu et al. [76] addressed the problems of high consumption of oxidants, intercalating agents, and time-consuming procedure by partly replacing KMnO_4_ with K_2_FeO_4_ and using a smaller amount of concentrated sulfuric acid, according to the scheme in Figure 8, offering new possibility for the production of GO in an economical, eco-friendly, and efficient way.

**Scheme 1:**
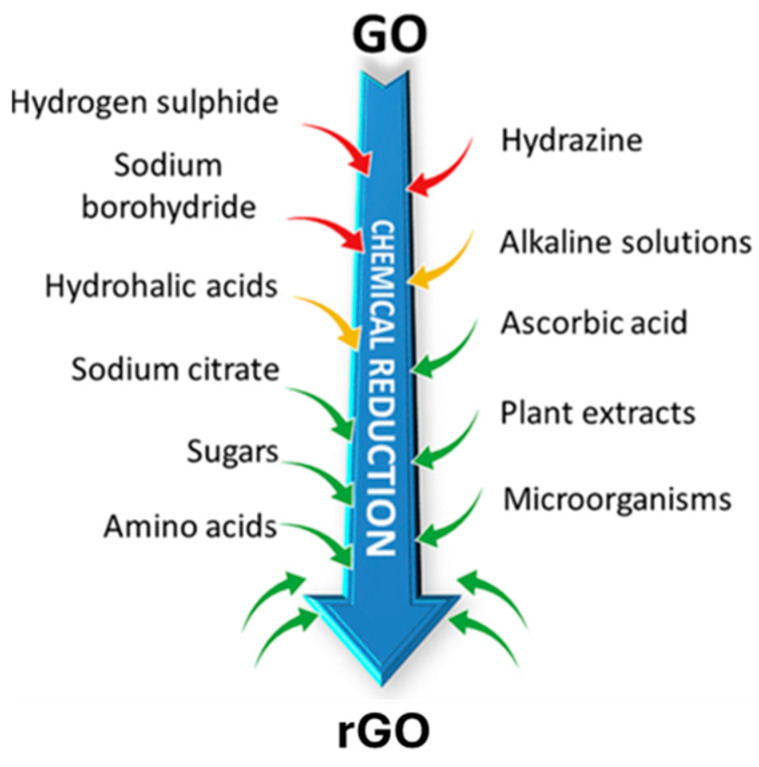
Schematic representation of the major methods of oxidation of graphite to graphene oxide and the chemical reduction of graphene oxide by some reductants from [82]. © 2017 Elsevier Ltd. (2017).

Such a thorough study on graphene oxide preparation is strictly related to its important role as a starting material in the production of large quantities of rGO, which can also be considered as chemically derived, converted, or modified graphene closely resembling pristine graphene in both structure and properties [77]. Reduced graphene oxide is obtained via direct reduction of GO through both chemical and thermal methods: the removal of the oxygen moieties is a vital topic that determines the properties of the ultimate product [78]. The reduction efficiency largely depends on the methods and processing parameters, resulting in graphene with various properties [77,79,80]. H_2_S was first used as a reducing agent in 1934 [81]; since then, a large number of reductants (Scheme 1) [82] have been used, among which hydrazine gave rGO with particularly improved electrical and structural properties, resembling pristine graphene to a large extent [83].

In addition to hydrazine [84,85], hydrogen sulphide [86] and metal borohydrides such as NaBH_4_ [87,88], hydrohalic acids [84,85,89,90,91,92], and various alkaline solutions [93,94,95] are used as reductant agents.

However, to reduce toxic chemicals and complicated manufacturing procedures, researchers introduced green reduction agents comprehending sugars (saccharides) [96,97,98], ascorbic acid (Vitamin C) [96,97,98,99,100], and micro-organisms such as bacteria [101,102], as well as amino acids [103,104,105,106] and plant extracts [107,108,109,110]; ascorbic acid, in particular, has produced rGO with electrical conductivity up to 800 Sm^−1^, which is almost comparable to that of rGO prepared using hydrazine [111].

Mature alternatives to wet-chemical reduction methods are represented by the solvothermal process as well as the hydrothermal process; they offer the opportunity to modify the physico-chemical properties of the solvent and overwhelmingly address problems of scalability and environmental concerns [112].

The hydrothermal technique has been largely used in the reduction of graphene oxide because it offers the advantages of being a water-soluble reaction, and the ionic product of water is high. Under hydrothermal conditions, the protonation of ·OH functionalities originates dehydration on the edges or basal planes of GO, followed by the π-bonding restoration [113,114,115,116,117,118,119,120].

**Figure 9:**
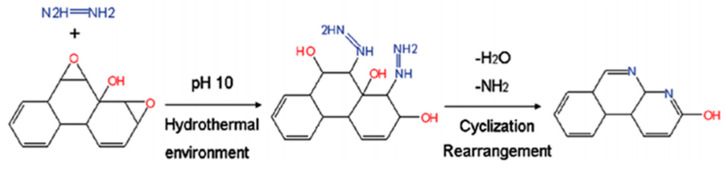
Ring opening of epoxides induced by hydrazine reactions and incorporation of N in the aromatic skeleton. Adapted with permission from [121]. Copyright {2010} American Chemical Society.

In 2010, Long et al. [121] prepared nitrogen-doped graphene sheets through hydrothermal reduction of colloidal dispersions of graphite oxide in the presence of hydrazine and ammonia at a pH of 10 via multiple reactions such as ring opening of epoxides induced by hydrazine as well as reorganization of the carbon skeleton with incorporation of nitrogen (Figure 9).

The reduction process can also be achieved by thermal treatment (thermal annealing) in inert or reductive atmospheres at temperatures above 200 °C [122,123,124,125,126]; with this method, oxygen functionalities are removed in the form of water, carbon dioxide, and carbon monoxide.

Thermal annealing is a well-established process for the preparation of heteroatom-doped graphene using GO and suitable molecules as the heteroatom source. Sheng et al. [127] reported a facile and catalyst-free approach for the synthesis of nitrogen-doped graphene by thermal annealing of graphite oxide with a low-cost industrial material, melamine, as a nitrogen source.

**Scheme 2:**
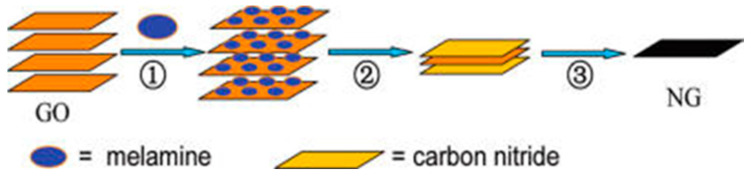
Illustration of the nitrogen doping process of melamine into graphite oxide layers, adapted with permission from [127]. Copyright {2011} American Chemical Society.

The process reported in Scheme 2 consists of the adsorption of melamine into graphite oxide layers at T < 300 °C (1); formation of carbon nitride at T < 600 °C (2), and decomposition into graphene layers at T > 600 °C (3).

Such methods can also be extended to prepare other heteroatom (such as B, P, S, Se, Si and I)-doped graphene nanosheets if appropriate precursors are used, e.g., boron oxide (B_2_O_3_), benzyl disulfide, and triphenyl phosphine as the sources of B, S and P elements, respectively [128], diphenyl diselenide [129], triphenyl silane for Si [130] and I_2_ for I [131].

This methodology has been largely applied to synthesize suitable metal-free graphene-based ORR electrocatalysts. It takes advantage of having different compounds as heteroatom sources and regulating the temperature and atmosphere to conveniently introduce the heteroatom into graphene in order to modulate the electrochemical properties.

### 2.2. Bottom-Up Graphene Synthesis

The typical “bottom-up” production techniques are arc discharge, CVD on active metals, hydro-/solvothermal synthesis, on-surface synthesis, laser irradiation, and pyrolysis of carbon sources.

In order to obtain high-quality graphene on a large scale under controllable conditions, the arc discharge technique used to vaporize elemental carbon to form nanotubes [132], fullerenes [133], and onion structures [134] has been also applied for synthesizing graphene sheets [135].

**Figure 10:**
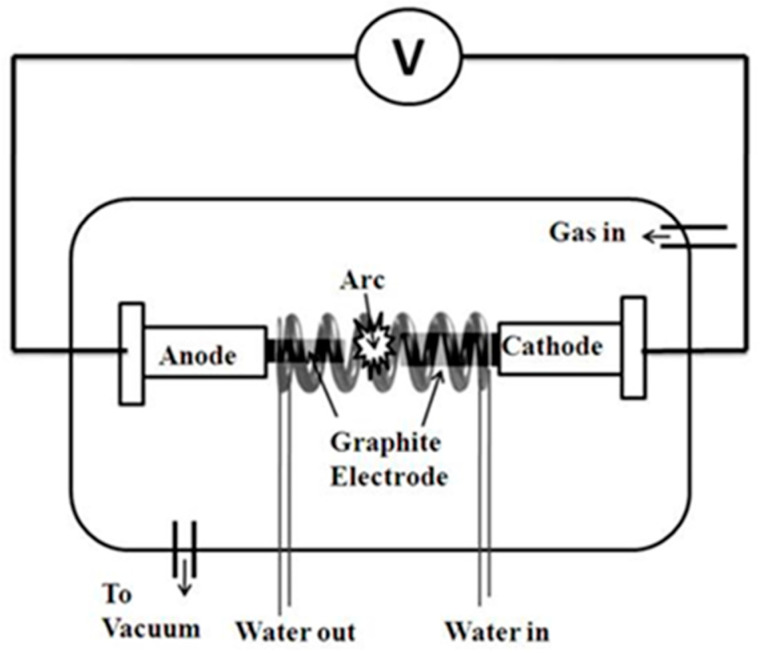
Schematic diagram of the DC-arc discharge set-up [136].

In the classic arc discharge set-up illustrated in Figure 10 [136], an arc is generated between two graphite rods upon application of a high voltage, with the anode sprayed up to the atomic state. Formation of carbon structures occurs via the condensation of carbon vapor cooled in a buffer gas.

Separation of the graphite layers into graphene sheets proceeds via high-temperature exfoliation, or the graphite layers can even get broken into small clusters/atoms, which then form graphene sheets via catalytic growth, demonstrating that two mechanisms exist in an arc discharge process simultaneously.

Among the process parameters of arc discharge (arc current/gap voltage, type and ratio of buffer gas, ambient media, cooling conditions, external fields and catalysts) influencing graphene formation in terms of layer numbers, purity, crystallinity, morphology, etc., investigations have been carried out on the buffer gas since arc plasma is enhanced by the ionization of buffer gas.

The use of H_2_, Ar, CO_2_, air, NH_3_, O_2,_ or various mixtures [135,137,138,139,140,141,142,143] as buffer gases could produce graphene sheets of integral structure, high thermal stability, and good dispersibility in organic solvents [142,144].

First studies were conducted using hydrogen (pure or mixed with He, N_2_ or, Ar) evidencing its key role in the formation of graphene and its etching effect on amorphous carbon; in arc discharge, the dangling carbon bonds are terminated with hydrogen, the rolling of sheets into nanotubes and graphitic polyhedral particles is minimized, and hydrogen also imparts a high temperature on plasma with in situ defect-healing effect [145,146,147].

Carbon dioxide mixed with helium is a better and safer buffer gas. Wu et al. [135] produced tens of grams of high-quality graphene sheets with four to five layers in minutes under optimized conditions consisting of a low voltage (<35 V), high buffer gas pressure (>1270 Torr), high current (about 150 A), and 25–40% (*v*/*v*) CO_2_ in the helium–carbon dioxide mixture. Moreover, the graphene sheets showed excellent solution processability, makingpossible to directly fabricate a variety of films using the organic solutions as precursors.

Arc discharge synthesis was run in liquid media, too, such as liquid nitrogen [148] and water [149,150], which also allows the collection of graphene sheets floating on the water surface.

Other implementations of the arc discharge synthesis in promoting the nucleation, growth, and separation of graphene were obtained by application of external fields [138,151,152,153] or using catalysts such as ZnO, ZnS, Cu [154,155], TiO_2_ [155], and Ni–Y_2_O_3_ powder [152].

Liu et al. [154] combined flake graphite as a carbon source, Cu as a catalyst, and melamine as a nitrogen source and simultaneously synthesized N-doped graphene sheets with nitrogen content as high as 4.92 at% and undoped graphene sheets in the different parts of the apparatus according to two different mechanisms: catalytic growth mechanism for the cathode-part graphene sheets and an exfoliation mechanism for the wall-part graphene sheets.

Since the first arc discharge synthesis of B and N-doped graphene was reported by Panchakarla et al. [156], this technique has also been used to introduce fluorine [157] and silicon [158] as heteroatoms into the graphene lattice.

Another important technique widely used to obtain large-area, single-crystalline graphene is Chemical Vapor Deposition. In a typical CVD process, a hydrocarbon in the gas phase is introduced as a carbon source for graphene growth; hydrogen is usually required to achieve high-quality graphene [159]. Decomposition and deposition usually occur on transition metal foils (Ni or Cu) [160,161,162,163,164,165] that act as catalysts and supports [165]. The carbon solubility of the catalyst is an important factor for controlling the number of graphene layers, enabling different growth mechanisms (carbon adsorption, absorption, or segregation). In the case of Cu, its low carbon solubility, also at high temperatures, allows the growth of single-layer graphene through an adsorption mechanism.

**Figure 11:**
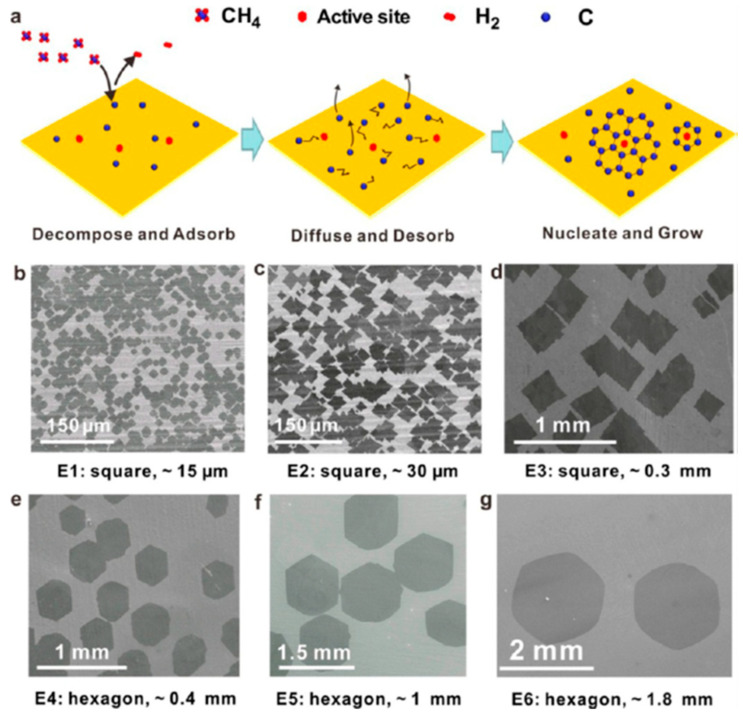
Illustration of the Cu-based graphene growth mechanism and graphene samples grown under different conditions. (**a**) Scheme for the Cu-based graphene growth mechanism: red hexagons are used to symbolize the active sites of the Cu surface, and blue spots signify active carbon species (CH_x<4_). (**b**–**g**) Typical scanning electron microscopy (SEM) images of graphene synthesized under different growth conditions: (**b**) (E1), (**c**) (E2), (**d**) (E3), (**e**) (E4), (**f**) (E5), and (**g**) (E6). Reprinted with permission from [166]. Copyright {2012} American Chemical Society.

Given the importance of producing large single crystals for the implementation of graphene into practical applications, the nucleation growth, ∼106 nuclei/cm^2^ in conventional cases, needs to be significantly decreased [162]. Yan et al. [166] conducted an interesting study where, by constructing a controlled chamber pressure CVD, it was possible to grow single crystal monolayer graphene up to ∼4.5 mm^2^ on commercial Cu foils from methane and hydrogen; they also showed that Cu pretreatments, electrochemical polishing, and high-pressure annealing are critical for suppressing graphene nucleation site density. The growth mechanism for Cu-based graphene was explored by studying the influence of varied growth parameters on graphene domain sizes (Figure 11).

In the proposed mechanism, the active carbon species from the dissociated CH_4_ are apt to agglomerate into thermodynamically stable (C_n_H_y_)_s_ species on the active sites of the Cu surface, ultimately leading to the formation of graphene nuclei. Once the graphene nuclei are formed, most of the active carbon species are captured and consumed for the growth of graphene, and the growth of larger-size graphene domains during the extended growth time will be enabled.

Another strategy for decreasing the nucleation growth is the passivation of Cu surface active sites by utilizing a growth–etching–regrowth strategy or O_2_ pretreatment prior to growth [167,168,169,170,171,172,173] so that centimeter-sized single-crystalline graphene can be grown [170].

The CVD process has also been revealed to be an effective approach for achieving substitutional doping without affecting the crystalline nature. N-doping was obtained by introducing gaseous NH_3_ [174,175,176,177,178,179,180] or N_2_ [181,182,183,184] in the CVD apparatus. Qu et al. [185] prepared large N-doped graphene films, ca. 4 cm^2^ in size, by flowing a nitrogen-containing reaction gas mixture (NH_3_:CH_4_:H_2_:Ar = 10:50:65:200 standard cubic centimeters per minute) at 1000 °C over a Ni-coated SiO_2_/Si wafer. These N-doped graphene films were shown for the first time to have ORR electrocatalytic properties, which determined the development of the CVD technique for the preparation of metal-free graphene ORR electrocatalysts.

As an alternative to ammonia and nitrogen gases, liquid N-containing substances, such as acetonitrile [186], pyridine [187,188,189,190,191], DMF [192,193], urea [194,195], various amines [196,197], and solid species such as melamine [198] and triazine [199], were used as a nitrogen source.

Xue et al. [189] demonstrated a rational route based on self-assembly of pyridine molecules on Cu surface at temperatures as low as 300 °C, producing single-layer, single-crystal, tetragonal-shaped, N-doped graphene domain arrays with typical n-type field-effect transistors (FETs) for N-doped graphene both in air and vacuum and mobilities in the range of 53.5–72.9 cm^2^V^−1^s^−1^.

Analogously, B-doped graphene films can be obtained from gaseous boron sources such as diborane [200] and solid sources such as boric acid [194]. In the last case, Wu et al. [194], by easily adjusting the amount of boric acid and polystyrene as boron and carbon sources, respectively, modulated the boron content from 0.7 to 4.3%, obtaining boron-based graphene FET devices with a mobility of 450–650 cm^2^V^−1^s^−1^.

Boric acid was also used to produce N,B-doped graphene. Bepete et al. [184], using a single-step CVD route with methane, boric acid powder, and nitrogen gas, synthesized large-area graphene, incorporating small BN domains in a facile and safe process that avoided the use of boranes and ammonia. XPS studies evidenced that BN species were incorporated in the graphene in small BN domains where the N in the BN is bonded to carbon, forming B–N–C systems.

The CVD technique was also used to incorporate phosphorus atoms [201,202] in the graphene lattice by using solid triphenylphosphine and sulfur [203,204] via simple and efficient CVD methods. In particular, Hassani et al. [204] grew S-doped graphene with high sulfur content (5 at%, determined by XPS) from sulfur powder and acetylene gas at 600 °C. They produced non-porous sulfur-doped graphenes with high surface areas, where sulfur atoms are successfully incorporated into the carbon matrix of samples with covalent bonds, as revealed by Raman and XPS analyses.

In the synthesis of heteroatom-doped graphene, hydro- and solvothermal processes have been revealed to be proper bottom-up, straightforward synthesis methodologies.

N-doped graphene was prepared by Deng et al. [205] by reacting lithium nitride with tetrachloromethane at 250 °C for 10 h in a stainless-steel autoclave in the presence of nitrogen (sample NG^−1^) or at 360 °C in the presence of cyanuric chloride (sample NG-2), obtaining N-graphene with nitrogen species content in the range of 4.5–16.4%. Preliminary tests of NG^−1^ and NG-2 as ORR electrocatalysts showed that they are more active than pure graphene and commercial XC-72, while NG-2 exhibits a higher activity than NG^−1^ because of higher N species content.

**Scheme 3:**
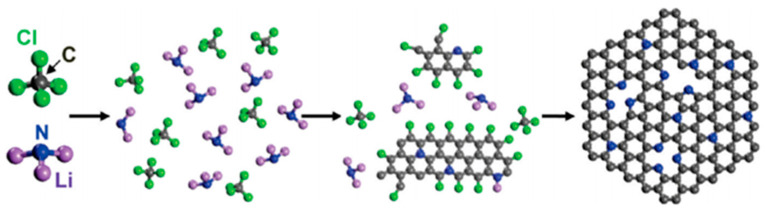
Scheme of a proposed mechanism for solvothermal synthesis of N-doped graphene via reaction of CCl_4_ and Li_3_N, where gray balls represent C atoms, blue represent N, green represent Cl, and purple represent Li. Reprinted with permission from [205]. Copyright {2011} American Chemical Society.

It has also been proposed, for graphene synthesis using lithium nitride in tetrachloromethane at 250 °C, a reaction mechanism that assumes that the graphene structure derives from mutual coupling of intermediates originating from transformation of sp^3^-hybridized carbon in CCl_4_ to sp^2^-hybridized carbon, such as dichlorocarbene, free -C=C-, and -C=N- groups. The small domains of sp^2^-hybridized carbon containing N are obtained through further dechlorination and then grow into N-doped graphene sheets (Scheme 3).

The strategy of using CCl_4_ as solvent and as carbon source at the same time has also been successfully applied in the solvothermal synthesis of B-doped graphene [206] and dual-doped N,X (X = B, P, or S) graphene [207,208].

**Figure 12:**
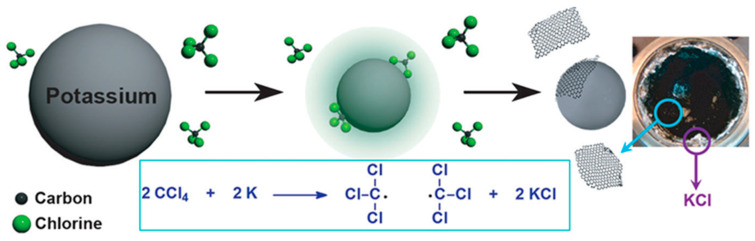
Formation of C-graphene by solvothermal reaction between CCl_4_ and potassium. The photograph is of the autoclave after the reaction, showing the formation of C-graphene (black) and potassium chloride (KCl; white) [207] © 2014 Wiley-VCH Verlag GmbH & Co. KGaA, Weinheim.

The fundamental step is the Wurtz-type reactive coupling reaction of CCl_4_ with potassium, which involves a bottom-up process for rapid preparation of high-quality pristine graphene [207]. In such a reaction, carbon radicals and potassium chloride are formed, then the carbon radicals form carbon–carbon (C-C) bonds on the surface of potassium particles to give graphene (Figure 12).

Doping of graphene occurred as the above-mentioned doping agents were present in the autoclave.

As an alternative to using alkaline metals, magnesium powder was used for the hydrothermal synthesis of N-doped graphene from hexamethylenetetramine, which was pyrolyzed in water at 500 °C for 20 h [209].

Another synthetic method starting from molecular precursors is the on-surface synthesis of porous graphene, which results in the collection of graphene-related materials with nanopores in the plane [210,211].

Thanks to the fabrication of regular two-dimensional polyphenylene networks with single-atom-wide pores and sub-nanometer periodicity by Bieri et al. [212], it was possible to develop a process based on surface-assisted coupling (generally Ullman reaction) and cyclodehydrogenation of specifically designed molecular building blocks consisting of variously condensed and covalently bonded benzene-ring-based systems [213,214].

This method generally produces graphene nanoribbons that undergo cross-dehydrogenative coupling at high temperature to obtain atomically precise nanometer-sized pores [215].

**Figure 13:**
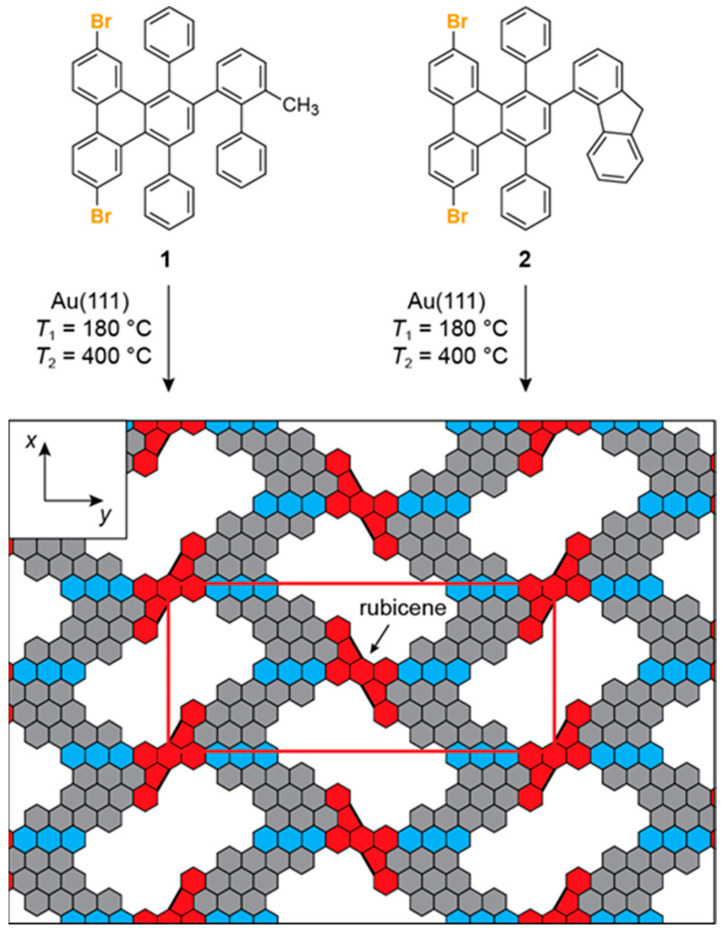
Schematic representation of the bottom-up synthesis of C-NPG from molecular precursors 1 and 2 (anti-configuration) and structure of the C-NPG anti-configuration with the interface (rubicene) groups highlighted in red and the 2D unit cell indicated by a rectangle. Reprinted with permission from [216]. Copyright {2020} American Chemical Society.

Jacobse et al. [216] created fully conjugated nanoporous graphene (C-NPG) through a single, mild annealing step following initial polymer formation by depositing methyl and methylene-bearing precursors 1 or 2 (Figure 13) onto a Au(111) substrate and annealing at 400 °C. This new 2D material emerges due to the reactivity of methyl and methylene crosslinking handles toward cross-dehydrogenative coupling at low temperatures.

The on-surface process also allows the formation of low-dimensional carbon-based nanostructures containing periodic nonplanar pores [217].

**Figure 14:**
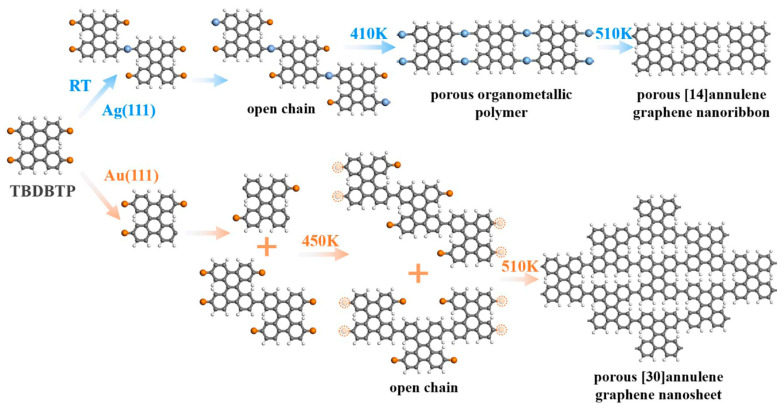
Reaction pathway for the formation of 1D porous graphene nanoribbons on Ag(111) and 2D porous graphene nanosheets on Au(111), starting from TBDBTP precursors. Color code: Ag, blue; Br, orange; C, gray; H, white [218] © 2023 WILEY-VCH Verlag GmbH.

Qin et al. [218] recently selectively synthesized one-dimensional graphene nanoribbons containing [14]annulene pores on Ag(111) and two-dimensional graphene nanosheets containing [30]annulene pores on Au(111) starting from the same precursor TBDBTP (Figure 14).

They showed that (1) the formation of 1D graphene nanoribbons containing [14]annulene pores on Ag(111) is a thermodynamically controlled pathway due to the reversibility and flexibility of C−Ag−C-linked organometallic intermediate motifs; (2) the formation of 2D graphene nanosheets containing [30]annulene pores on Au(111) is a kinetics-driven pathway where the debromination process is the rate limiting step for the initial C−C coupling reaction.

Another technique that has gained much attention for the construction of 3D porous graphene structures is based on irradiation of certain carbon materials with a laser beam: the obtained laser-induced graphene (LIG) is characterized by high porosity, excellent electrical conductivity, and good mechanical flexibility.

Since the first conversion of a commercial polyimide (PI) film into 3D porous laser-induced graphene (LIG) under ambient conditions using a mid-infrared (MIR) CO_2_ laser system by Lin et al. [219], there has been significant development in the application of lasers to synthesize carbonaceous materials and in particular LIG as an alternative to conventional thermal treatment. Most types of lasers generate a photothermal effect, i.e., target materials absorb the incident photon energy and convert it into thermal energy [220]. The laser irradiation leads to a sharp rise in the localized temperature (>2500 °C), which can cause the breakage and recombination of weak chemical bonds, such as –COOH and –OH [221], while releasing gaseous products. In the PI network, the C-O, C=O, and N-C bonds are broken, and the aromatic compounds rearrange to form graphitic structures.

PI is commonly used as a precursor because its chemical structure is particularly well suited for LIG formation: it has a high content of aromatic sp^2^ carbons, which are more likely to form the hexagonal graphene structure than other organic sources that only produce amorphous carbon [222].

**Figure 15:**
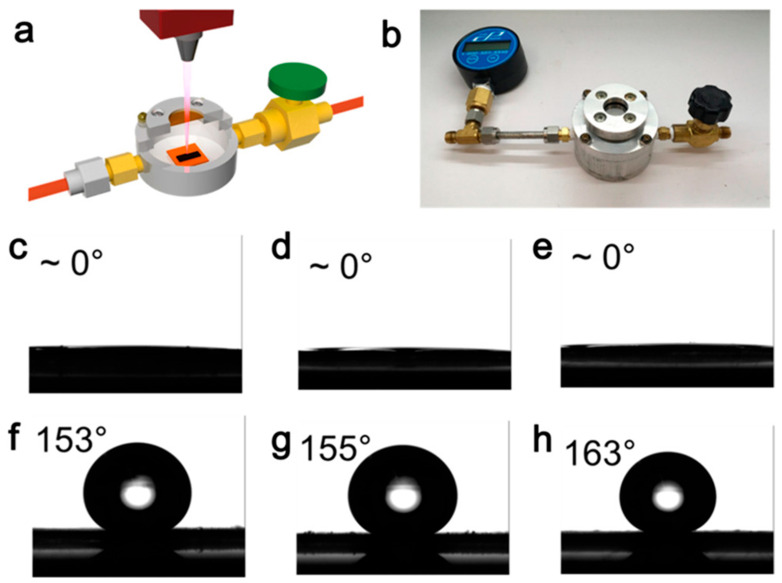
LIG formation under controlled atmosphere. (**a**) Scheme and (**b**) photograph of the apparatus for the fabrication of LIG under a controlled atmosphere. Contact angles of LIG prepared in (**c**) ambient air, (**d**) air (chamber), (**e**) O_2_ (chamber), (**f**) Ar (chamber), (**g**) H_2_ (chamber), and (**h**) SF_6_ (chamber). Reprinted with permission from [224]. Copyright {2018} American Chemical Society.

By lasing PI under various gas environments, such as O_2_, Ar, and SF_6_ under various atmospheres, Li et al. [223] prepared LIG with different surface morphologies and chemical compositions: under ambient or oxidizing atmospheres, LIG with superhydrophilic surface is obtained (contact angle∼0°, Figure 15c–e), while a superhydrophobic surface (contact angle > 150°) can be obtained when inert or reducing gas is applied.

In addition to PI, various materials have been successfully used to prepare LIG, including polysulfones [225], cloth, paper, food [226], wood [227], lignin [228], and phenolic resin [229].

Another synthetic aspect to be taken into account is the laser type (UV, IR, or visible lasers), since the laser properties must match the adsorption properties of the carbon precursor for effective energy transfer and desired outcomes, considering that many carbon precursors exhibit strong light absorption in the UV, visible, and MIR regions. UV lasers primarily drive the reaction through photochemical processes; because of the high energy involved in breaking molecular bonds directly with minimal thermal contribution, a less uniform and more defect-rich graphene structure can be obtained with UV lasers. Conversely, IR lasers rely on photothermal effects, as described above, with the formation of graphene with more uniform structural properties. Visible lasers harness both photothermal and photochemical mechanisms, offering a balance between direct bond cleavage and thermal decomposition, thus affecting the crystallinity and uniformity of the resulting graphene [230].

Moreover, laser processing in a special gas, liquid, or solid environment that contains the target atoms allows the heteroatom-doping of LIG [221]; in particular, in situ doping methods, which offer benefits such as precise control over dopant distribution and enhanced stability, can be performed in two ways. The first consists of lasing a PI film, coated with a thin dopant layer obtained by solvent evaporation of a dopant slurry on the PI film. The second involves the formation of a homogenous solution of the PI precursor solution, typically poly(amic acid) (PAA) with dopants, before laser irradiation, which occurs after thermal curing to create a PI film [231,232,233].

**Figure 16:**
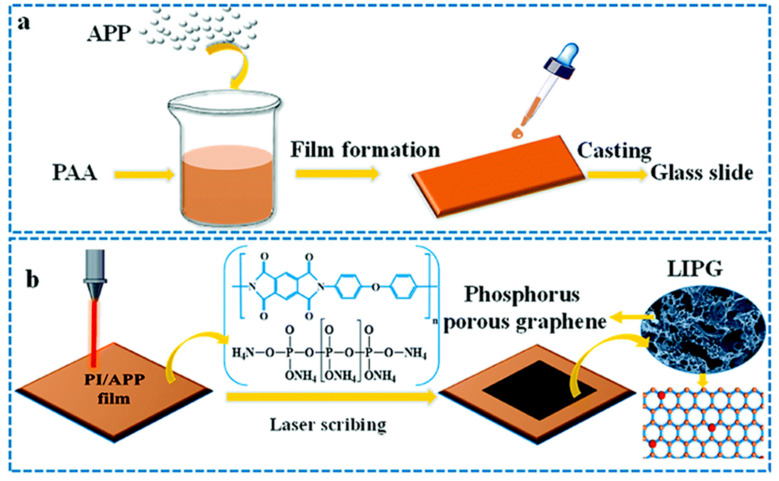
Schematic of the formation of LIPG from the PI/APP film. (**a**) Preparation of the PI/APP film from PAA containing the APP solution; (**b**) LIPG fabricated on PI/APP film by laser induction [233].

Yang et al. [233] developed a one-step method for selectively generating phosphorus-doped hierarchically porous graphene via 10.6 μm CO_2_ laser induction (LIPG) with significantly improved electrochemical performance, innovatively using ammonium polyphosphate (APP) as a flame retardant and P supplier as well (Figure 16).

The last bottom-up method is pyrolysis, where decomposition of suitable carbon-based precursors is brought about by high temperatures, which can also be achieved using plasma-based heating methods [234,235].

Pyrolysis is largely used because of the great availability of carbon sources, particularly chemical products such as carbonaceous salts [236,237,238], synthetic polymers [239], and plastic wastes [239].

**Figure 17:**
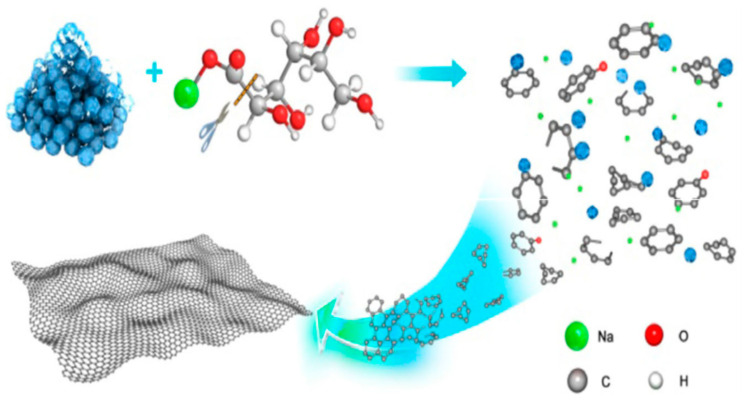
Schematic outline of the general solid-state pyrolytic conversion strategy for the preparation of monolayer graphene. The blue spheres represent the Na_2_CO_3_ salt, the large green sphere represents Na^+^ in the solid sodium carboxylate precursor, and the small green spheres represent the Na_2_CO_3_ crystals generated in situ; reprinted with permission from Springer Nano Research©Tsinghua University Press and Springer-Verlag Berlin Heidelberg 2017.

Zhu et al. [236] developed a novel general synthesis strategy based on the direct pyrolytic conversion of sodium carboxylate in the presence of Na_2_CO_3_ to monolayer graphene (Figure 17).

Another important group of carbon precursors is organic species such as sugar [240], organic natural polymers such as lignin [241], and more generally biomass precursors consisting of natural sources such as plants or wood parts, agricultural waste, fruits, vegetables, biopolymers, or even insect parts [242].

**Figure 18:**
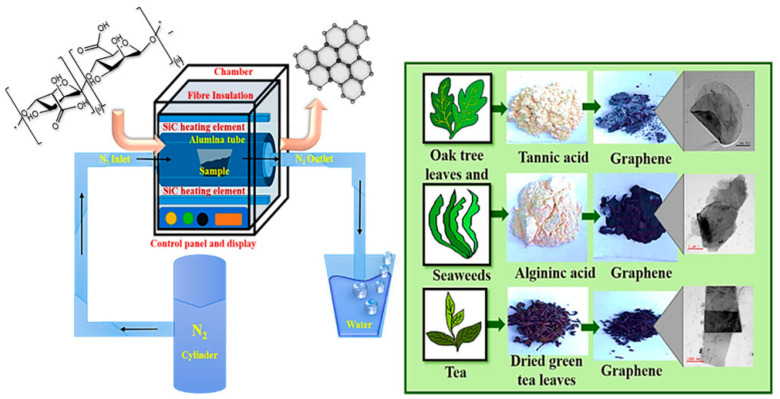
Pyrolytic apparatus and green materials used for the preparation of graphene [243].

Roy et al. [243] produced graphene from nonconventional sources such as tannic acid, alginic acid, and green tea using a controlled pyrolysis technique (Figure 18).

The process of graphitization of the precursors was thoroughly studied, and it was found that the temperature needed is 1100 °C.

Moreover, the pyrolysis technique is also very useful for the synthesis of heteroatom-doped graphene [244]; for instance, starting from a low-cost crystal sugar, Pan et al. [240] developed a simple strategy for fabricating nitrogen-doped graphene (NG) as efficient metal-free ORR electrocatalysts via one-step pyrolysis of sugar in the presence of urea.

## 3. Characterization of Graphene

Effective characterization techniques play an important role in accurately analyzing the surface morphology, the structural features, composition, and electrochemical behavior of the graphene-based ECs.

In particular, since the electrochemical performance and durability of metal-free graphene-based ECs are strongly dependent on the structure and morphology of the materials, this section will describe techniques based on (1) Electron Microscopy (Transmission Electron Microscopy—TEM; High Resolution Transmission Electron Microscopy—HRTEM; Scanning Electron Microscopy—SEM; and Scanning Tunneling Electron Microscopy—STEM); (2) Scanning Probe Microscopy (Atomic Force Microscopy (AFM) and Scanning Tunneling Microscopy (STM)); (3) Raman Spectroscopy; (4) X-ray Photoelectron Spectroscopy (XPS); (5) X-ray Diffraction (XRD); (6) UV−Vis Spectroscopy; (7) X-ray Absorption Near-Edge Structure (XANES).

### 3.1. Electron Microscopy (TEM, HRTEM, SEM, and STEM)

The fundamental understanding of the structural characteristics of a nanocomposite is significant for building an optimal nanocatalyst to maximize its electroactivity. SEM has been largely used to unravel the morphology of graphene, and TEM has been used to image nano-sized materials at atomic scale resolutions.

**Figure 19:**
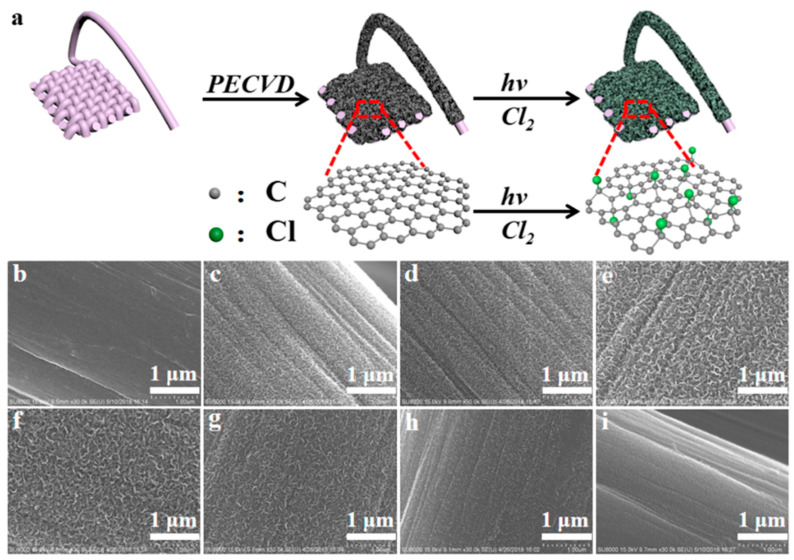
Preparation scheme and SEM images of chlorinated VG. (**a**) Schematic illustration of VG grown on CC using the PECVD method, and subsequent chlorination. SEM images of (**b**) CC; (**c**) pristine CC@VG; (**d**) chlorinated CC@VG for 2 min; (**e**) chlorinated CC@VG for 4 min; (**f**) chlorinated CC@VG for 6 min; (**g**) chlorinated CC@VG for 8 min; (**h**) chlorinated CC@VG for 12 min; and (**i**) chlorinated CC@VG for 20 min, from [245]. © 2019 Elsevier Inc. (2019).

Zhang et al. [245] illustrated accurately via SEM the photochlorination of vertically oriented graphene (VG) grown on carbon cloth (CC), indicated as CC@VG, with chlorination time (Figure 19).

**Figure 20:**
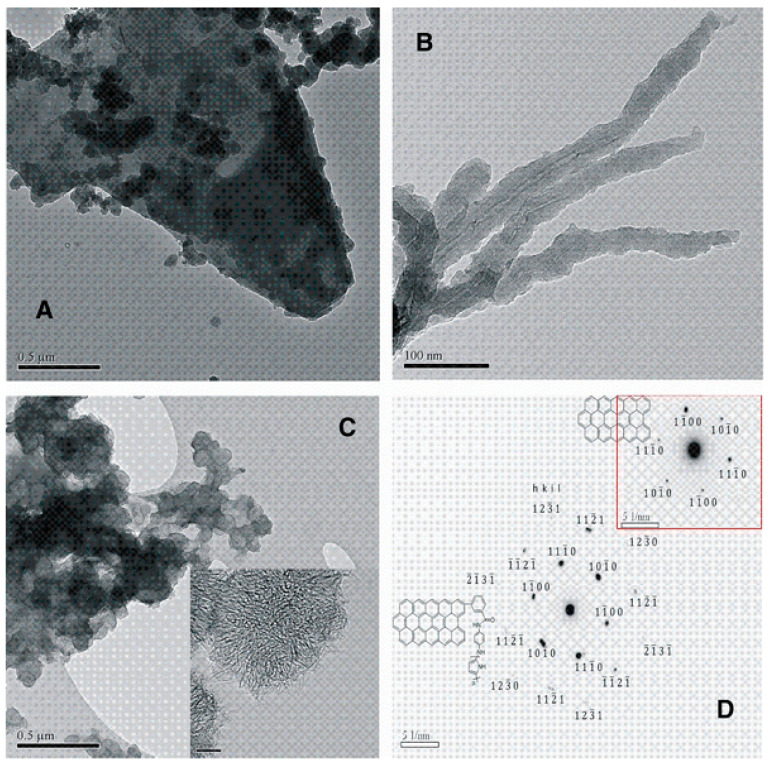
TEM image of the (**A**) Graphene-g-PPy; (**B**) mWC_nT_-g-PPy; (**C**) carbon nanohorn-g-PPy; (**D**) and the selected area electron diffraction (SAED) patterns of pure graphene (**right**, **top**) [246].

Interesting TEM observations of carbon allotropes [graphene, multi-walled carbon nanotubes (mWC_nT_) and nanohorns] that are covalently bound to polypyrrole (PPy) (Graphene-g-PPy, mWC_nT_-PPy, carbon nanohorn-g-PPy) (Figure 20) [246] showed that, for chosen carbons, the polymer coating looks completely different; in particular, in the graphene-based system (Figure 20A), spherical particles of the polypyrrole are evenly distributed on the carbon surface and the size of polymers is in the range 20–80 nm.

However, TEM is characterized by limited resolution (0.2 nm size uncertainty) at low operating voltage, while at high voltage, melting effects of the smallest nanoparticles under the electron beam can occur with damage to the monolayers. Therefore, a new class of TEM (High-Resolution TEM, HRTEM) has been introduced, which, in combination with a monochromator via aberration correction, can provide 1 Å resolution at an acceleration voltage of only 80 kV [247,248]. Using electrons at 80 kV not only minimizes the knock-on damage but also improves detection sensitivity due to the higher scattering power of carbon at a lower acceleration voltage of the electrons [249]. HRTEM, therefore, permits the study of the size and crystalline structure of relatively small metal nanocrystals; it can resolve the in-structural features of graphene (individual carbon atoms, defects, adatoms, etc.) [250] and the number of layers of graphene flakes can be detected from the diffraction of the electron nanobeam [249].

**Figure 21:**
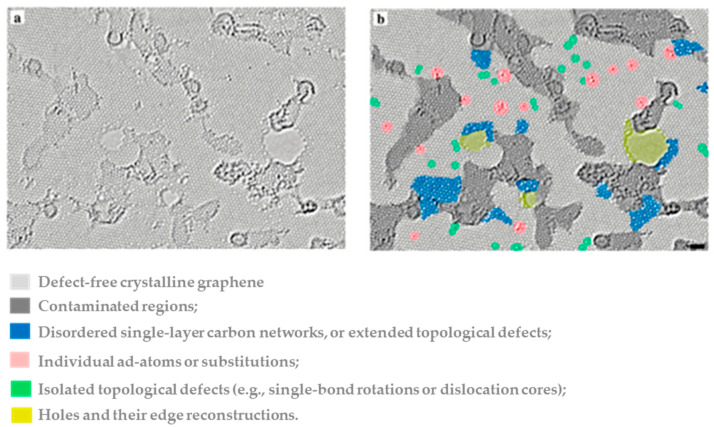
Atomic resolution, aberration-corrected TEM image of a single-layer reduced-graphene oxide membrane. (**a**) Original image and (**b**) with color added to highlight the different features. Adapted with permission from [247]. Copyright {2010} American Chemical Society.

A very nice example of HRTEM potentialities is offered by Gómez-Navarro et al. [247], who were able to provide insight into the exact atomic structure of the rGO layers. At the atomic resolution, an aberration-corrected TEM image of a single-layer reduced-graphene oxide membrane (Figure 21) showed the hexagonal lattice of the well-crystallized graphene sheet, as well as various defects and deformations, e.g., disordered carbon networks, trapped carbonaceous adsorbates, heavy atoms, isolated pentagon−heptagon pairs, and clustered defects evidenced by the different colors in Figure 21.

However, HRTEM may have the drawback of generating defects and edge reconstructions due to the high-energy electrons [245,251].

Investigation of morphology, crystal structure, and composition distribution has also been achieved using STEM [252], which combines the principles of transmission electron microscopy and scanning electron microscopy.

Its primary advantage over conventional TEM is the possibility of using other signals that cannot be spatially correlated in TEM, including secondary electrons, scattered beam electrons, characteristic X-rays, and electron energy loss, while its primary advantage over conventional SEM imaging is the improvement in spatial resolution. In particular, the imaging method in STEM, based on collecting scattered electrons with a dark-field detector both in medium (ADF) and high-angle aberration-corrected annular configuration (HAADF), allows characterization at the atomic level.

**Figure 22:**
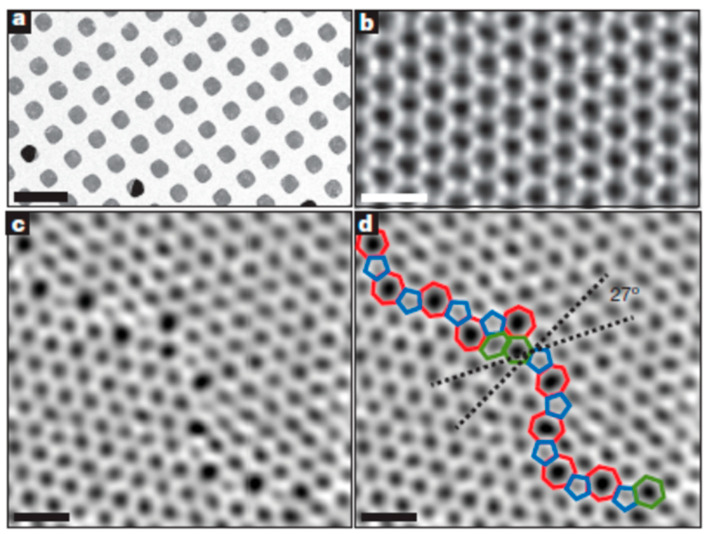
ADF-STEM images of graphene crystals. (**a**) SEM image of graphene transferred onto a TEM grid with over 90% coverage using novel, high-yield methods. Scale bar, 5 μm. (**b**) ADF-STEM image showing the defect-free hexagonal lattice inside a graphene grain. (**c**) Two grains (bottom left, top right) intersect with a 27° relative rotation. An aperiodic line of defects stitches the two grains together. (**d**) The image from c with the pentagons (blue), heptagons (red), and distorted hexagons (green) of the grain boundary outlined. (**b**–**d**) Images were low-pass-filtered to remove noise; scale bars, 5 Å. Reprinted with permission from Springer Nature© 2011 Macmillan Publishers Limited.

In 2011, Huang et al. [253] grew predominately single-layer graphene films on copper foils, and by using atomic-resolution imaging, determined the location and identity of every atom at a grain boundary (Figure 22), overcoming the problem of the five-order-of- magnitude size difference between grains and the atoms at grain boundaries.

It was found that the grain boundary is not straight, with not periodic defects along the boundary; different grains stitch together predominantly through pentagon–heptagon pairs.

In 2008, Blangert et al. [254] evidenced regions including one to a few layers of graphenes via HAADF images in graphene membranes, while in 2012, Zhou et al. [255] analyzed graphene grown by CVD on a Ni film on a silicon wafer, where Si is a common impurity. Using ADF and HAADF images, it was possible to track atoms diffusing in the graphene lattice, providing a full description of their local chemical environment.

### 3.2. Scanning Probe Microscopy

Imaging of solid surfaces at an atomic resolution is commonly achieved using Atomic Force Microscopy (AFM) and Scanning Tunneling Microscopy (STM), the first based on the strong interatomic forces between atoms of the surface and sharp probe tips at a very short distance, the second exploiting the tunneling effect and monitoring the related current as the conducting tip moves across the surface.

AFM pictures are collected in three different modes: contact, noncontact, and tapping. Amplitude modulation (AM) AFM, the so-called tapping mode, has been predominantly employed for graphene imaging under ambient conditions and measurement of the graphene sheets’ thickness on different substrates [256,257,258,259,260,261].

Pristine graphene thickness is reported to be around 0.34 nm, much smaller than that of graphene oxide (1–1.5 nm), which may depend on various functional groups or adsorbed molecules present on the surface [262].

**Figure 23:**
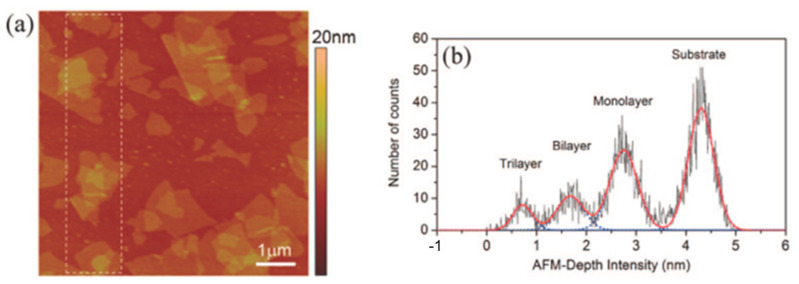
(**a**) AFM image of the GO films. (**b**) Histogram of the AFM-depth intensities obtained from the dotted area of the image (**a**). The histogram is fitted with a linear combination of four Gaussian functions representing each peak. Adapted with permission from [263]. Copyright {2009} American Chemical Society.

An interesting study on the structure of GO sheets deposited on a SiO_2_/Si substrate was carried out by Mkhoyan et al. [263] who could readily identify several GO sheets consisting of mono-, bi-, and trilayers in the AFM image (Figure 23); from the histograms of the AFM-depth profiles, quantitative analysis of flake height (or thickness) was conducted, showing that the thickness of a single sheet is about 1.6 nm and the ratio of thicknesses of the mono-, bi-, and trilayers scale is 1:1.6:2.2 (with actual values being 1.6:2.6:3.6 nm).

Further information on the lattice structure and surface morphology at atomic resolution can be obtained using STM. The possibility of carrying out STM measurements at low and high temperatures makes STM a powerful investigation method for thermal phenomena in real time [264,265].

**Figure 24:**
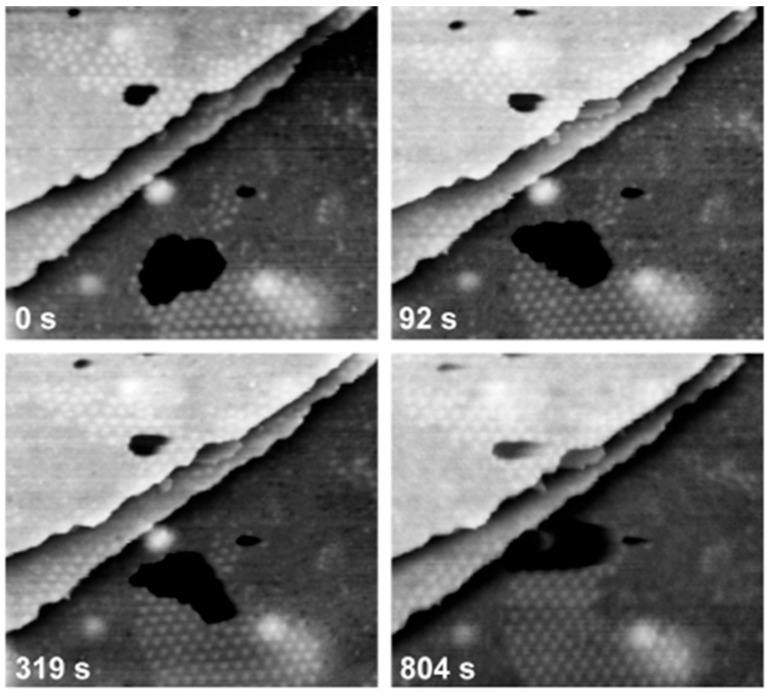
Series of STM images of the carbon-saturated layer taken during the phase transition of the disordered carbon layer into graphene at 920 K. The motion of the hole on the central terrace transforms the flat, disordered carbon layer into the graphene Moiré structure. 1100 Å × 1100 Å, tunneling current 0.07 nA, tunneling voltage 1.24 V. Reprinted with permission from [265]. Copyright {2013} American Chemical Society.

In 2013, Günther et al. [265] studied the ordering transition of an amorphous carbon layer into graphene at 920 K by monitoring it as a function of time using STM (Figure 24).

Time sequence shows that graphene is not formed homogeneously over the entire layer; a peculiar growth process involves motion in the carbon layer over unexpectedly large distances and in a seemingly directional, non-Brownian manner of topographic holes directly connected with the formation of the Moiré structure.

Another interesting investigation of the thermal evolution of individual graphene oxide sheets to graphene through high temperature (≥1773 K) heat treatment was carried out by Rozada et al. [266], who rationalized the process on the basis of the generation and dynamics of atomic vacancies in the carbon lattice.

**Figure 25:**
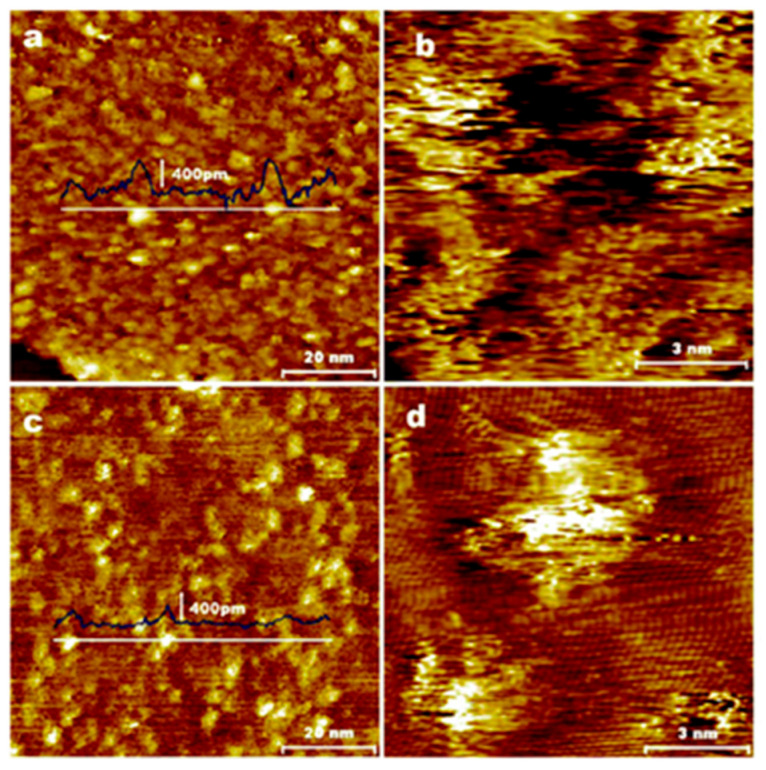

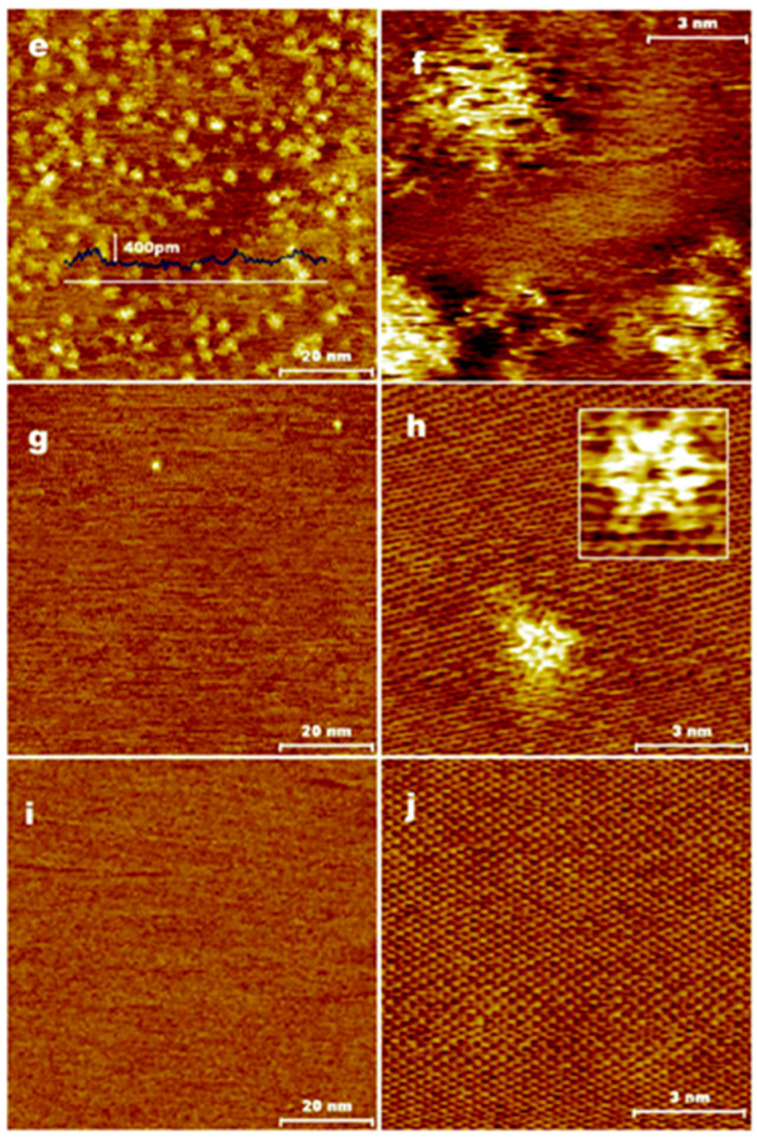
Nanometer- and atomic-scale STM images for the highly reduced graphene oxide sheets prior to thermal treatment (**a**,**b**) after annealing at 1773 K (**c**,**d**), 1923 K (**e**,**f**), 2073 K (**g**,**h**) and 2223 K (**i**,**j**). The inset to (**h**) is a detailed 2 × 2 nm^2^ image showing a different image of the same type of defect. Imaging conditions: 0.3 nA (tunneling current) and 500 mV (bias voltage) (**a**,**c**,**e**,**g**,**i**); 1–4 nA and 5–10 mV (**b**,**d**,**f**,**j**); 0.5 nA and 80 mV (h); 0.8 nA and 50 mV (inset). Adapted from [266] from the Royal Society of Chemistry. https://pubs.rsc.org/en/content/articlelanding/2015/nr/c4nr05816j (accessed on 1 March 2025).

It was evidenced via STM that a thorough conversion of graphene oxide/reduced graphene oxide into graphene sheets of high structural quality can be achieved via graphitization at temperatures as low as 2073 K if, prior to annealing, the amount of oxygen groups on the graphene oxide sheets is drastically decreased, e.g., by chemical reduction (Figure 25).

In fact, in this way, the thermal evolution of carbon atoms in CO/CO_2_ is almost negligible, and no atomic vacancies are created. Secondly, the substrate onto which the sheets must be supported must be inert, such as pristine atomically flat HOPG, in order to prevent interactions with the vacancies that could alter their migration dynamics.

### 3.3. Raman Spectroscopy

Raman Spectroscopy has found significant applications in the characterization of ordered and disordered crystal structures of carbon materials [267]; in particular, it can be efficiently used to monitor the number and quality of layers, bonding configuration of carbon atoms in graphene sheets, doping level, and confinement in graphene nanostructures. Graphene Raman spectra have some fingerprints that reflect changes in the electronic structure and electron–phonon interactions, allowing unambiguous, high-throughput, nondestructive identification of graphene layers [268,269].

**Figure 26:**
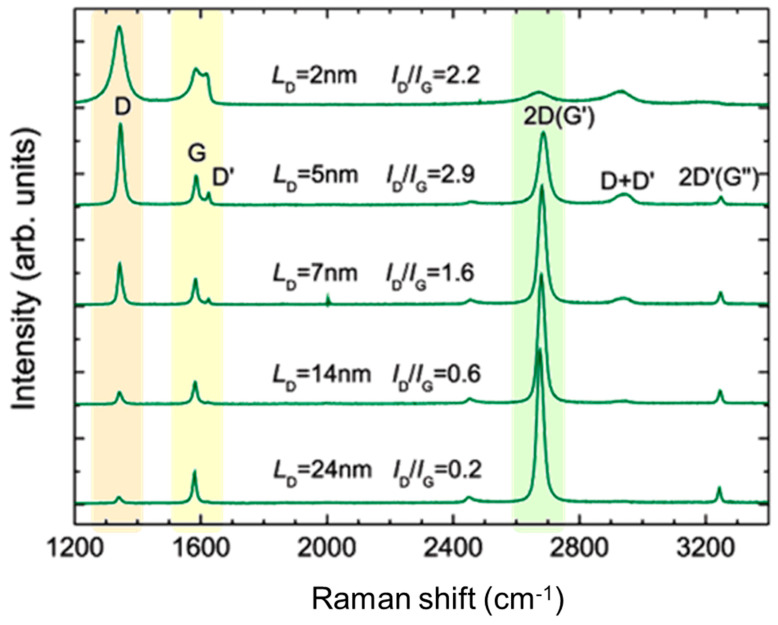
Raman spectra of five ion bombarded single layer graphene (SLG) measured at laser energy (E_L_) = 2.41 eV (λ_L_ = 514.5 nm), where the main Raman peaks are labeled. The distance between defects (L_D_) is independently measured following the procedure of Lucchese [271]. The respective I_D_/I_G_ values are indicated for each spectrum. The notations within parentheses [e.g., 2D(G’)] indicate two commonly used notations for the same peak (2D and G’) [267,269]. Adapted with permission from [270]. Copyright {2011} American Chemical Society.

The graphene Raman spectrum (Figure 26) has three characteristic peaks at 1580 cm^−1^, 1350 cm^−1,^ and 2700 cm^−1^ [270].

The first one is the G band at 1580 cm^−1^, associated with the first-order scattering of the E_2g_ mode arising from sp^2^ carbon atom domains.

The D band at 1350 cm^−1^ is associated with second-order Raman scattering process consisting of one-elastic and one-inelastic scatterings, and it is the so-called disorder-induced D-band. It is related to the vibrations of sp^3^ carbon atoms of disordered graphene nanosheets that usually appear in a disordered sample or at the edge of a graphene sample.

Raman data facilitate quantifying defects in graphene, which is a key step toward understanding the limits of its ultimate mobility. In graphene with zero-dimensional point-like defects, the distance between defects, L_D_, is a measure of the amount of disorder.

The third peak at 2700 cm^−1^ is the 2D band, also known as G’, and it is associated with a two-phonon double resonance Raman scattering process consisting of two inelastic scatterings. Cançado et al. [272] showed that the position and shape of the second-order 2D feature in the Raman spectra of graphene are sensitive to the number of layers of graphene sheets and can be used to evaluate the number of layers in a multilayer graphene sheet.

The first two Raman peaks, and in particular the D band, have been studied to obtain a deeper insight into the graphene quality.

**Figure 27:**
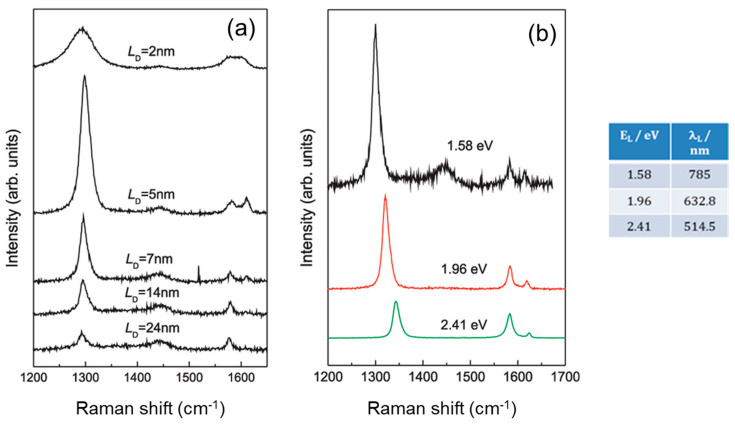
(**a**) Raman spectra of five ion-bombarded SLGs measured with laser excitation energy, E_L_, of 1.58 eV. (**b**) Raman spectra of an ion-bombarded SLG with L_D_ = 7 nm obtained using the three excitation energies reported in the table on the right. The ∼1450 cm^−1^ band in the Raman spectra at 785 nm is a third-order peak of the silicon substrate. Adapted with permission from [270] Copyright {2011}. American Chemical Society.

For this purpose, Cançado et al. [270] bombarded single-layer graphenes prepared on silicon with argon cations in a controlled and reproducible way, obtaining layers with various L_D,_ which were analyzed by means of Raman spectroscopy (Figure 27).

The Raman spectra of graphene change with the energy of the excitation source as well as the ratio between the D and G peak intensities, for a given defect density. The D band moves to higher Raman shifts, and I_D_ decreases as E_L_ increases (Figure 27b), which is a well-known effect in the Raman scattering of sp^2^ carbons.

In the graphene Raman spectra, analysis of the 2D peak is very significant since its shape, width, and position are affected by the number of graphene layers, reflecting the change in the electron bands via a double resonant Raman process [268].

**Figure 28:**
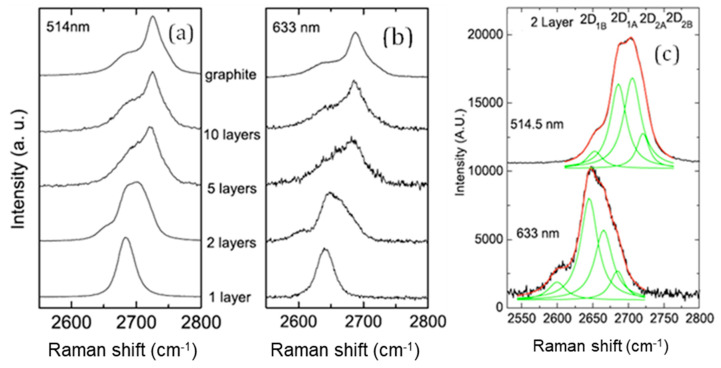
(**a**) Evolution of the spectra at 514 nm with the number of layers. (**b**) Evolution of the Raman spectra at 633 nm with the number of layers. (**c**) The four components of the 2D band in 2-layer graphene at 514 and 633 nm [268].

SLG is characterized by a single sharp 2D band (Figure 28a,b), and moving to a bilayer graphene, a much broader and up-shifted 2D band with respect to graphene appears. This can be fitted with four components, 2D_1B_, 2D_1A_, 2D_2A_, 2D_2B_ (Figure 28c), that can be associated with the splitting of π and π* electronic bands due to the interaction between layers.

The 2D band gets broader as the number of layers increases, until, for graphene with more than five layers, the Raman spectrum resembles that of the bulk graphite.

**Figure 29:**
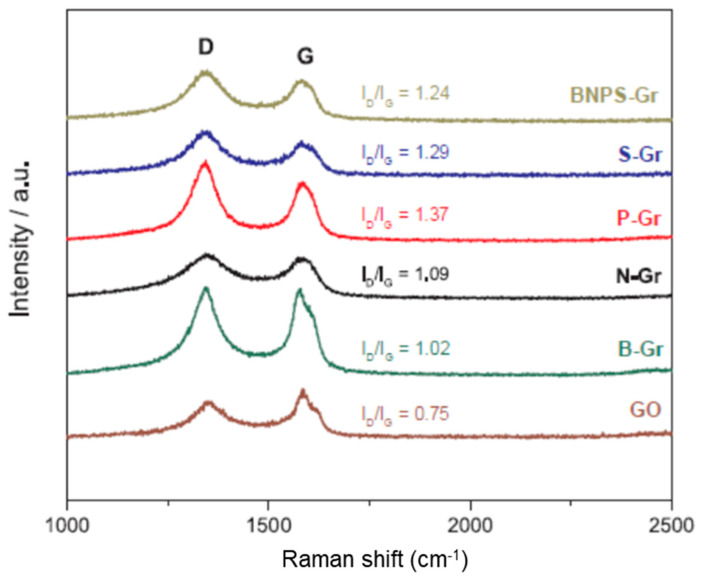
Raman spectra of the prepared catalysts were obtained using an excitation laser wavelength of 532 nm. The calculated I_D_/I_G_ values are also presented [273].

Raman spectroscopy is also useful in the characterization of heteroatom-doped graphene because the presence of the heteroatom is itself broadly a defect, with an increase in the I_D_/I_G_ ratio with respect to the undoped species. Molina-Garcia and Rees [273] analyzed single boron-doped (B-Gr), nitrogen-doped (N-Gr), phosphorus-doped (P-Gr), sulfur-doped (S-Gr), and quaternary-doped (BNPS-Gr) graphene (Figure 29).

The I_D_/I_G_ ratio of all the doped samples is higher than that of the starting material. Moreover, it is lower for B-Gr and N-Gr (ca. 1.05) with respect to P-Gr and S-Gr (ca. 1.35), suggesting that the integrity of the graphene structure is dependent on the size of the doping atoms, rather than the amount of doping species incorporated into the graphene layers. The quaternary-doped catalyst has the highest I_D_/I_G_ ratio (1.24), which reflects the incorporation of the different heteroatoms.

### 3.4. X-Ray Photoelectron Spectroscopy

XPS measurements allow the investigation of the surface composition and the oxidation states on the surface of graphene and its derivatives.

XPS survey spectra are collected to determine the surface elemental composition, while high-resolution XPS is used to determine the local bonding environments of C atoms.

In the case of graphene oxide and graphene oxide derivatives, this is accomplished by fitting the C_1s_ spectral envelope to include contributions from different surface oxides (e.g., C-O, C=O, O-C=O), all having peaks at different binding energies.

**Figure 30:**
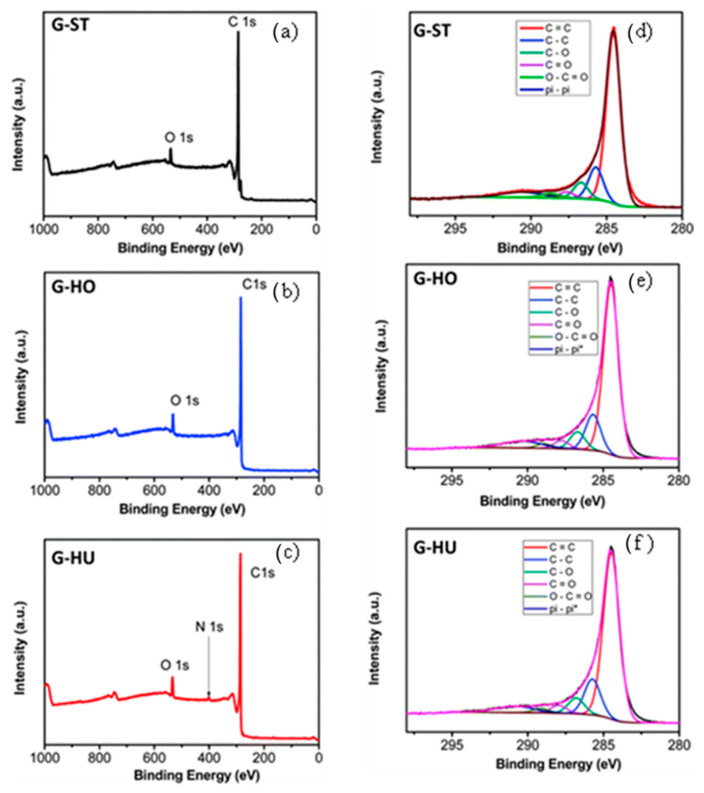
Wide-scan X-ray photoelectron spectra of (**a**) G-ST, (**b**) G-HO, and (**c**) G-HU, and high-resolution X-ray photoelectron spectra of the C_1s_ peak of (**d**) G-ST, (**e**) G-HO, and (**f**) G-HU. Adapted from [274] with permission from the Royal Society of Chemistry. https://pubs.rsc.org/en/content/articlelanding/2012/nr/c2nr30490b (accessed on 1 March 2025).

In 2012, Poh et al. [274] prepared graphene oxide via Staudenmaier, Hofmann, and Hummers methods, which underwent thermal treatment to be converted into graphene, named G-ST, G-HO, and G-HU, respectively. XPS evidenced, in particular, the presence of N in the G-HU (Figure 30c) and showed higher C/O ratios in the reduced species than in the parent species, confirming successful reduction.

Further insight into the types of oxygen-containing groups present in the three thermally reduced graphenes was given by high-resolution XPS spectra of C_1s_ for each sample. From careful fitting of the signal, five different carbon stages were quantitatively differentiated: the sp^2^-hybridized carbon atoms (284.5 eV), the sp^3^-hybridized carbon atoms (285.7 eV), the C–O of alcohol/ether groups (286.8 eV), the C=O of carbonyl groups (288.0 eV), and O–C=O of carboxyl acid/ester groups (289.2 eV). The π–π* shake-up signal (290.8 eV) was also found in all three thermally reduced graphenes, which is typical for sp^2^-hybridized carbon.

In the case of heteroatom-doped graphene, XPS is quite useful in distinguishing heteroatom configurations, which can be correlated, for example, with electrocatalytic properties.

**Figure 31:**
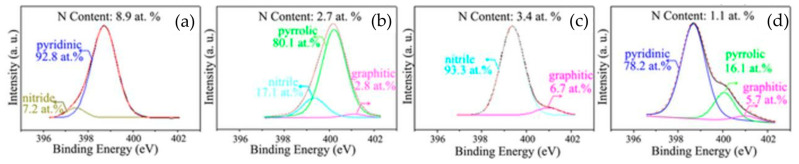
XPS profiles for samples (**a**) NG^−1^, (**b**) NG-2, (**c**) NG-3, and (**d**) NG-4. Reproduced with permission from IOP Publishing. From [275]. © 2017 The Electrochemical Society.

Hu et al. [275] collected XPS spectra of various N-doped samples (NG^−1^, NG-2, NG-3, NG-4); in particular, it was possible to detect various N configurations from the deconvolution of the N_1s_ peak (Figure 31) and the full width at half maximum (FWHM), the nitride- N (~397.5 eV, FWHM = 0.96 eV), pyridinic- N (~398.5 eV, FWHM = 1.29 eV), nitrile- N (~399.3 eV, FWHM = 1.24 eV), pyrrolic- N (~400.2 eV, FWHM = 1.24 eV), and graphitic- N (~401.1 eV, FWHM = 1.30 eV).

### 3.5. X-Ray Diffraction

XRD is a useful technique for analyzing crystalline nanomaterials and determining the crystal structure and size of nanoparticles on the basis of the Debye–Scherrer equation.

$$d=\frac{K\lambda }{\beta \cos\theta }\;$$

where K is the Scherrer constant; λ is the wavelength of light used for the diffraction; β is the “full-width at half maximum” of the sharp peaks and θ is the measured angle.

Using XRD, it is possible to distinguish and identify graphite, graphene oxide, and reduced graphene oxide. The XRD pattern of pristine graphite with a graphitic structure exhibits a strong and sharp peak at 2θ at approximately 26°, corresponding to an interlayer spacing of 0.334 nm [276]. The successful oxidation of graphite implies the loss of the typical graphitic structure in graphite because of the introduction of oxygen functionalities onto the carbon basal plane [277]. A new diffraction peak, the characteristic GO peak of the (002) plane, appears at 2θ within a range between 9 and 13° with different associated interlayer spacing. The variation in the interlayer spacing of GO is a result of variations in the degree of oxidation of the graphite, and according to Marcano et al. [69], it is proportional to the degree of oxidation.

XRD has also been used in the study of the reduction of GO to rGO carried out by Shin et al. [87], who treated graphite oxide with different amounts of NaBH_4_.

**Figure 32:**
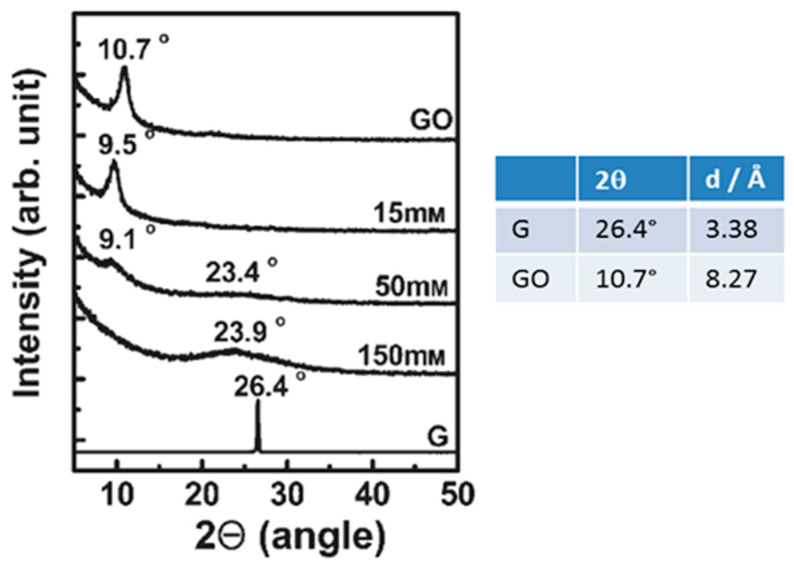
XRD patterns of GO, graphite (G), and reduced GOs obtained using various molar concentrations of NaBH_4_. A table with the interlayer distance is reported beside the graph [87] © 2009 WILEY-VCH Verlag GmbH & Co. KGaA, Weinheim.

In the XRD spectra (Figure 32), it is again evident that the increased interlayer distance between graphite and graphene oxide, attributed to the hydroxyl and epoxy groups between the carbon sheets introduced by the oxidation treatment, can lead to the decrease in van der Waals forces between the graphite sheets in the exfoliated GO.

Upon addition of NaBH_4,_ the interlayer distance is further increased up to 9.72 Å nm, but with 150 mM of NaBH_4_, the peak of the large interlayer distance disappears completely, and a broad peak near 0.3.73 Å (2θ = 23.98°) becomes visible, which suggests that most of the functional groups were removed.

**Figure 33:**
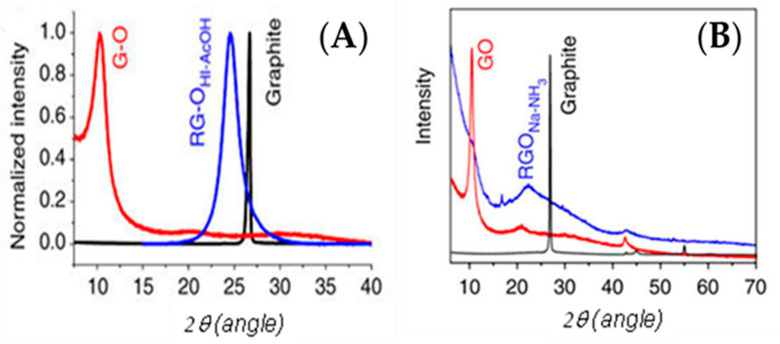
(**A**) XRD spectra of graphite, GO, and RGO_HI–AcOH_ [277]. Adapted with permission from Springer Nature Communications © 2010 Macmillan Publishers Limited. (**B**) XRD spectra of graphite, GO, and RGO_Na_-_NH3_ powder [279]. Adapted with permission from Springer Nature Communications © 2013 Macmillan Publishers Limited.

Other reduced graphene oxide obtained via reduction of GO with hydriodic and acetic acid (RGO_HI–AcOH_) [278] and with sodium–ammonia solution (RGO_Na_-_NH3_) [279] present XRD spectra with a distinguishable peak for the reduced GO at θ = 24.57° (interplanar distance = 3.62 Å) and at θ = 25.04° (interplanar distance = 3.56 Å) (Figure 33).

### 3.6. Ultraviolet–Visible Spectroscopy

**Figure 34:**
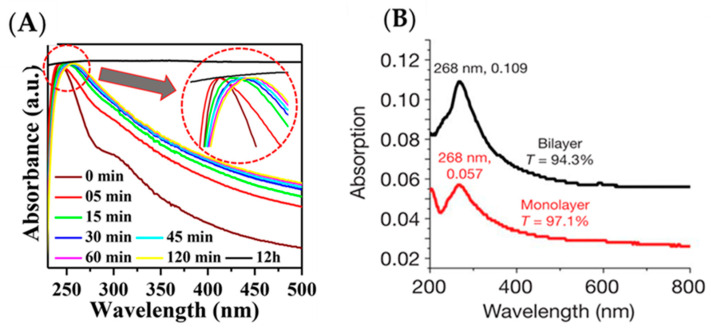
(**A**) Optical absorption spectrum of GO and rGO with different reduction times. Zoomed image of the optical absorption spectrum (inset) [280]. (**B**) The UV-Vis absorption spectra of monolayer graphene and bilayer graphene [280]; peaks are labelled with a wavelength of maximum absorption and the value of maximum absorption. The UV transmittance (T in %) is measured at 550 nm. Adapted with permission from Springer Nature Communications © 2010 Macmillan Publishers Limited.

UV-Vis spectroscopy is quite useful for identifying graphene and its oxide because of their different UV-Vis absorptions, as shown in Figure 34A, where the reduction of GO with hydrazine was monitored during the reaction [280]. By increasing the duration, a large red shift is observed because of the reduction of GO to rGO.

GO presents two characteristic absorption peaks, one intense at 230 nm related to π-π* transition of the C=C bond, and one weak at 305 nm corresponding to n-π* transition of the C=O bonds of GO [281,282]. A different UV graphene characteristic peak is observed at 268 nm.

Another important feature is that the electronic properties of graphene are strongly linked to its thickness, and the absorption depends linearly on the number of layers: the intensity of the band at 268 nm is almost doubled going from monolayer to bilayer graphene (Figure 34B) [281].

**Figure 35:**
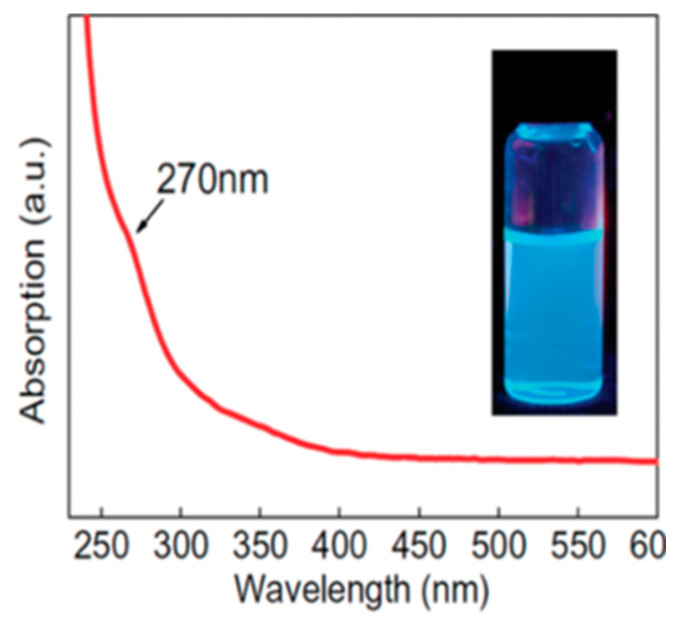
UV−Vis absorption of NG_qDot_; in the inset is the photo of the corresponding solution in water under 365 nm UV irradiation. Adapted with permission from [283]. Copyright {2012} American Chemical Society.

UV-Vis also shows evidence of heteroatom-doping, as in the case of N-doped quantum dots NG_qDot_: the absorption band at ca. 270 nm (Figure 35) [283] in the absorption spectrum is blue-shifted by ca. 50 nm with respect to that of N-free G_qDot_ of similar size [284].

### 3.7. X-Ray Absorption Near-Edge Structure

The X-ray absorption near-edge structure (XANES) spectroscopy, also referred to as Near-edge X-ray Absorption Fine Structure (NEXAFS) spectroscopy, is an element-specific spectroscopic technique for probing photoexcitation of electrons from a core level (absorption edge) of an atom to levels within an energy range of ∼50 eV above the absorption edge.

Therefore, being sensitive to the local chemistry of the atom under detection using edges as a “fingerprint” of the electronic structure offers the possibility of investigating the chemical bonding, electronic structure, and interactions of the materials with the advantage of not destroying the sample [285,286].

A NEXAFS study of single-layer graphene was carried out in 2008 by Pacilè et al. [287], who observed a splitting of the π* resonance and a clear signature of the predicted interlayer state. By comparing spectra of single-layer, bilayer, and few-layer graphene samples, it was possible to illustrate the rapid evolution of the electronic states from those of a truly two-dimensional (2D) system towards those of bulk graphite.

Since NEXAFS is particularly powerful at characterizing carbon-related materials by providing information on the degree of bond hybridization in mixed sp^2^/sp^3^-bonded carbon, the specific bonding configurations of foreign functional atoms, and the degree of alignment of graphitic crystal structures [287], it is particularly suitable for studying doped graphene [288,289,290,291].

**Figure 36:**
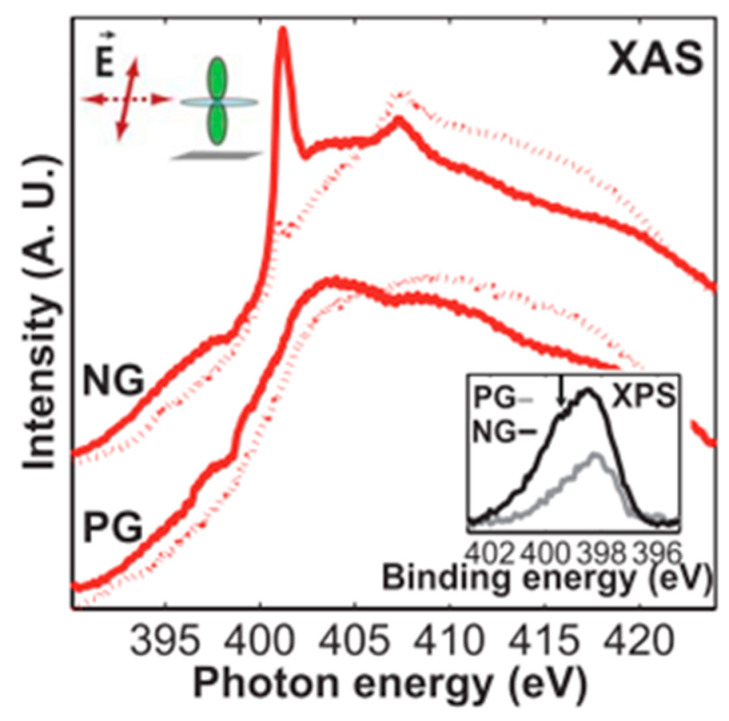
NEXAFS (total electron yield) of pristine graphene (PG) and N-doped graphene (NG) on copper foil at the N K-edge. (Inset) XPS data for pristine and N-doped graphene, showing a higher-binding energy component (black arrow) for the doped sample [178]. Copyright © 2011, The American Association for the Advancement of Science.

XANES measurements of pristine and N-doped graphene on copper foil at the nitrogen K-edge (Figure 36) carried out by Zhao et al. [178] confirmed the presence of N in the system. N-doping results in sharp peaks at 400.7 and 408 eV in the NEXAFS spectrum, associated with 1s-to-π* and -σ* transitions, respectively, for a single molecular species. The new peak in the spectrum at 400.7 eV is due to graphitic N.

**Figure 37:**
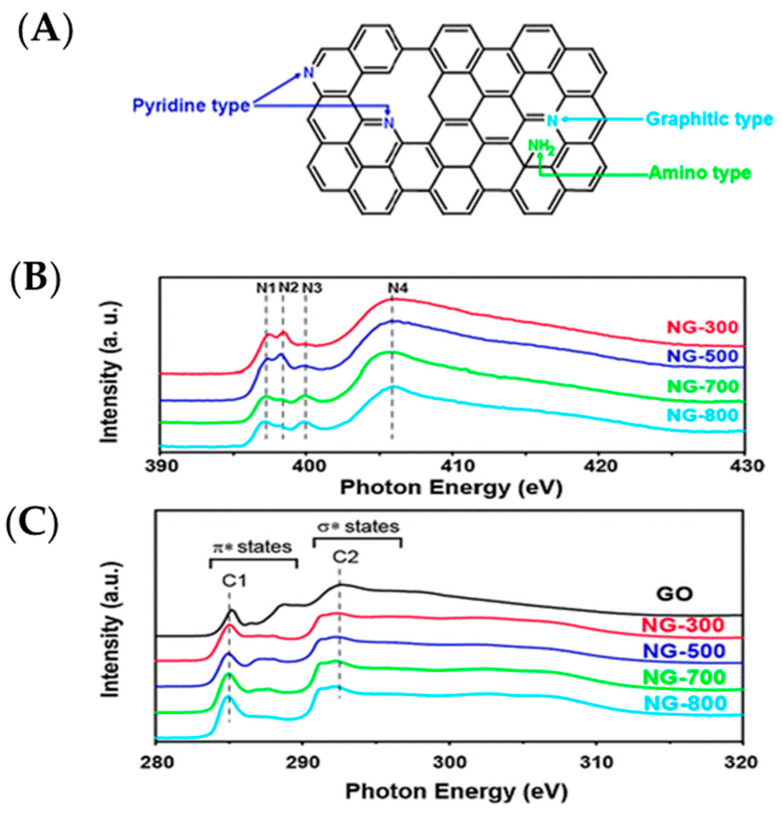
(**A**) Nitrogen species doped in the graphene layer. (**B**) N K-edge XANES spectra of NG-300, NG-500, NG-700, and NG-800. (**C**) C K-edge XANES spectra of NG-300, NG-500, NG-700, and NG-800. Adapted from [292] with permission from the Royal Society of Chemistry. https://pubs.rsc.org/en/content/articlelanding/2015/ta/c5ta01561h (accessed on 1 March 2025).

XANES measurements provided unambiguous evidence for the presence of more nitrogen species (pyridinic and amino group) in GO treated (Figure 37) at 300 °C (sample NG 300), 500 °C (sample NG 500), 700 °C (sample NG 700), and 800 °C (sample NG 800), with ammonia used as supporting materials to load Pt nanoparticles [292].

The XANES spectra show four well-resolved resonance peaks, N1, N2, N3, and N4, located at ca 397.4 eV, ca 398.2 eV, ca 399.8 eV, and ca 406 eV, respectively. In contrast, peak N4 is assigned to general transitions from the N 1s core level to C–N σ* states, peak N1 and N3 are attributed to the pyridinic (C=N π*) and graphitic type nitrogen species, respectively, and peak N2 is attributed to amino type species.

It is also interesting to observe the evolution of XANES peaks at C edge with the temperature; on raising the temperature, the intensity of C1 at around 285 eV due to C–C π* (ring) transitions increases, while the intensity of C2 at around 292.4 eV due to C–C σ* (ring) transitions decreases, indicating the increasing dominance of the π-network because of the removal of the oxygenated groups during the treatment.

A detailed analysis of the absorption edges of the dopant elements in doped and co-doped GO_qDot_ was carried out by Favaro et al. in 2015 [293]. In the case of S and S,N-GOqDot, the S L edge spectra show the presence of –SH (S1, 162.8 eV), C–S–C (S2, 164.2 eV), and –SO_3_ (S3, 166.8 eV) groups. The S,N-GO_qDot_ S L edge spectra are also characterized by a broad peak between 165 and 168 eV due to the concomitant presence of a relatively high amount of oxidized S-based groups and S–N groups whose 1s–π* transition is centered at 166.1 eV. Similarly, in the B K edge spectrum of B-GO_qDot,_ two distinct peaks are present, associated with the 1s–π* (at 189.3 eV) and 1s–σ* (at 191.5 eV) absorption transitions of the B–sp^2^ C and C–BO_2_ groups, respectively. In the case of N,B-GO_qDot_, C–BNO bonds are present as indicated by a further feature at 191.1 eV.

## 4. Nitrogen-Doped Graphene

Since Gong et al.’s work on vertically aligned nitrogen-containing carbon nanotubes [294] and following analogous investigation on N-doped graphene in 2010 [185] showing its superb ORR electrochemical properties in alkaline medium, intense research has been initiated to study the behavior of various N-doped graphenes as ORR electrocatalysts strictly correlated to the synthetic procedure, morphology, structure, and physicochemical properties of the material.

Nitrogen is considered one of the most effective dopants for enhancing the ORR activity of graphitic carbon materials [185,294,295,296]. The electron-accepting ability of the nitrogen atoms creates a net positive charge via intramolecular charge-transfer on adjacent carbon atoms in the nanocarbon structures to readily attract electrons from the anode to facilitate O_2_ adsorption and the ORR process [185,297].

Also, the nitrogen-induced charge delocalization changes the chemisorption mode of O_2_ from the usual end-on adsorption to a side-on adsorption, which effectively weakens the O–O bond, thus facilitating the oxygen reduction process [294]. Additionally, all intermediates involved in the oxygen reduction reaction bind to the carbon atom next to the nitrogen dopant [298].

The nitrogen doping introduces asymmetry in spin density and atomic charge density, making it possible for N-graphene to show high electrocatalytic activities for the ORR, as evidenced by the density functional theory [DFT] analysis of the ORR mechanism by Zhang and Xia [299]. Unlike pristine graphene, the nitrogen-doped graphene has a dramatically lower energy cost for the early catalytic ORR steps [300].

Nitrogen follows carbon in the periodic table, and by replacing C with N in the graphitic framework, the total number of electrons in the system can be tailored depending on the doping [301]. Since N has an atomic radius similar to C, significant lattice mismatches are prevented when N is introduced. N-doping can effectively improve the surface energy and reactivity of graphitic carbon frameworks with minimal damage to their properties [302].

**Figure 38:**
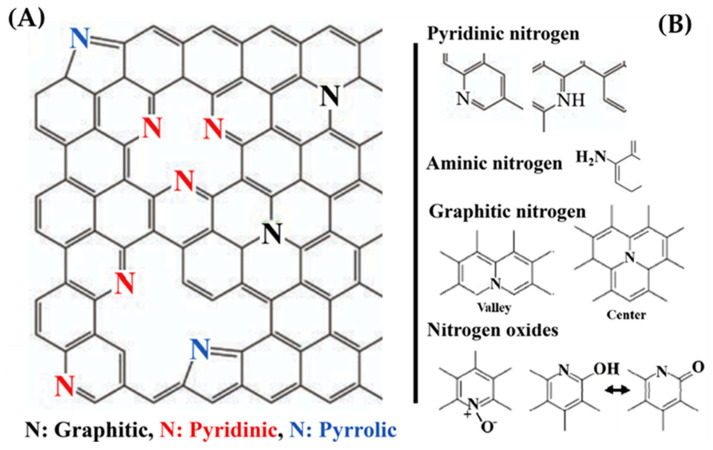
Various configurations of N atoms in a nitrogen-doped graphene layer. (**A**) graphitic-N, pyridinic-N, and pyrrolic-N are identified with different colors. (**B**) A detailed description of various N atom configurations from [303]. © 2018 Elsevier Ltd. (2018).

The introduction of N atoms into the matrix of sp^2^-bonded graphitic frameworks can lead to different configurations of nitrogen states: pyridinic N, pyrrolic N, graphitic [or quaternary] N, N-oxides, and amino group (Figure 38) [303].

Pyridinic N is attached to two carbon atoms in six-membered rings, possesses a lone pair of electrons, and donates one p electron to the π system; also, configurations with hydrogen can be present, as shown in Figure 38B. The pyrrolic N substitutes a carbon atom in five-membered rings and donates two p electrons to the π system. Both types of N occur at the edge of the layer but may also occur inside and are associated with vacancies.

Graphitic N refers to a N atom that bonds to three carbon atoms in graphene basal plane (graphitic/quaternary-N center) or occupies graphene zigzag edge or valley sites (graphitic-N valley), Figure 38B, and it is less impressionable to the protonation reaction because of an unavailable lone pair of electrons around N atom in the carbon plane [304].

In addition, nitrogen can be included in the form of nitrogen oxides or bound to the basal plane as an amino group Figure 38B. The detection and identification of the N configuration can be achieved using XPS via analysis of the nitrogen core level peak N_1s_ and its deconvolution.

There is extensive debate on the contribution of the nitrogen configuration (pyridinic, graphitic, pyrrolic, and amino) to ORR activity: the identity and role of the electrocatalytically active center are still controversial, with different nitrogen configurations often used to justify ORR activity and/or different ORR activity among correlated materials.

Beyond the intrinsic nature of the active sites, which is governed by the chemical compositions and bonding configurations of the doped atoms, there are two more factors to be considered that affect the ORR performance of heteroatom-doped nanostructured carbon materials: (1) the number of the available catalytic centers and the transport properties of reactants and electrolyte, which depend largely on the specific surface area and porous architecture; and (2) the electrical conductivity, which is mainly determined by the defects of carbon materials.

Because of the very wide scenario of cases and related studies, this review presents them using, as the first criterion, the claimed nitrogen configuration boosting the oxygen reduction kinetics.

In 2010, Long et al. [121] prepared N-doped graphene sheets via reduction of graphene oxide with hydrazine, first ascribing the high ORR activity in acidic media in part to the large amount of N pyridinic sites, as supported by previous work [305,306,307].

Many other studies followed, evidencing the relevant contribution of pyridinic N [127,275,304,308,309,310,311,312,313,314,315,316,317,318,319,320,321,322,323,324,325,326,327,328,329,330,331,332,333,334,335,336,337,338,339,340,341,342,343,344,345,346,347,348,349,350,351,352,353,354,355,356].

In 2014, Xing et al. [313] carried out an interesting study to determine the catalytic sites of three nitrogen-doped multilayer graphenes prepared from graphene oxide using different nitrogen sources and doping methods for different nitrogen doping configurations. The chemical composition changes before and after ORR were examined via synchrotron-based X-ray photoelectron spectroscopy analyses of chemisorbed oxygen reduction intermediates.

On the basis of the commonly accepted four-electron associative ORR mechanism of N-doped graphene in alkaline solution, intermediate OH_ads_ is formed from O_ads_ with the addition of H_2_O and one electron: the oxygen reduction intermediate OH_ads_, which should chemically attach to the active sites, was found to remain on the carbon atoms neighboring pyridinic nitrogen after ORR, indicating that the carbon atoms close to pyridinic nitrogen are the main active sites in the different nitrogen doping configurations. In addition, a high amount of OH_ads_ attachment after ORR corresponds to a high catalytic efficiency and vice versa.

Synchrotron-based X-ray photoelectron spectroscopy analyses of three nitrogen-doped multilayer graphene samples revealed that the oxygen reduction intermediate OH_ads_, which should chemically attach to the active sites, remains on the carbon atoms neighboring pyridinic nitrogen after ORR. Moreover, the location or microstructure of pyridinic nitrogen can affect its catalytic efficiency.

In 2016, Farzaneh et al. [317] hydrothermally synthesized three-dimensional (3D) mesoporous nitrogen-doped reduced graphene oxides [N-rGO_3Dmpo_] with different nitrogen contents [1.0–4.7%] containing different N-pyridinic amounts and carried out research on the role of π and lone pair electrons. Pyridinic-N contributes one π electron to the aromatic π system and possesses an electron pair in the plane of the carbon matrix. This electron pair increases the electron–donor property of the catalyst, weakening the O-O bond via bonding between oxygen and nitrogen and/or an adjacent carbon atom, and facilitating the reduction of O_2_. Pyridinic N at the edge of the carbon plane is also more exposed and thus can be more available for O_2_ molecules [305,356,357].

It was shown for the first time that there are four different regimes governing ORR on N-doped graphenes depending on the applied potential: at lower potentials, N-content of N-rGO_3Dmpo_ [regardless of their bonding configurations] promotes ORR performance due to activation and strengthening of π-electrons of graphene framework, while pyridinic-N content has a significant role in ORR at higher potentials because of activation of their lone pair electrons. Furthermore, N-rGO_3Dmpo,_ containing 4.1% nitrogen, was tested as a cathode electrocatalyst in the direct methanol fuel cells, which produced superior output power compared to commercial 20 wt% Pt/C.

**Scheme 4:**
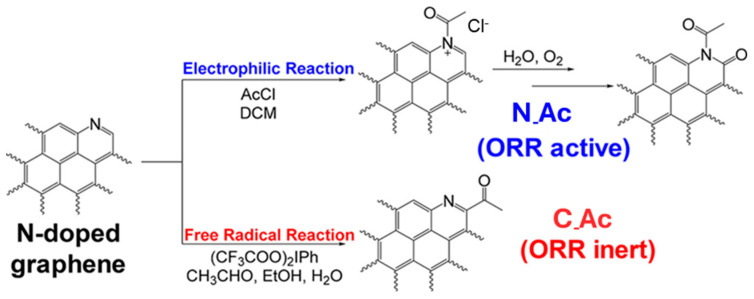
Selective modification of the acetyl group on the N atom and ortho-C atom of the pyridinic ring for a N-doped graphene catalyst, inspired by selective substitution for the quinoline derivative in organic synthesis. AcCl = acetylchloride, DCM = dichloromethane. Reprinted with permission from [334]. Copyright {2018} American Chemical Society.

In 2018, Wang et al. [334] developed a surface modification method to identify the ortho-carbon atom of the pyridinic ring as the reactive site for ORR on N-doped graphene, taking advantage of the molecular probe approach. They first prepared N-doped graphene containing higher amounts of pyridinic N and carried out a targeted modification: the pyridinic ring on N-doped graphene was highly selectively grafted by the acetyl group Ac at the pyridinic N atom, denoted as N_Ac, via an electrophilic reaction and ortho-C atom denoted as C_Ac through electrophilic and via a free radical reaction (Scheme 4).

It was observed that attaching a functional group to the pyridinic N atom of N-doped graphene did not affect the ORR activity, which was mostly preserved, while it was completely suppressed when the ortho-carbon of the pyridinic ring was blocked. DFT calculations revealed that the distinct ORR activity between N_Ac and C_Ac mainly comes from the difference in positive charge density and spin density at the ortho-C atom.

Therefore, it was concluded that the active species of N-doped carbon for ORR in acidic solution is the ortho-C atom of the pyridinic ring, on which O_2_ can be absorbed and reduced favorably.

It is worth mentioning the efforts that have been made to develop facile methods for the synthesis of pyridinic-N-doped graphene, trying to avoid high temperatures and using simple solution chemistry [333,335,349]. In 2019, Liu et al. [349] converted holey graphene into nitrogen-doped graphene by chemically grafting diamino-benzene derivatives to the ortho-quinone sites on the abundant holey graphene edges through a simple condensation reaction. The ORR performance depends on the crafted N types. The 3,4-diaminopyridine grafted holey graphene [A3-HG] containing pyridinic N possesses the best ORR performance, showing excellent ORR activity comparable to Pt/C in alkaline media, as well as good selectivity and stability (Table 2). The Zn–air batteries assembled with A3-HG electrocatalyst outperformed those assembled using commercial Pt/C electrocatalyst, achieving a high energy density of 495 Whkg^−1^, much higher than that of the Pt/C [198 Whkg^−1^].

Another interesting method for controlling pyridinic N in graphene and preventing the formation of other nitrogen species was offered by Zhong et al. [358], who carried out pyridine cycloaddition of graphene, obtaining a graphene derivative with an activity comparable to that of the commercial Pt/C electrocatalyst because of the synergistic effect of pyridine and graphene. Such a derivative is characterized by pyridine groups “external” and covalently attached to graphene, being in a vertical direction with respect to the basal plane. DFT calculations revealed that the ortho-carbon of the “external” nitrogen could be a possible adsorption and catalytic site.

In addition to the rich literature evidencing the role of pyridinic nitrogen related to the modification of the band structure of carbon, raising the density of states near the Fermi level, and lowering the work function [205], many other papers illustrate, on the other hand, the contribution of graphitic nitrogen in the ORR electrochemical process. This is based on the fact that the relative electronegativity of graphitic N atoms reduces the electron density on the adjacent C nuclei, which helps electrons to transfer from the adjacent C to N atoms, and N back-donates electrons to adjacent C p_z_ orbitals [205]. The donation and back-donation processes not only facilitate O_2_ dissociation on the adjacent C atoms but also help form a strong chemical bond between O and C. Graphitic-N is considered to provide active sites for ORR because its two p_z_ electrons can partially occupy the π* anti-bonding orbital around nitrogen [359]. A theoretical study by Nandhini et al. [360] on the identification of site-dependent activity in alkaline media evidenced a strong correlation between the activity of each C site with the occupancy of p_z_[π] electrons of the corresponding site. OH adsorption is found to have a linear relationship with p_z_ occupancy; the occupancy of p_z_[π] electrons of C atoms increased near the dopant site due to the back-donation mechanism. Carbon atoms in the vicinity of the N atom are enriched with different p_z_[π] electron densities due to the back-donation mechanism. Hence, unequal binding strength is observed for each carbon atom of N-doped graphene, and the C atoms that are far from the N dopant site show similar behavior to the C atoms in the pure graphene surface.

Additional theoretical investigations were carried out to analyze the impact of different nitrogen doping on oxygen adsorption and on the energy barrier of oxygen molecule dissociation [299,361,362,363,364,365,366] and to explore in more detail the chemical reactivity, reaction pathway, electronic transport characteristics, and experimental conditions, such as the use of water as a solvent and the pH [360,367,368,369,370]. Additionally, particular structures, such as quantum dots and nanoribbons, have also been studied [371,372,373,374,375,376].

Scardamaglia et al. [377] used first-principle calculations to support the modification of the N_1s_ line-shape observed by in situ high-resolution synchrotron techniques in the interaction of molecular oxygen with graphene doped via nitrogen plasma. Upon exposing N-graphene/Ir samples in situ to 5 × 10^−5^ mbar of O_2_ at a temperature of 200 °C for 30 min, oxygen dissociation and formation of carbon–oxygen single bonds occurred on graphene, along with a band gap opening and a rounding of the Dirac cone. The modification of the N 1s line-shape reveals, in particular, a change in the chemical environment of graphitic nitrogen upon oxidation that is consistent with the adsorption of two oxygen atoms in the epoxy position on the nearest carbon atom neighbors of the graphitic nitrogen, as predicted theoretically. The graphitic nitrogen is involved in the observed mechanism: the adsorbed oxygen molecule is dissociated, and the two O atoms chemisorb via epoxy bonds to the nearest carbon neighbors of the graphitic nitrogen. This is the first direct experimental description of the first step in the oxygen reduction reaction, providing a detailed atomic-level understanding of the role of the nitrogen active sites in the interaction with molecular oxygen.

Besides theoretical and spectroscopic investigations, many experimental studies have proven the effectiveness of graphitic N in promoting the ORR [378,379,380,381,382,383,384,385,386,387,388,389,390,391,392,393,394,395,396,397,398,399,400,401,402,403,404,405,406,407,408,409,410,411]. Geng et al. [378] were the first to prepare nitrogen-doped graphene by heating graphene in the presence of ammonia at different temperatures. The sample treated at 900 °C had the best high ORR activity through a four-electron transfer process in alkaline media, comparable to Pt/C (Table 2). By comparison with the samples heated at 800 °C and 1000 °C, which contain lower relative amounts of quaternary nitrogen than the sample heated at 900 °C, they evidenced that quaternary nitrogen atoms could be considered the most important species for the ORR due to the relationship between activity and quaternary N contents.

An interesting study of the dependence of catalytic activity on the nitrogen configurations was carried out by Lin et al. [384], who prepared N-doped graphene via pyrolysis of graphene oxide/polypyrrole composite. Starting from the N pyrrolic configuration [100%] of the initial composite, by heating above 300 °C, the pyrrolic N percentage decreased, while the pyridinic N percentage increased monotonically, with transformation from pyrrolic N to pyridinic N. However, at T > 600 °C, the pyridinic N percentage started to drop with a simultaneous increase in graphitic N percentage, implying the transformation of pyridinic N to graphitic N. Moreover, the direct transformation of pyrrolic N to graphitic N may occur at temperatures above 500 °C. The sample heated at 900 °C, containing the highest N graphitic percentage among all the samples, gave the best ORR performance in alkaline media.

Analogous thermal behavior of nitrogen configurations was driven and observed by Lu et al. [403] in N-doped graphenes prepared via pyrolysis of graphene oxide. First, they observed that the ORR activity is not proportional to the total nitrogen content, which decreases as the pyrolysis temperature increases up to 1000 °C; an increase in the annealing temperature can enhance the content of graphitic N, and the enriched graphitic sample obtained at 1000 °C had the best catalytic ORR activity and outperformed Pt/C (Table 2). Moreover, it showed appreciable catalytic activity also at acidic pH (Table 3).

DFT calculations evidenced that oxygen can be stably adsorbed on the C site neighboring the N atoms, while H_2_O can be weakly adsorbed and therefore easily released as an ORR product. More recently, another study by Guo et al. [406] on graphitic-N-rich N-doped graphene was conducted to investigate the effective mechanism of the graphitic nitrogen on the ORR activity of graphene and to better understand the relationship between the N dopant species and catalytic activity, indicating the interest in addressing the synthesis of N-doped graphenes towards tailored N configurations. DFT calculations were first performed; on the basis of electrostatic potential analysesit was showed that the oxygen molecule cannot bind to the pyridinic N site. Secondly, on the basis of the average local ionization energy on the molecular surface, widely used to evaluate electron reactivity, the minimum point can be observed near the graphitic N, while no minimum point can be found near the pyrrolic N [411,412]. Even if both pyrrolic N and graphitic N can bind with oxygen, the electron around graphitic N has large reactivity, so that the reaction occurs on the graphitic N site.

The general synthetic method to get enriched N graphitic graphene is based on pyrolysis operating at high temperature; microwave irradiation was alternatively used [389,401]. It is a simple, facile, and efficient heating process [413,414,415] with several advantages, including fast, volumetric, and uniform heat transfer to the reactants, thereby causing a homogeneous synthesis condition at elevated temperatures.

Wang et al. [389] produced N-doped graphene with different characteristics in a very short time. Through microwave heating, an efficient reductive process occurs, with the O content of graphene reduced to around 5 wt% from 19.2 wt%. The N content increased from 0 to over 5 wt%. The N-doping process via microwave heating required only a few seconds (2–30 s), which is much less time than thermal annealing (30 min) [380], and a considerable advantage compared with previous methods.

Two more N-configurations to be mentioned are the N-pyrrolic and N-amino configurations. In 2011, Zhang and Xia [299] studied the electrocatalytic mechanism of N-doped graphene in an acidic environment using DFT. Two different nitrogen-containing graphene sheets [C_45_NH_20_ and C_45_NH_18_] were built, containing pyridine and pyrrole species for which different active catalytic sites were identified, having either high positive spin density or high positive atomic charge density. Because of asymmetric spin density and atomic charge density, the N-doped graphenes containing pyridinic or pyrrolic nitrogen are predicted to show appreciable electrocatalytic activities in the ORR.

Unni et al. [416] were the first to ascribe the enhanced catalytic activity towards ORR of the prepared N-doped samples obtained from GO and pyrrole used as nitrogen dopant and reducing agent to the enhanced proportions of the pyrrolic nitrogen along with the mesoporous structure of graphene. Then, Liu et al. [417] proposed, reasonably, that the pyrrolic-N structure is the main contributor to the four-electron transfer mechanism of the ORR of N-rGO samples prepared under microwave irradiation. More recently, the amount of N-pyrrolic has been considered fundamental in driving the selectivity of N-rGO toward the formation of H_2_O_2_, involving only two electrons [418,419,420,421]. Li et al. [418] showed that the sample with the highest pyrrolic-N content and abundant in-plane pores had superior activity toward electrochemical H_2_O_2_ synthesis, with a selectivity as high as 95% and excellent long-term stability. In particular, the critical role of the pyrrolic-N was elucidated by the variable adsorption profiles of OOH* and O* intermediates on C K edge XANES spectra, as well as the dependent negative shifts of the pyrrolic-N peak on N K edge XANES spectra.

Regarding the amino group its role in the ORR graphene-based electrocatalyst is associated with its electron-donating ability: the functionalization of graphene with amine is supposed to be a promising strategy for improving the electrical conductivity of graphene due to the electron-donating effect of amino groups [422,423,424,425,426]. In particular, amine-functionalized holey graphene [424], prepared via hydrothermal reduction of graphene oxide in the presence of ammonia, followed by etching in the presence of KOH, is shown to have high electrocatalytic activity toward ORR, which is more kinetically facile than when the commercial Johnson Matthey Pt/C 40 wt% is used. This is explained by the fact that the presence of the electron-donating group and a large number of holes in its sheet plate can improve its electrical conductivity and give more active edge N atoms, thereby increasing the electrocatalytic activity of amine-functionalized holey graphene.

Considering functionalization more specifically as a way for molecular doping of graphene [427] in a few papers, other nitrogen-based groups, such as ammonium [428], imino [429], nitro [427], heterocycle [430], and amide groups [246,425,431] have been introduced. The basic concept is that such species can effectively modulate the electron density of graphene and therefore affect the electrochemical properties without altering the graphene basal plane.

There are many other studies where the ORR electrocatalytic properties are favored by the coexistence of various N configurations [432,433,434,435,436,437,438,439,440,441,442,443], in particular, by N-pyrrolic with N-pyridinic configuration [120,409,444,445,446,447,448,449,450,451,452] and by N-graphitic with N-pyridinic configuration, which is the most widespread [176,452,453,454,455,456,457,458,459,460,461,462,463,464,465,466,467,468,469,470,471,472,473,474,475,476,477,478,479,480,481,482,483,484,485,486,487].

**Figure 39:**
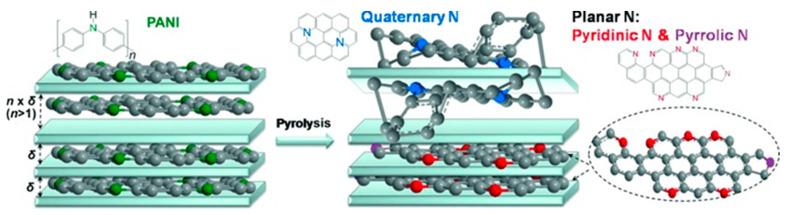
Schematic representation of NG synthesis showing the selectivity inside and outside of MMT during NG synthesis [452] © 2013 Wiley-VCH Verlag GmbH & Co. KGaA, Weinheim.

In the specific case of the combination of N-pyrrolic and N-pyridinic configurations, Ding et al. [452] developed a particular synthesis method based on the polymerization of aniline and pyrolysis carried out inside layered montmorillonite (MMT), which is used as a quasi-closed flat nanoreactor, since it is open only along the perimeter to enable the entrance of aniline monomer molecules. The flat MMT nanoreactor, less than 1 nm, facilitates the formation of pyridinic and pyrrolic N, characterized by planar sp^2^ hybridization (Figure 39), while it extensively constrains the formation of quaternary N because of its 3D structure. In this way, N-doped graphene (NG) containing selective N planar configurations could be obtained. The confinement effect of MMT ensures that N is incorporated into the structure and that the graphitization is successful without significant loss of N species.

The ORR activities of the prepared NG decreased as the planar N percentage and the electrical conductivity decreased; the sample with 90.3% N planar showed good catalytic activity toward the ORR in acidic media (Table 3), with a difference in the half-wave potential of 60 mV relative to Pt/C, high selectivity toward the ORR, stability, and a remarkable tolerance for methanol.

On dealing with the combination of graphitic and pyridinic nitrogen, it is interesting to report a study by Lai et al. [453] where the different roles of the pyridinic and graphitic nitrogen configurations were evidenced. Different N-doped graphenes were obtained by starting from different reagents: annealing of GO with ammonia preferentially formed graphitic N and pyridinic N centers, while annealing of polyaniline/rGO and polypyrrole/rGO tended to generate pyridinic and pyrrolic N moieties, respectively. Such graphenes were characterized by different ORR performances; while it was recognized that the dependence of electrocatalytic activity on the graphitic N content affected the limiting current density, the pyridinic N content improved the onset potential for ORR and might convert the ORR reaction mechanism from a 2e-dominated process to a 4e-dominated process. However, the ORR process is not affected by the total N content in the N-doped graphenes.

Analogous observations on the role of graphitic and pyridine N were made by Su et al. [488].

There are also computational studies aimed at evaluating the interplay between graphitic and pyridinic N in the ORR process [474,475]. Yan et al. [475] evidenced the synergic effect of graphitic and pyridinic N on the improvement of the adsorption and activation of O_2_. Combining graphitic and pyridinic N can induce further redistribution of charge among the carbon materials, which subsequently promotes O_2_ adsorption and lowers the energy barrier of O_2_ hydrogenation, demonstrating an improved property for ORR; hence, the synergic effect of graphitic and pyridinic N can strengthen the reduction performance of O_2_ by reducing the activation energy barrier.

As reported at the beginning of the paragraph, there are other elements beyond the particular N configuration that affect the ORR activity of the graphene-based catalysts, for example, the presence of exposed edges and defects, the conductivity, the morphology, and the microstructure of N-doped graphenes.

**Figure 40:**
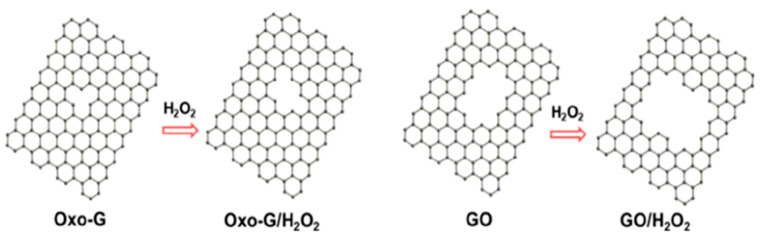
Scheme of the preparation procedure of GO precursors with different densities of in-plane lattice defects. Reprinted with permission from [499]. Copyright {2019} American Chemical Society.

Defective graphenes containing nitrogen as dopant with an optimized defect site density are generally characterized by enhanced ORR catalytic activity thanks to the synergistic effect of doping and defects/exposed edges [318,322,337,339,340,351,353,439,440,446,448,489,490,491,492,493,494,495,496,497,498,499,500,501,502,503], while an excess of defects can have detrimental effects on the ORR electrocatalytic behavior because of reduced conductivity [499,504]. Han et al. [499] investigated the influence of in-plane carbon lattice defects on the catalytic activity and selectivity toward ORR, and were able to tune the density of defects, identifying the best and worst conditions for significant ORR activity and selectivity to H_2_O_2_ formation. Different graphene and graphene oxide precursors (oxo-G, obtained from mild oxidation of graphite, and GO, obtained from Hummers’ method) underwent an oxidative-etching process by mixing with H_2_O_2_ under hydrothermal conditions (Figure 40).

In this way, they could obtain four graphene precursors with different defect densities, which were afterwards hydrothermally treated with ammonium hydroxide to achieve nitrogen doping. Hetero-doping was revealed to improve the ORR performance and the presence of a moderate defect density, as the sample obtained from oxo-G induced selectivity to H_2_O_2_ production and the highest electron density below 0.5 V. The sample with higher N content, higher defect density, and higher electrochemically active surface area was obtained from the precursor with GO treated with H_2_O_2_, but did not show the highest ORR activity and H_2_O_2_ selectivity, which may be due to the fact that an excess of in-plane carbon lattice defects lead to reduced conductivity.

Defect engineering is very important for generating active sites [375], and to avoid disrupting the honeycomb lattice of the graphene basal plane, the scientists have tended to study and work on the edge positions to maximize the number of exposed edge sites [preferably zigzag] relative to catalytically inert basal plane sites [325,348,352,496,505,506,507,508,509].

This can be achieved, in particular, in graphene quantum dots (G_qDot_): the spherical shape and reduced size of graphene quantum dots offer a greater proportion of edge sites compared to graphene [510]. N-doped graphene quantum dots have shown improved ORR electrocatalytic properties [283,316,319,329,391,410,511,512,513] (Table 3) and have been employed as defective surface modification additives to generate defect-rich graphene/carbon quantum dot composites.

The integration of a defect-rich G_qDot_ in the graphene matrix was achieved hydrothermally by Wang et al. [513]. The product was mixed with melamine and heated at different temperatures to allow N doping by melamine and activate fabrication defects. Thanks to a great density of defects and N doping, the sample obtained at 1000 °C has an excellent electrocatalytic activity for the oxygen reduction reaction, with a nearly four-electron pathway. The application potential of such material was also evaluated. An aqueous zinc/air battery was fabricated by employing it as a cathode catalyst, obtaining a much higher discharge voltage at the current density range from 20 to 100 mAcm^−2^ compared to Pt/C, leading to a higher power density for zinc/air batteries. The stability during the discharge process is also better than that of Pt/C.

**Figure 41:**
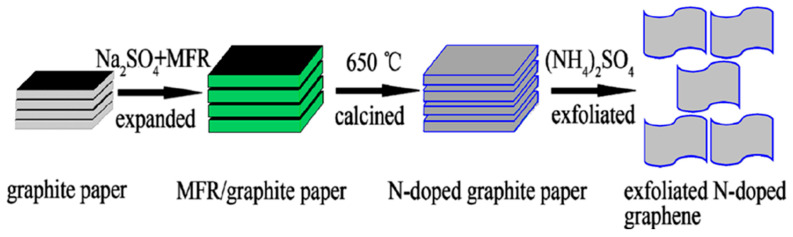
Schematic illustration of the fabrication process of electrochemically exfoliated N-doped graphene. Reprinted from [515] with permission from Springer Journal of Solid State Electrochemistry © Springer-Verlag Berlin Heidelberg 2016.

The preservation and enhancement of the conductivity of the sp^2^ carbon network combined with N-doping is a well-defined issue in graphene-based ORR electrocatalysis [304,332,375,380,497,514,515,516,517]. Chao et al. [515] developed a facile solution-processed strategy to fabricate N-doped graphene consisting of two-step electrochemical exfoliation: in the first step of exfoliation, graphite paper is expanded in a weak exfoliation electrolyte, Na_2_SO_4_ aqueous solution, permeated with melamine–formaldehyde resin monomer (MFR), and calcined (Figure 41).

The obtained N-doped graphite was further completely exfoliated in (NH_4_)_2_SO_4_ electrolyte, taking advantage of the much larger number of gas species, i.e., NH_3_, which can be produced, resulting in a stronger force for exfoliating N-doped graphite paper into thin-layer graphene. The resultant N-doped graphene contains a high level of nitrogen (7.9 at%) and presents outstanding electrical conductivity despite abundant oxygen species, which, however, impart good wettability, facilitating effective ionic transport.

An effective way to ensure efficient mass transfer to the active sites and markedly facilitate the ORR diffusion kinetics is to construct graphene networks with convenient and abundant porosity characterized by a high surface area [518,519,520], which makes several active sites exposed and accessible to the electrolyte [352].

In order to increase the ORR active site density, the enlargement of the volumetric surface of the graphene catalysts [518] is used to achieve porous structures [336,355,464,490,521,522,523,524]. The 2D graphene sheets are converted to hollow spheres [409,444,479,525,526], nanocapsules [448], nanoring-integrated boxes [342], 3D graphene structures with various porosities [342,497], 3D interconnected nanostructures [432], aerogels [106,339,462,469,470,527,528,529], and foams/microfoams [442,460,530,531]. Other 3D structures consist of dendritic carbon structure [532], graphene nanoscrolss [530], crumpled/wrinkled graphene [240,327,330,344,404,461,465,533], holey graphene [399], flakes [466], unstacked double-layer templated graphene [534], fibers [348,535], core-shell structure [337], vertically-aligned graphenes [351], and graphene nanoribbon networks [396,527].

Chen et al. [527] fabricated a highly conductive, ultralight, neat, and versatile nitrogen-doped graphene nanoribbons aerogel (GN_nRAe_) through a facile hydrothermal method for the first time. Graphene nanoribbons prepared by longitudinally unzipping multi-walled carbon keep the carbon skeleton’s structural integrity as well as present abundant active sites related to straight edges possessing an open bandgap that varies with the ribbon width. The hydrothermal treatment allowed assembly into a highly porous structure with a large surface area and good conductivity. GN_nRAe_ was revealed to be an efficient ORR catalyst in alkaline media (Table 3), with better stability in methanol than Pt/C, as well as in acidic media.

Graphene nanoribbons, a type of one-dimensional carbon material derived from carbon nanotubes (C_nT_), can serve as an ideal bridge [482,536,537] combining the features of graphene sheets and carbon nanotubes [527]; however, they are still limitedly applied. On the contrary, carbon nanotubes have been more widely used to obtain N-doped 3D graphene–C_nT_ composite materials [441,477,481,538,539,540,541,542,543,544,545,546,547,548,549,550,551]. The introduction of one-dimensional carbon nanotubes into graphene materials has different advantages, such as the formation of a highly conductive three-dimensional network to greatly enhance electron transport through a 3D-interpenetrating conductive network. The incorporation of C_nT_ can also solve the mass transfer issue caused by the stacking and agglomeration in graphene-based catalysts, which also reduces the number of active sites; moreover, the hydrophilic and hydrophobic nature of the dual interfaces can be conveniently tuned [539,542,551].

Qazzazie et al. [544] carried out a thorough investigation of N-doped graphene/carbon nanotube nanocomposites prepared by mixing N-doped graphene and C_nT_, consisting of a few layers of graphene flakes wrapped with C_nT_ network and separated from each other. Its ORR performance was examined by galvanostatic measurements in realistically approached glucose half-cells at neutral pH. It showed remarkable enhancement due to the improved accessibility of oxygen molecules to active reaction sites and the remove OH-; actually, the incorporation of C_nT_ between 2D graphene sheets opens mesoscale pathways for mass transport, forming a 3D porous structure in the nanocomposite. Efficient 4-electron transfer and improved overall catalytic ORR performance are observed as evident from voltammetric studies. 2D simulation models were carried out to clarify the effects of carbon nanotubes on mass transport and the overall catalytic activity of the electrode: the kinetics features extracted from the simulations show qualitative agreement with the experimentally determined kinetics underlining the role of C_nT_ acting as conductive spacers for improving accessibility of pores and providing more reaction sites for reactants in the nanocomposite.

In order to complete the overview on N-doped graphenes, it is important to report some studies that have strictly addressed the application of N-doped graphenes in fuel cells [552,553], the development of alternative mild and/or massive and/or environmentally friendly synthetic strategies of N-doped graphenes [554,555,556,557,558,559,560,561,562,563,564,565,566,567], and studied the effects of chemical or thermal treatment of N-doped graphenes [568,569,570].

The very last papers report theoretical studies that mostly concern DFT calculations for various heteroatom-doped graphene containing B, S, P, or co-doped species [128,361,571,572,573,574,575,576,577,578,579,580,581,582,583] and two papers on graphene characterized by structural deformation [584,585]. Xie et al. [585] compared the ORR reaction at active sites with different deformations under similar structural and chemical environments. Considering the associative mechanism of ORR, since both the tensile strain and the adsorption of O tend to break the NC* bond, the adsorption strength of the O atom is resonantly enhanced by the tensile strain, while those of OH and OOH remain unchanged. The results suggest that it is possible to improve catalytic performance by tuning the structure of the catalyst to be in selective resonance with the adsorption of a specific intermediate, which provides a new way for designing optimal catalysts.

## 5. Chalcogen (S, Se)-Doped Graphene

This section illustrates graphene doped with sulfur and selenium, the chalcogen elements belonging to group 16.

The first papers, published in 2012 [454,586], evidenced how the introduction of sulfur can boost the ORR electrocatalytic activity. Unlike nitrogen, boron, and phosphorus, sulfur has an electronegativity (2.58) close to that of carbon (2.55). Therefore, the enhancement of ORR catalytic activity in sulfur-doped graphene can be ascribed to the asymmetric spin density rather than to the change of atomic charge distribution [586,587], taking also into account that the d-orbitals of the embedded sulfur atoms are soft nucleophiles, and the local strain, due to the bigger size of S atoms compared to C atoms, favors the ORR reactivity around these sites [588].

**Figure 42:**
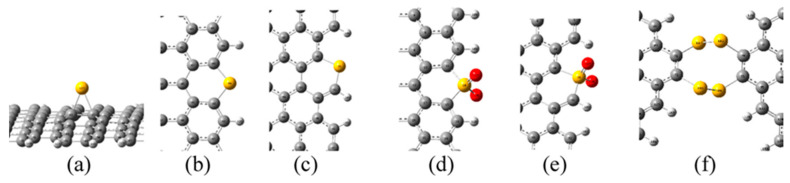
Several possible S-doped graphene clusters: (**a**) sulfur atoms adsorbed on the surface of the graphene cluster; substituting sulfur atoms at (**b**) zigzag and (**c**) armchair edges; SO_2_ substituted at (**d**) zigzag and (**e**) armchair edges; and (**f**) sulfur ring cluster connecting two pieces of graphene. The structures of the graphene are shown only partially to highlight the doping structures. Small white, gray, yellow, and red balls represent hydrogen, carbon, sulfur, and oxygen atoms, respectively. Reprinted with permission from [589]. Copyright {2014} American Chemical Society.

A few theoretical investigations [574,575,576,581,583,589,590,591] have been carried out on the catalytic processes involved in the ORR on S-doped graphene. Zhang et al. [589] conducted a specific study on catalytic mechanisms, proposing four types of sulfur-doping structures: surface S-adsorbed, edge S-substituted, edge SO_2_-substituted, and sulfur-ring connecting graphene clusters (Figure 42).

The S-doped graphene clusters with sulfur or sulfur oxide at graphene edges show electrocatalytic activity for ORR. Catalytically active sites are distributed at the zigzag edge or the neighboring carbon atoms of doped sulfur oxide atoms, which possess large spin or charge densities. For those systems where the sulfur atoms with the highest charge density are identified as the active catalytic sites, the ORR takes a two-electron transfer pathway, while in the case of the carbon atoms with high spin or charge density as active sites, it follows a four-electron transfer pathway. Moreover, evaluating the reaction energy barriers shows that sulfur-doped graphene is predicted to show ORR catalytic properties comparable to platinum, providing theoretical support for the discoveries made by Yang et al. [586], who prepared S-doped graphene by annealing GO with dibenzyl sulfide. The sample obtained at 1050 °C, characterized by a uniform distribution of S present in the plane and at edge positions, under alkaline conditions has an onset potential close to that of the Pt/C catalyst, the highest current density (at −0.8 V), and a comparable number of electron transfers, but with better stability and tolerance to methanol crossover than Pt/C.

In particular, since the theoretical demonstration that doping of a graphene sheet with S is possible with an average binding energy of 0.30 eV was published [591], there has been great effort in the development of efficient sulfurized metal-free ORR electrocatalysts, because the incorporation of S atoms into graphene requires a higher energy of formation than for the case of N or B atoms, making the synthesis of S-doped graphene more difficult [592].

The sulfur doping process is carried out at high temperature using single source precursors (thiophene [593], sulfonic resin [594], p-methylbenzenesulfonic acid [595], polyphenylensulphide [376]) or combining a C source (graphite/graphene derivatives, CH_4_, Na_2_CO_3_, and fructose) with different sulfur compounds [H_2_S, CS_2_, SO_2_, Na_2_S_n_, benzyldisulfide, phenyldisulfide, triphenyl sulphonium chloride, (NH_4_)_2_S_2_O_8_, KSCN, Na_2_SO_4_, Na_2_S_2_O_3_, H_2_SO_4_, 4-benzenedithiol dihydrochloride, sulfur, thiourea, polyphenylensulphide] as S source [128,273,454,531,596,597,598,599,600,601,602,603,604,605,606,607,608,609,610,611].

In particular, synthesis via an ion-exchange/activation combination method using a 732-type sulfonic acid ion exchange resin [594] produced 3D S-doped graphene networks (SG_3Dχ_) characterized by a high crystallization degree that contained appropriate sulfur doping (the sulfur content is as high as 12.8%), mainly in the form of –C–S–C–, which is beneficial for achieving high oxygen reduction. In addition, the S-doped graphene has an extremely high surface area, which can increase the number of catalytic sites exposed to oxygen molecules. Due to their unique structure and composition, the SG_3Dχ_ exhibited similar electrocatalytic activity but superior stability and methanol tolerance to the commercial Pt/C catalyst for four-electron oxygen reduction in alkaline solutions (Table 4).

**Figure 43:**
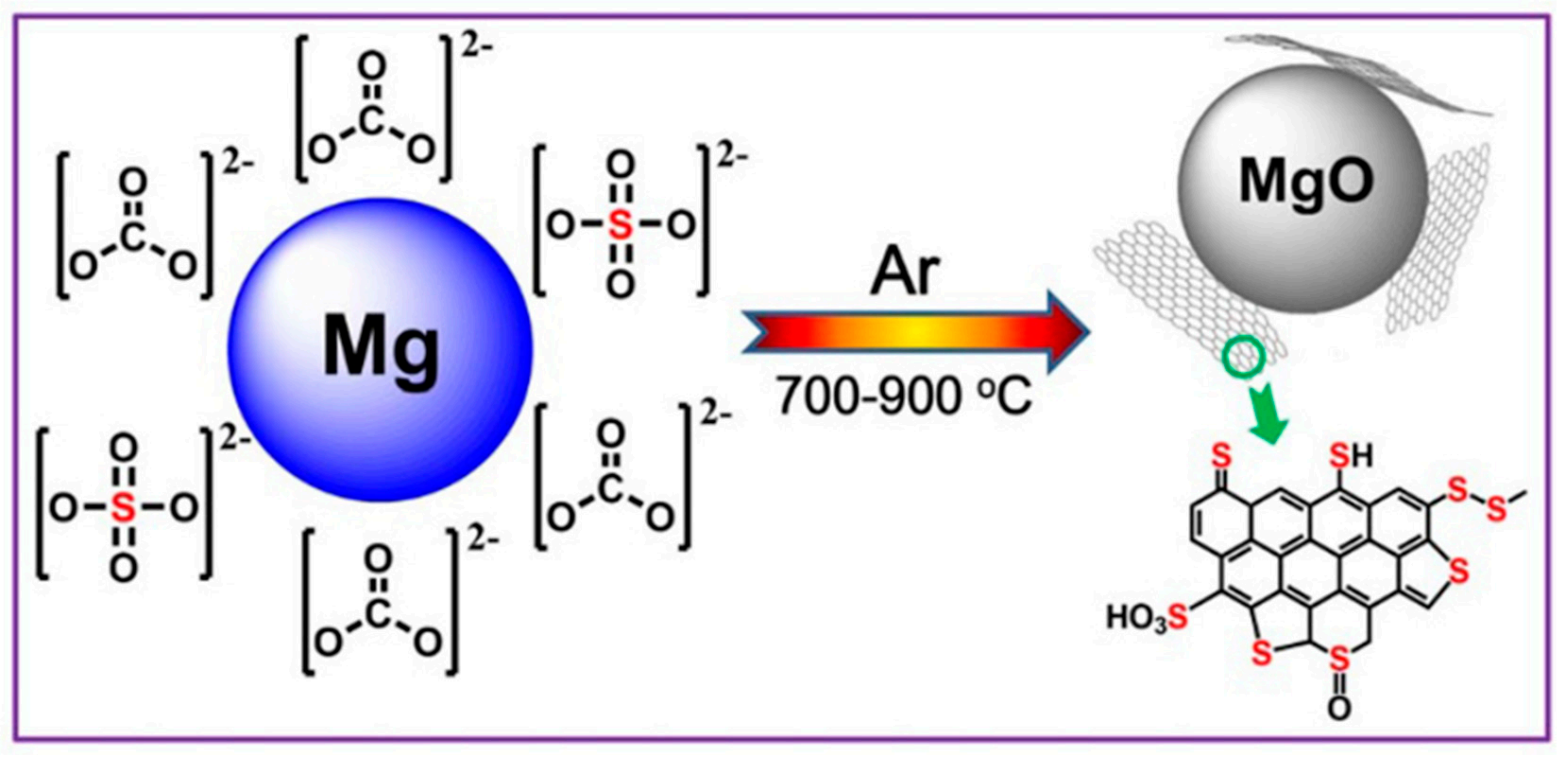
The schematic illustration indicating the production of S-doped graphene by magnesiothermic reduction of CO_3_^2^- ions in the presence of SO_4_^2^- ions in an Ar flow [602].

Another big challenge to directly generating S-doped graphene is its synthesis from easily available, low-cost, nontoxic inorganic salts, such as Na_2_CO_3_ and Na_2_SO_4,_ which are reduced by magnesium at high temperatures (Figure 43) [602].

This magnesiothermic reaction afforded S-doped graphenes characterized not onlyby high electrocatalytic activity for ORR with a four-electron reaction pathway, but also by much better stability and increased tolerance to MeOH crossover effects than commercial Pt/C (Table 4). The high ORR activity is mainly ascribed to highly graphitized structures, S-related active sites, and hierarchically porous textures.

However, other synthetic routes have also been developed to operate under mild conditions. Except for Ma et al. [612], who recovered S-graphene by continuous charge/discharge cycling of graphene–sulfur composites in Li–S batteries discharging and ball milling graphite with sulfur [502,587], alternative synthesis processes are carried out in solution [610,613,614,615,616,617,618,619], particularly under hydro/solvothermal conditions [120,588,614,619,620,621,622,623].

Chen et al. [620] first developed a low temperature (180 °C), economical, and facile one-pot hydrothermal method for synthesizing S-doped reduced graphene oxide nanosheets, S-rGO_nSh,_ 180, by reacting GO with Na_2_S, which was, therefore, employed not only as a sulfur source but also to reduce GO. Sulfur was distributed uniformly into the graphene plane and at the edges, mainly in the form of thiophenic S. S-rGO_nSh,_ 180, possessing numerous open edge sites and defects on its surface, showed superior electrocatalytic activity, long-term stability, and high methanol tolerance in alkaline media for ORR (Table 4). The authors speculate that the asymmetric spin density and the increase in edge plane defects play a key role in the observed ORR activity of S-graphene.

S-doped graphene nanosheets were synthesized by plasma-assisted [624] or simple electrochemical exfoliation of graphite using thiosulfate and H_2_SO_4_ as the sulfur source and the intercalation compound, respectively [610]. In this way, monolayer graphenes were obtained with fewer oxygen-containing functional groups (e.g., hydroxyl, carboxyl, carbonyl, and phenol groups) than conventional Hummers’ GO and had a less corrugated appearance as well. XPS showed that they contained sulfur substitutional defects, and sulfur doping provided materials with stable structural features. The main S_2p_ peaks at ca. 163.4 eV, 164.6 eV, and 168.3 eV were attributed to the contributions of H-S-C, R-S-C, and C-SO_x_-C (x = 2, 3, 4), respectively. In alkaline media, the ORR activity is correlated with a nearly four-electron pathway characterized by higher stability and methanol tolerance than a benchmark commercial Pt/C (Table 4).

Functionalization of graphene with sulfur groups can also be achieved under mild reaction conditions. Oligothiophene, sulfonic groups, and thiol groups were covalently bonded to graphene without altering the graphitic basal plane [625,626,627,628].

Jeon et al. [626] prepared large-scale edge-selectively functionalized graphene nanoplatelets via ball milling graphite in the presence of hydrogen, carbon dioxide, sulfur trioxide, or a carbon dioxide/sulfur trioxide mixture. Such edge-selectively functionalized graphene nanoplatelets are highly dispersible in various polar solvents, and it was found that the edge polar nature of the newly prepared graphene nanoplatelets without heteroatom doping into their basal plane played an important role in regulating ORR efficiency. The graphene functionalized with SO_3_H and SO_3_H/COOOH showed the best electrocatalytic activity, with ORR performance at high potential even better than Pt/C. The ionic interactions between strong acidic sulfonic acids and strong basic KOH in the electrolyte solution make the sulfonated graphenes more hydrophilic, and sulfonic acids at the edges can be fully ionized in an alkaline medium to form a strong negative charge. As a result, they have a strong affinity for electrolytes and oxygen.

**Figure 44:**
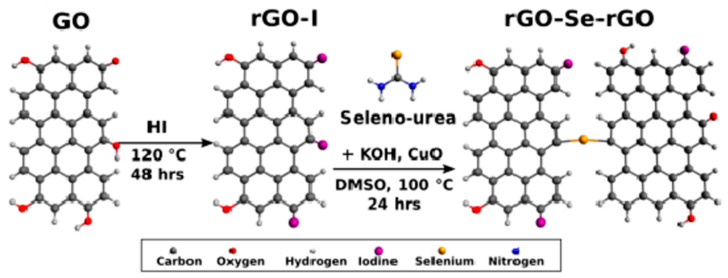
Schematic of the synthesis of rGOSe through a two-step procedure where GO layers are linked by selenium. Reprinted with permission from [629]. Copyright {2019} American Chemical Society.

In addition to the graphene functionalized with group 16 elements, the results obtained by Pal et al. [629] must also be mentioned. They coupled rGO-I with seleniothiourea (Figure 44).

The Se–graphene derivative has been evaluated as an ORR electrocatalyst. Since selenium is present in very small amounts (0.16% atom), the system is considered a single-atom site catalyst. Coupling with Se results in a prominent four-electron ORR pathway, similar to Pt/C (Table 4). DFT-based calculations show that Se bridging results in a charge density distribution in graphene, and the Se bridge causes a huge distortion in the lattice distance. Based on Hirshfeld charge analyses, Se is shown to possess a positive charge site, and hence, it is considered the active center in this ORR catalytic process.

To the best of our knowledge, only two papers in the literature [129,586] report information on proper selenium-doped graphene, even though selenium has similar chemical properties to sulfur. For example, its electronegativity (2.55) is close to that of sulfur (2.58), and the bond length and bond angle of C–Se–C in selenophene (C_4_H_4_Se) are almost equal to those of C–S–C in thiophene (C_4_H_4_S). On the other hand, selenium has a larger atomic size and higher polarizability than sulfur: lone selenium pairs can easily interact with molecules in the surrounding electrolyte.

In 2012, Jin et al. [129] fabricated selenium-doped carbon nanotube/graphene composites by facile thermal annealing of the carbon materials with diphenyl diselenide in argon, characterized by excellent catalytic activity, long-term stability, and a high methanol tolerance (Table 4). Se doping was confirmed by XPS and UV-Vis spectroscopy, showing that Se atoms are successfully introduced into the graphene framework via covalent bonds. The significantly enhanced catalytic activity observed is believed to be due to the increased electron transfer since the introduction of Se allows the restoration of defects at the edge of carbon materials and induces the formation of a π conjugation system.

## 6. Boron-Doped Graphene

Boron belongs to group 13 and is s immediately before C in the periodic table; boron atoms, possessing an atomic size similar to that of carbon and three valence electrons for binding with carbon atoms, can be introduced into the carbon lattice by substitutional doping. Boron doping into a carbon network gives the host carbon array p-type conductivity. Because of the lower electronegativity of B (2.04) compared to that of C (2.55), B atoms are positively polarized in the carbon lattice and hence able to attract the nucleophilic oxygen molecules, leading to efficient chemisorption; on the other hand, boron sites can also act as electron shuttles for the electron density of the graphite π-electron system going through the p_z_ orbital of boron to the chemisorbed O_2_ molecule [512,624].

The local positive charge density due to the incorporation of boron atoms into the graphene layer and enhancement of CO tolerance in the oxygen reduction reaction enables effective and stable electrochemical behavior [630]. B-doped graphenes have therefore attracted significant scientific attention.

Several theoretical studies have been carried out on the ORR process catalyzed by B-doped graphenes [128,571,572,574,575,577,578,581,582,631,632,633,634,635].

It must be considered that boron, which belongs to the same period as carbon, maintains the graphene surface basically flat, which helps in calculating the contributions of the electrons of the heteroatom to the delocalized density, even if it also depends on the adopted model [579].

Sheng et al. [636] first suggested that the electron-deficient boron atoms doped in graphene might function as active sites for oxygen adsorption and activation of the O–O bond cleavage. Based on the analysis of charge redistribution, the formation of active catalytic sites is attributed to the localized positive charge and electronic dipole induced by the dopant [574].

The kinetic pathways of oxygen reduction catalyzed by B-doped graphene have been analyzed. Fazio et al. [632] carried out a detailed study of the possible reaction paths for ORR as catalyzed by B-doped graphene through the identification of all the intermediates and transition structures for both dissociative and associative mechanisms, computing also the water contribution.

Among all the intermediate and transition structures that have been characterized along the various reaction coordinates, the most significant species is the end-on dioxygen species, an open-shell intermediate resulting from the adsorption of molecular oxygen on the positively charged B-doped atom. From the free energy diagrams at different electrode potentials derived by using the methodology developed by Nørskov et al. [637], they were able to determine the overpotentials both under acidic and alkaline conditions. In particular, at pH = 14, the overpotential is 0.28 V, which corresponds to an onset potential of 0.05 V, in excellent agreement with the experimentally reported value for ORR on B-doped graphene samples [128].

Despite the theoretical potential of B-doped graphene, from the experimental point of view, the literature on B-doped graphene tested as an ORR electrocatalyst is sparse, mainly because of difficulties at the synthesis level and low doping levels [638].

Synthesis of B-doped graphene has been mainly achieved at temperatures ranging from 400 to 1200 °C with various boron sources (boric oxide, boric acid, tetraphenyl phosphonium tetraphenyl borate, triphenyl borane, and Filmtronic B155 compounds) [385,531,636,639,640,641,642,643,644,645,646,647,648,649].

Zuo et al. [641] prepared a 3D B-doped porous graphene framework with hierarchical pores from frozen GO sediment containing boric acid via quick heating. The morphology of the porous graphene and the doping level can be effectively tuned by controlling the reaction temperature. During the heating, the frozen ice not only maintains the volatilization rate of water but also produces steam, which can introduce pores into the graphene sheet at high temperatures [650]. The nanopores are hence produced because of the strong oxidation ability of steam via the so-called water gas reactions at high temperatures. As for the catalyst, the novel hierarchical porous structure of the B-doped graphene framework can provide edge-rich planes and a large specific surface area with more active sites for ORR. The samples treated at 600 and 800 °C have ORR activity close to that of Pt/C, but with better durability against methanol.

3D B-doped graphene was also obtained using supercritical carbon dioxide, a one-pot and green method [651], where GO and borane adducts were mixed and reacted. The porous network structure of the products is likely the result of exfoliation and expansion of the rGO due to the action of the supercritical carbon dioxide fluid.

**Figure 45:**
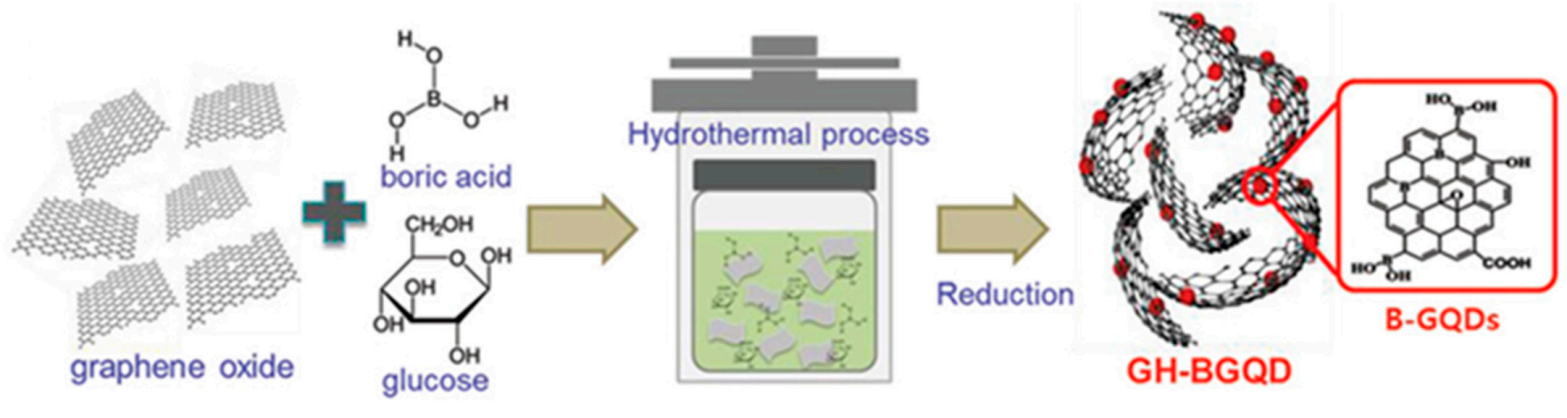
Schematic of the synthesis of B-doped graphene quantum dots [638], © 2019 WILEY-VCH Verlag GmbH & Co. KGaA, Weinheim.

The choice of operating under higher pressure allowed synthesis at a lower temperature under hydrothermal conditions [638,652,653,654]. Tam et al. [638] developed a simple and green one-pot synthesis method for novel nanocomposites consisting of a graphene hydrogel (GH) and B-doped graphene quantum dots (B-GQDs), (GH-BGQD), fabricating B-doped graphene quantum dots with a uniform size and a controlled boron concentration from glucose and boric acid (Figure 45).

XPS analysis identified various structures, such as the graphite-like BC_3_, and oxidized B-C bonds, such as BC_2_O and BCO_2_, to which the electrochemical behavior was correlated. Under alkaline conditions, the composite containing the higher percentage of BC_3,_ even if with a lower B-doping level with respect to another sample, showed the most positive onset potential (−0.05 V vs. SCE) and highest limiting current density (4.2 mAcm^−2^), which are close to those of Pt/C (−0.08 V vs. SCE and 4.6 mAcm^−2^) (Table 5). Theoretical calculations have evidenced that the adsorption of O_2_ on the BC_3_ structure in BGQDs is more favorable than those of the BC_2_O and BCO_2_ structures, as deduced from the calculated O_2_ adsorption energies of the BC_3_ structure (−0.190 and −0.204 eV) and those of the BC_2_O and BCO_2_ structures (−0.162 and −0.133 eV, respectively).

Moreover, the prepared GH-BGQD composites, which are characterized by a unique 3D architecture with high porosity and large specific surface area characterized by abundant catalytic active sites of BGQD as well as enhanced electrolyte mass transport and ion diffusion, exhibited superior trifunctional electrocatalytic activity toward the oxygen reduction reaction, oxygen evolution reaction, and hydrogen evolution reaction with excellent long-term stability and durability: a Zn–air battery and a water electrolysis cell were demonstrated using the GH-BGQD electrodes and were found to deliver a superior performance with a highly stable operation.

B-reduced graphene oxide obtained via hydrothermal reduction of GO [654] was recently integrated with activated carbon and carbon black as part of the MFCs’ cathode catalyst, significantly improving the output voltage and power density, with ameliorants of 75% and 58% over their undoped counterparts, respectively.

As an alternative to the reported hydrothermal reactions, boron-doping can also be achieved electrochemically to produce BG_qDot_ and three-dimensional graphene networks [512,655].

## 7. Phosphorus-Doped Graphene

Phosphorus belongs to group VA, similar to nitrogen, and possesses the same number of valence electrons; however, its electronegativity (2.19) is lower than that of carbon, which determines a consequent polarized distribution of charge density, a relevant feature for imparting ORR electrochemical properties to the phosphorus-doped graphene. Phosphorus has a relatively large atomic radius with respect to carbon, which makes the synthesis of P-doped graphene quite a challenge; hence, there are very few papers dealing specifically with experimental evaluation of synthesized P-doped graphene as ORR electrocatalysts compared with nitrogen-doped graphene [128,273,531,640,642,656,657,658,659,660,661,662,663,664].

The first papers on P-doped graphene and its electrochemical ORR properties were published in 2013 [656,657]. Zhang et al. [657] prepared P-doped graphene (GP) via a facile, low-cost, and scalable thermal annealing method using GO and triphenylphosphine as carbon and phosphorus sources, respectively. GP has a crystalline structure, and P was detected via EDX and XPS (about 1.3–1.8 at% P), indicating the presence of P-C and P-O bonds. GP exhibits outstanding ORR activity (close to Pt/C) as well as excellent selectivity and stability in alkaline media (Table 6).

A few theoretical studies have been carried out to elucidate the active sites and ORR mechanism in P-doped graphene [572,573,574,575,576,579,581,582,583,631,637,665,666,667]. Del Cueto et al. [572] developed a model to show that ORR via a four-electron transfer mechanism is energetically more favorable than the two-electron transfer mechanism. In addition, the energies calculated for each ORR step showed that the P-doped surface favors ORR better than the N and S-doped surfaces.

**Figure 46:**
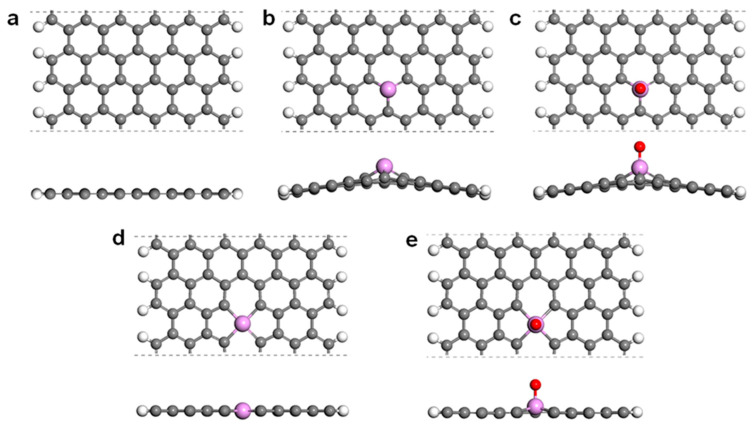
Top and side views of the different models of pristine and P-doped graphene: (**a**) pristine graphene (G), (**b**) PC3G, (**c**) OPC3G, (**d**) PC4G, and (**e**) OPC4G. The gray, white, red, and pink spheres represent the C, H, O, and P atoms, respectively. Reprinted with permission from [667]. Copyright {2017} American Chemical Society.

While Jiao et al. [128] showed that the active sites of ORR are the adjacent carbon atoms of heteroatoms, Zhang et al. [665] indicated that the P atom and its neighboring C atoms are the most preferred adsorption sites and the active center for the ORR. All of the ORR species adsorb on the P atom strongly, and all of the possible ORR elementary reactions take place within a small region around the dopant. Such divergence arises from the coordination and chemical bonding of P in carbon, which play an important role in ORR catalysis. Yang et al. [667] tried to provide a better understanding by considering the species experimentally, considering the possibility of P forming three to five bonds thanks to the hybridization of d orbitals (Figure 46).

Combining stability, conductivity, ΔG of the rate-determining step, and the number of potential active sites of the P-doped graphene structures, OPC3G was the most effective and stable P-doped graphene for ORR among the four types of P-doped structures—PC3G, OPC3G, PC4G, and OPC4G sheets.

## 8. Halogen-Doped Graphene

The halogen elements, fluorine (F), chlorine (Cl), bromine (Br), and iodine (I) belong to group 17 of the periodic table. They are all characterized by higher electronegativity than carbon, decreasing from F to I, and, except for fluorine, also have larger atomic sizes. They are also resonance-donating due to the existence of a lone pair of electrons.

Fluorine has strong electronegativity and theoretical calculations showed that by partially fluorinating graphene, the material energy band gap can be changed from 0.8 to 2.9 eV [668] and that a particular F-doped configuration is thermodynamically as good as Pt for ORR [581]; nevertheless, to the best of our knowledge, very few articles in the literature report on the evaluation of the ORR electrocatalytic properties of fluorinated graphene [669,670,671,672,673] mostly because of synthesis problems arising, for example, from safety issues. Kakaei and Balavandi [671] fluorinated graphene-based high-performance metal-free ORR electrocatalysts and optimized the amount of fluorine in rGO by mixing rGO in H_2_SO_4_ with NaF. They prepared F-rGO by randomly stacking graphene nanosheets with an interconnected 3D porous structure possessing C-F bonds, as clearly identified via IR. Doping with fluorine imparted better electrochemical activity, suggesting a 4-electron process for the best electrocatalyst in alkaline medium.

Moreover, focusing strictly on halogen-doped graphene, fluorine has been found to determine better ORR performance when used as a co-dopant with other halogens [671] and with just chlorine in halogen-doped graphene [674]. In the last case, F,Cl-rGO, synthesized from Cl_3_F as a single source precursor, is characterized by an onset potential of ORR close to that of the commercial Pt/C catalysts (40% *w*/*w*), but with much better tolerance to methanol crossover. F,Cl-rGO is a better ORR electrocatalyst than Cl-rGO, which contains only chlorine (Table 7).

There are few articles reporting on chlorine-doped graphene as an ORR electrocatalyst [245,671,675,676]. Zhang et al. [245] modified CVD graphene using the photochlorination method by irradiating with a Xenon lamp. Cl_2_ was generated in situ in a photoreactor containing vertically grown graphene on carbon cloth (Figure 19a).

This method induced the covalent attachment of Cl on the basal plane in a homogeneous way, generating a high degree of structural defects. Chlorinated vertically oriented graphene grown on carbon cloth exhibits improved ORR electrocatalytic activity, giving rise to more positive O_2_ reduction voltage and larger limited current density than benchmark electrocatalysts such as carbon cloth and pristine vertically oriented graphene grown on carbon cloth (Table 7). Moreover, chlorinated vertically oriented graphene grown on carbon cloth served as a bifunctional air-cathode for the construction of solid-state zinc air batteries, which showed stable discharge potential and high power and exhibited long-lasting rechargeability over 108 cycles at a current density of 2 mAcm^−2^, superior to noble metal-based zinc air batteries. The authors ascribe the good performance to the synergetic effects between defective sites, exposed edges, and the high electronegativity of Cl dopants.

It is, however, interesting that the high electronegativity of the dopant is not always the key factor for high ORR activity. Br- and I-doped graphenes were found to promote ORR more efficiently than analogous Cl-doped graphene [675,676] (Table 7), and therefore, the origin of ORR activity is thought to be related to the local electronic and bonding configuration around the dopant in the carbon lattice [677].

Jeon et al. [675], who prepared edge-selectively Cl-, Br-, I-doped graphene nanoplatelets (G_nPl_) via ball milling graphite in the presence of X_2_ (X =, Cl, Br, I), evidenced that the observed order of ORR activities ClG_nPl_ < BrGr_nPl_ < IG_nPl_ follows the atomic sizes (Cl atomic radius < Br atomic radius < I atomic radius) (Table 7). Hence, the valence electrons of Br and I are more loosely bound than those of Cl, facilitating charge polarization in the BrG_nPl_ and IG_nPl_ electrodes. Unlike Cl, Br and I can form partially ionized bonds of –Br^+^– and –I^+^. DFT calculations, performed for various edge configurations, showed that the edges of halogenated graphene have a favorable binding affinity with the O_2_ molecule, and the O-O bond is weakened because halogenation induces charge transfer, whose efficiency increases as the atomic radius increases.

To the best of our knowledge, apart from this paper and two others [671,676], no specific or further research has been conducted on Br-doped graphene. Among the halogenated graphenes, iodine derivatives are definitely the most studied [131,671,675,676,678,679,680,681,682,683,684].

Yao et al. [131] first utilized intercalated iodine graphene, obtained by annealing graphene oxide and iodine at 500–1100 °C in argon, as an ORR electrocatalyst, prompted by the already known application of iodine in conducting polymers [685,686]. Iodine is an important dopant that improves the electrical conductivity of materials, and graphene has a conjugate structure similar to that of conducting polymers.

As an alternative to molecular iodine [131,678,681,682] KI, NaIO_4_, HI, and deep eutectic solvents have been used to synthesize I-doped graphene [671,676,679,680,681,683,684,687,688]. Hoang et al. [684] exposed their hybrid to vapors of hydrogen iodide to simultaneously reduce the GO/C_Dot_ composite and introduce iodine dopants into the structure, while Marinoiu et al. [679,680,681] conducted solution-based electrophile substitution of KI/NaIO_4_ and graphene or a nucleophile substitution on graphene oxide using HI, also serving as a reducing agent [679,681,683]. The ORR activity of the iodine-doped graphenes was improved, and better performance of PEMFC was obtained when iodinated graphene was included as a microporous layer set between the catalyst and the gas diffusion layer [679,681,683] or in the cathode of the fuel cell with Pt/C [680].

It is interesting to observe that iodine doping occurred via the formation of iodine intercalated graphene, where I_3_- and I_5_- species are present, as detected using IR and XPS analysis. As already observed by Yao et al. [131], the presence of the I_3_- structure plays a crucial role in the enhancement of the ORR activity of graphene. In particular, a DFT study on polyiodide-doped graphenes by Hoyt et al. [689] showed that on the basis of band structure calculations and molecular orbital theory, the I_3_ molecule acts as a p-type dopant that shifts the Fermi level 0.46 below the Dirac point without noticeably disrupting graphene’s band structure. The I_3_ chains acquire charge in π* orbitals that are oriented with lobes perpendicular to graphene. Adsorption energy calculations reveal that I_3_ acts as an effective catalyst for the oxygen reduction reaction on graphene by stabilizing the rate-limiting OOH intermediate. I_3_ can serve as the active site for OOH adsorption and hence promote the early stages of oxygen reduction.

## 9. Multielement-Co-doped Graphene

The introduction of dopants into the carbon lattice has been the focus of many studies aimed at producing highly efficient electrocatalysts for the ORR via the alteration of charge or spin distribution of the sp^2^ carbon plane. Co-doping is considered an effective way of improving the electrochemical properties of the single heteroatom-doped graphene.

Different heteroatoms in the conjugated carbon backbone can create new non-electron-neutral sites, and except for very few cases [674,690,691,692,693], co-doped graphene always contains nitrogen as one of the heteroatoms.

The co-doped graphenes will be illustrated depending on the second heteroatom (S, Se, B, P, halogens) combined with nitrogen, including a subsection with the tri- and tetra-doped species.

### 9.1. N,Chalcogen (S,Se)-Co-doped Graphene

To adjust the bonding site of N in carbon nanomaterials, the addition of S as a further dopant helps to tailor the activity of the nanocarbon materials for the ORR through a higher degree of electron delocalization and polarization [586]. While nitrogen polarizes the adjacent carbons, the sulfur electronegativity characteristic and bulk size result in easy polarization and induce charge density/spin density on the surrounding graphene matrix, thus enhancing the ORR activity [694,695]. Furthermore, two lone-pair electrons of sulfur help in tuning the electronic band gap in the graphene framework to achieve a favorable electrochemical ORR. In addition, S-doped system alters the intrinsic hydrophobic nature of graphene to hydrophilic, and thus, it creates a strong affinity toward the adsorption of molecular oxygen, which results in enhanced ORR activity [696]. Remarkably, co-doping increases the density of active catalytic sites and induces a synergistic coupling effect in graphene layers, leading to further enhancement of the electrocatalytic ORR activity [697,698].

This effect has also been explained in terms of DFT calculation, where the ORR reactivity was related to the induced spin density after N,S-doping [596]. Song et al. [699] also showed chemical adsorption of the oxygen molecule on the N,S-co-doped graphene, which is much stronger than on either the mono N- or S-doped graphene.

The first design and preparation of N and S dual-doped mesoporous graphene as a metal-free catalyst for ORR in the literature was reported by Liang et al. [596]. Porous N,S-doped graphene was prepared via a very simple one-step doping process using solid and low-cost precursors (melamine and benzyl sulfide), in which porosity was introduced by employing commercially available colloidal silica. The catalyst showed excellent ORR performance comparable to that of commercial Pt/C as well as full fuel tolerance and much better long-term stability than Pt/C in an alkaline environment. DFT calculations revealed that the synergistic performance enhancement results from the redistribution of spin and charge densities were brought about by the dual doping of S and N atoms, which results in a large number of carbon atom active sites.

Right after the first successful co-doping of nitrogen and sulfur, the co-doping of N and Se of a graphene carbon nanotube composite was reported [700]. Se-doping demonstrated much stronger effects on ORR activity compared to S-doping, but the research on co-doping graphene with nitrogen and selenium did not advance.

On the contrary, a few more papers appeared on N,S-co-doped graphene–C_nT_ composites [701,702].

Villemson et al. [702] proposed a novel and effective one-pot synthetic approach to prepare N,S-doped carbon catalysts by using heat treatment of the GO and multiwalled C_nTs_ mixture, together with o-methylisourea bisulfate. The composite showed superior ORR performance in alkaline media (Table 8), and the electrocatalytic mechanism for the reduction of oxygen was well explained by DFT calculations of graphene sheets according to the XPS data and ORR test results. The C_α_ and C_β_ carbon atoms at the graphitic nitrogen located close to the sulfur atom correspond to the global minimum and are thereby energetically most favorable (−1.14 eV) for oxygen electroreduction.

**Scheme 5:**
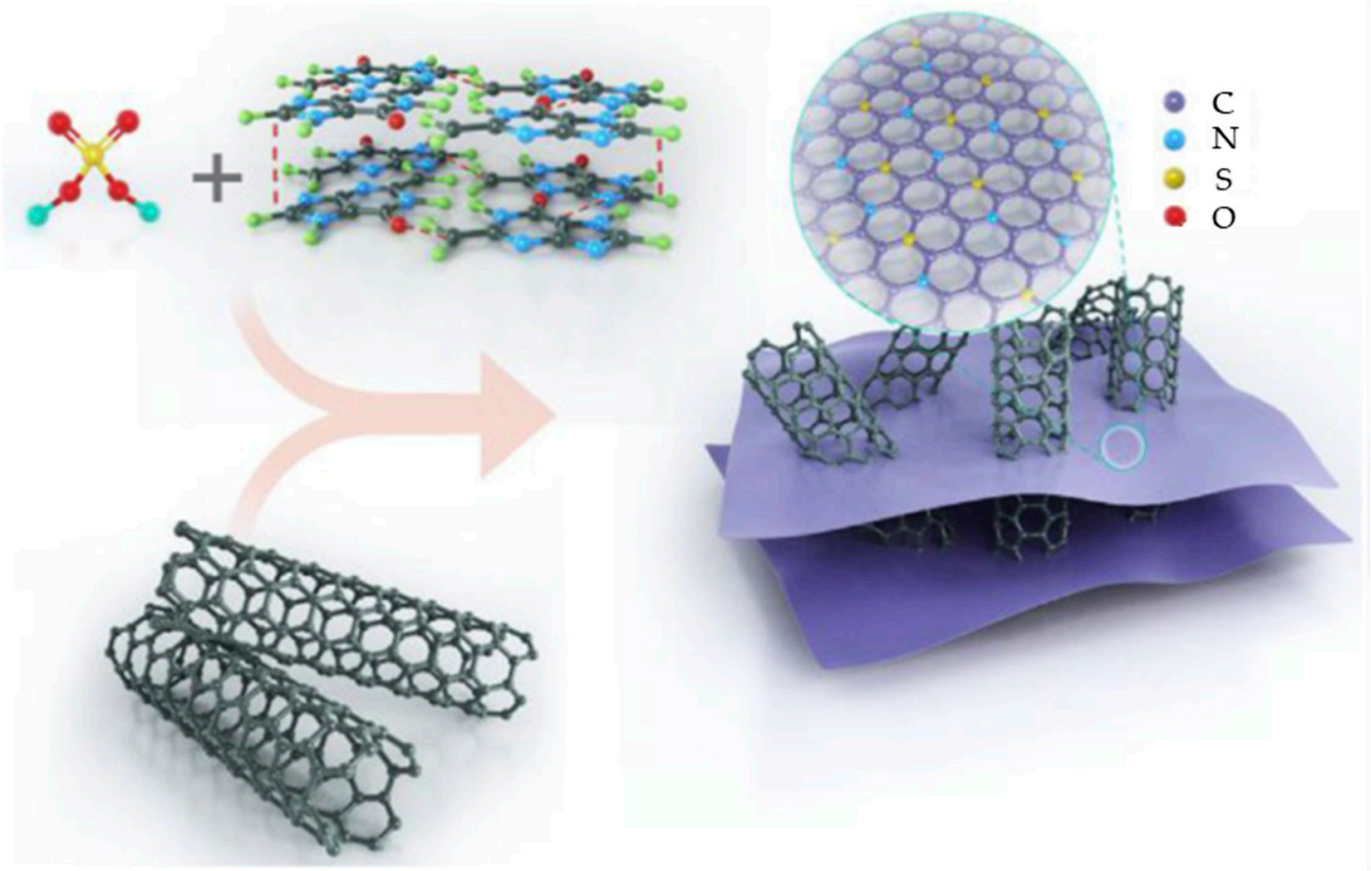
Illustration of the synthesis route for C_nT_/N,S/graphene_3D_. Adapted from [701]. © 2019 Elsevier B.V (2019).

Recently, Huang et al. [701] reported C_nT_-N,S-co-doped graphene-based 3D carbon composites (C_nT_/N,S-graphene_3D_) with high activity toward a set of important electrochemical reactions and high performance in Zn–air batteries. In the composite, graphene is found to strongly couple with the surface of C_nTs_, achieving uniform distribution of both components and combining optimal nitrogen configuration (graphitic and pyridinic) and well-developed porosity. The effective integration of 1D C_nTs_ and 2D graphene into 3D conductive frameworks was achieved in a facile and sustainable preparation via an in situ direct pyrolysis in the presence of C_nT_ of a layered biomolecule guanine and guanine sulfate (Scheme 5), which typically exists in the form of a layered structure through intermolecular H-bond interactions.

The strategy of using just one compound as a nitrogen and sulfur source is the most exploited one, thanks to the large availability of reagents [209,608,609,614,623,658,701,702,703,704,705,706,707,708,709,710,711,712,713,714,715,716,717,718,719,720,721,722,723,724,725,726]. The preferred procedure is based mainly on the thermal treatment of suitable precursors [608,658,701,702,703,704,705,706,708,711,715,717,719,720,721]. Pan et al. [713] reported the facile and convenient preparation of N,S-co-doped graphene from low-cost and green raw materials, consisting of the pyrolysis of cysteine in the presence of NaCl, which serves as a structure-directing template to direct the growth of graphene with the formation of curved graphene domains. The product has remarkable ORR activity based on an almost 4-electron transfer process (Table 8). Arunchander et al. [720] carried out simultaneous doping of nitrogen and sulfur using in situ polymerization of 6-N,N-dibutylamine^−1^,3,5-triazine-2,4-dithiol on a graphene framework and subsequent pyrolysis. The sample treated at 1000 °C (N-S-Gr^−1^000), exhibiting an enhanced ORR activity dominated by the 4-electron pathway compared to other catalysts (Table 8), was the first N,S-co-doped graphene whose potential as a cathode catalyst was validated in a membrane electrode assembly and in a real AEMFC. A peak power density of 20 mWcm^−2^, which is ~3.4-fold lower than that of the Pt/C catalyst, was achieved under ambient temperature and pressure, which makes N-S-Gr^−1^000 a promising alternative nonprecious metal catalyst in AEMFCs.

On the other hand, good electrocatalysts based on N,S-co-doped graphenes can also be obtained under mild reaction conditions [209,609,614,623,707,716,718,723,727]. Wu et al. [722] prepared N,S-co-doped graphene aerogel with a 3D hierarchical porous structure and high specific surface area from the amino acid methionine and GO under hydrothermal conditions and subsequent freeze-drying. Methionine acted as a reductant, as well as an N and S source. The N,S-co-doped graphene aerogel exhibited good mechanical properties, excellent capacitive performance, and ORR performance, with selectivity tending towards a 4-electron process with increasing voltage. Favaro et al. [293] used an electrochemical approach to synthesize doped graphene quantum dots via electrochemical etching of GO with cysteine. It was observed that the reaction selectivity is controlled by the oxidation degree of the materials: as-prepared graphene oxide quantum dots, which have highly oxidized functional groups, follow a two-electron reduction pathway and produce hydrogen peroxide, whereas after reduction treatment with NaBH_4_, the same materials favor a four-electron reduction of oxygen to water.

N,S-co-doped graphene quantum dots were obtained in the last few years by hydro- or solvothermal treatment [376,637]. In the presence of NH_4_OH and Na_2_S as precursors, Fan et al. [619] synthesized N,S-G_qDots,_ which possessed enhanced electrocatalytic activity compared to G_qDot_, NG_qDot_, and SG_qDot_ and their composite with GO, even comparable to Pt/C (Table 8). The introduction of S changes the state of N species in N,S-G_qDots_, creates asymmetrical spin and charge density, and enables the synergetic effect of N and S, which facilitates the electron transfer in the ORR process.

It is interesting to observe that the use of two compounds as separate sources of nitrogen and sulfur opens the opportunity for a one [376,593,596,608,617,618,728,729,730,731,732,733,734] or two-step doping process [598,604,607,735]; however, except for very few examples [617,618,703,730], thermal treatment at high temperature is the common synthesis method. The temperature approach is commonly used to tune the features of the active centers during material fabrication for ORR performance improvement.

**Figure 47:**
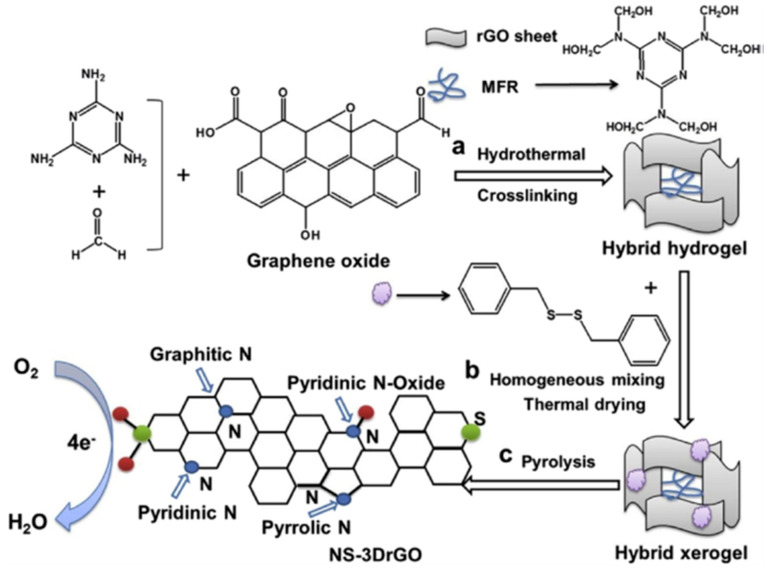
Schematic illustration of the fabricated NS-3DrGO products: (**a**) crosslinking of GO by the addition of melamine and formaldehyde using a hydrothermal method; (**b**) homogenous mixing of benzyl disulfide with the as-prepared hybrid hydrogel; (**c**) pyrolysis of the hybrid xerogel to synthesize the N,S co-doped 3D rGO catalyst from [735]. © 2017 Elsevier Ltd. (2017).

Li et al. [735] developed a well-structured process (Figure 47) where crosslinking of GO by the addition of melamine and formaldehyde occurred using a hydrothermal method; after the addition of dibenzylsulfide and pyrolysis, 3D N,S-co-doped graphenes (NS-3DrGO) were obtained.

The sample obtained at 950 °C had the optimal ORR activity compared with the others (Table 8), which may be due to highest amount (74.8 at.%) of the two active nitrogen species (pyridinic N and graphitic N) and the highest amount (79.8 at.%) of active thiophene-S together with the desirable specific surface (391.9 m^2^g^−1^) area and multi-porous structure. Furthermore, the N,S-3D rGO catalysts also exhibit superior methanol tolerance and favorable durability.

Moreover, very recently, Zhang et al. [734] were able to tune the configuration between graphitic-N and thiophenic-S dopants in one-step-synthesized graphene nanosheets by selecting suitable S precursors, obtaining a material which performed well in a Zn–air battery as cathode, producing a high-power density of 146 mWcm^−2^.

### 9.2. N,B-Co-doped Graphene

Co-doping with nitrogen, which has a higher electronegativity (3.04), and boron, which has a lower electronegativity (2.04) than C (2.55), can create a unique electronic structure with a synergistic coupling effect between the heteroatoms.

The introduction of N and B in graphene systems was predicted to enhance ORR activity [297,342] even before any experimental proof; this was first achieved by Wang et al. [736], who developed a facile low-cost approach for mass production of N,B-co-doped graphene with tunable N-/B-doping levels simply by thermal annealing graphene oxide in the presence of boric acid under ammonia atmosphere. The resultant N,B-co-doped graphene samples showed ORR electrocatalytic activities that were even better than the commercial Pt/C electrocatalyst, and with an electron transfer close to 4. DFT calculations indicated a model with a low bandgap and therefore high conductivity; however, it also had many more carbon atoms with relatively high spin density and charge density compared to pure graphene, thus providing more active sites to catalyze ORR: the theoretical results agreed with the experimental data.

It is interesting to observe theoretical research on the mechanism of ORR catalyzed by N,B-co-doped graphene, analogous to what is reported for the single B-doped graphene [512,572,574,577,578,639,648,737,738,739,740,741,742,743].

The BN systems have been analyzed considering mostly different BN clusters; Sinthika et al. [577] analyzed a co-doped graphene containing a simple BN cluster. They identified a descriptor of N,B-co-doped graphene ORR performance, the variance of the average of p_z_ occupancies of the carbon atoms adjacent to boron, finding a linear correlation with the free energy of OH adsorption. Kattel et al. [737] identified graphitic BN_3_ motifs as active sites for complete O_2_ electroreduction in the N,B-co-doped graphene electrocatalyst. Increasing N coordination to B gradually shifts the band gap below the Fermi level, so that graphene with the BN_3_ defect motif has a small band gap below the Fermi level, showing the increase in electron (donor) concentration states. On the other hand, Tang et al. [740], considering that the BN clusters embedded in graphene involve different sizes and shapes as well as edge termination, discussed the effect of edge termination and the shape of substitutional BN clusters on oxygen dissociation barriers; they found that N-terminated triangular BN (t-BN) cluster doping can reduce the energy barrier more effectively compared to a t-BN with a B edge or quadrangular BN cluster. The enhancement of catalytic activity by triangular BN doping is attributed to the positively charged active site and its large density of unoccupied electronic states around the Fermi level.

However, from the experimental point of view, the presence of BN bond, and in particular the formation of hexagonal boron nitride, is sometimes recognized as detrimental for the ORR performance, with a preference for a structural B-C-N configuration. This is the reason why Zheng et al. [639] developed a two-step co-doping method: GO was annealed with NH_3_ at an intermediate temperature (e.g., 500 °C), and then B was introduced by pyrolysis of the intermediate material (N-graphene) with H_3_BO_3_ at a higher temperature (e.g., 900 °C), enabling the incorporation of heteroatoms at selected sites in the graphene framework to induce a synergistic enhancement of the activity of the N,B graphene. In this way, the formation of inactive by-products was prevented, and the new catalyst showed excellent activity in the ORR and perfect (nearly 100%) selectivity for the four-electron ORR pathway in an alkaline medium (Table 8). The better ORR performance than that of the single boron and nitrogen-doped graphenes was ascribed to the synergistic coupling effect between heteroatoms, which arises from the charge transfer process in which N has the role of an electron-withdrawing group, indirectly activating B, and thus makes the latter an active site to enhance the ORR activity.

The importance of the presence of the B-C-N hetero-ring or the absence of BN bond to achieve the synergistic effect has been recognized by other authors [645,649,742,744,745,746], who accordingly analyzed a dual-step synthesis procedure using two separate N and B sources in order to avoid direct combination of the heteroatoms. In this perspective, Qin et al. [742] succeeded in controlling BN configurations and producing N,B-co-doped porous graphene with ORR catalytic activity comparable to that of Pt/Calso in acidic media (Table 9).

The use of different nitrogen and boron sources is the most preferred way for co-doping materials, mainly because of the larger availability of compounds [177,512,639,644,645,646,648,649,655,739,742,744,745,747,748].

Gong et al. [739] built 3D interpenetrating networks from numerous flexible ribbons and showed the importance of an optimal doping level of boron and nitrogen in graphene nanoribbons to obtain a material with excellent ORR electrocatalytic activity, even better than the commercial Pt-C catalysts (Table 8). A low BN content results in a low number of active sites, while BN content that is too high undermines the conductivity of the material, weakening the charge transport from the electrode to oxygen and decreasing electron transfer.

Even if less exploited, the strategy of using a single source of N and B for doping may have the advantages of energy-saving, less time-consuming, less waste of precursor, and less complicated operating apparatus [208,415,643,746,749,750,751].

Jiang et al. [643] prepared boron and nitrogen-co-doped hollow graphene microspheres using a simple template sacrificing method in which doping of the amino-modified SiO_2_ nanoparticles supported GO with N and B occurred in the presence of NH_3_BF_3_ under hydrothermal conditions. Silica was then removed through HF etching. The as prepared microsphere present high electrocatalytic activity (Table 8), which could be attributed to the synergetic effect of the N,B-co-doped graphitic structure and the specific microspherical hollow morphology, which promotes the exposure of more surface area accessible to electrolytes and decreases the overpotential for the ORR by reducing the contribution from the mass transport limitation.

Other porous structures consisting of graphene aerogels with tunable contents of B and N configurations were obtained by Chen et al. [751] from NH_4_B_5_O_8_ using a hydrothermal method and freeze-drying process. Linear sweep voltammetry results confirmed that the increasing doping contents of pyridinic N and BC_3_ phases in N,B-doped graphene aerogels can boost ORR activity (Table 8). The proposed co-doped aerogels not only show similar ORR activity to commercial Pt/C, but also superior stability and excellent methanol tolerance; moreover, the performance was appreciable when it was tested as an air cathode for a rechargeable zinc–air battery device.

N,B-doping can also be achieved by functionalizing graphene with L-cysteine [752] or with amino-functionalized boron nitride [753] via cross-linking.

### 9.3. N,P-Codoped Graphene

Phosphorus like boron, has lower electronegativity than carbon, disrupts the charge uniformity of carbon, creates new charge and structural defect sites, and increases the electron delocalization [754]. On the other hand, like sulfur, it has a relatively larger atomic size than that of C, and consequently produces large defect sites, more edge sites, and high curvature in the carbon lattice [755,756].

This system and the N,P synergistic effect has been studied via DFT calculations [573,663,757] but starting from different models; in one, Gracia-Espino et al. [573] considered substitutional P and substitutional N separate atoms, while in the other one, Xue et al. [757] showed that N and P atoms are inclined to bond with each other, forming N-P clusters in graphene. Gracia-Espino et al. [573] illustrated that the possibility of quantifying and determining the spatial distribution of catalytic sites permitted the construction of reduced overpotential (η_ORR_) maps; such maps provided an insight into the source of the synergistic effect of N,P-co-doped graphene commonly reported in the literature. Xue et al. [757] evidenced that the catalytic performances of the N-P clusters are sensitive to their geometries, and especially to the N:P ratios.

**Figure 48:**
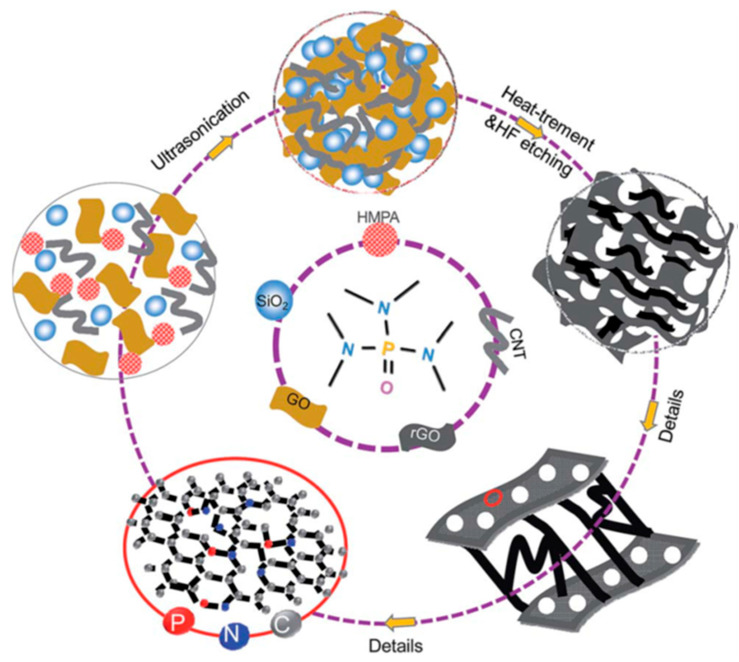
Schematic illustration of the synthesis procedures for 3D porous carbon nanotube-graphene hybrid foam with embedded N,P-coupled active species. Adapted from [660] with permission from the Royal Society of Chemistry. https://pubs.rsc.org/en/content/articlelanding/2018/ta/c7ta08186c (accessed on 1 March 2025).

Experimentally, the existence of P-N moieties and their catalytic effects have been reported by Li et al. [660], who successfully constructed 3D porous carbon nanotube/graphene hybrid foams with embedded N,P-coupled active species by one-pot pyrolysis method (Figure 48) using graphene oxides and carbon nanotubes as building bricks and hexamethylphosphoric triamide (HMPA) as the special source of P and N.

Impressively, the HMPA compound not only facilitated the remarkable increase in the content of both P and N in the carbon frameworks but also offered a high density of the coupled N-P moieties embedded in the graphene surface. Together with the optimized local structures, including open porosities and high electron transportation capacity, this foam exhibits excellent electrocatalytic performance for the ORR in acidic medium. In particular, the ORR onset potential and half-wave potential reached 0.98 and 0.78 V in 0.1 M HClO_4_ (Table 9).

In another research, Yang et al. [758] prepared N,P-co-doped graphene-based electrocatalysts with high activity also in acidic media (Table 9). They assembled carbon nanotubes and graphene into hybrid nanospheres via a one-step aerosol route, which, after doping with nitrogen and phosphorus, showed higher oxygen reduction reaction activity, better stability, and better methanol tolerance for ORR than the commercial Pt/C catalyst in alkaline solution.

**Scheme 6:**
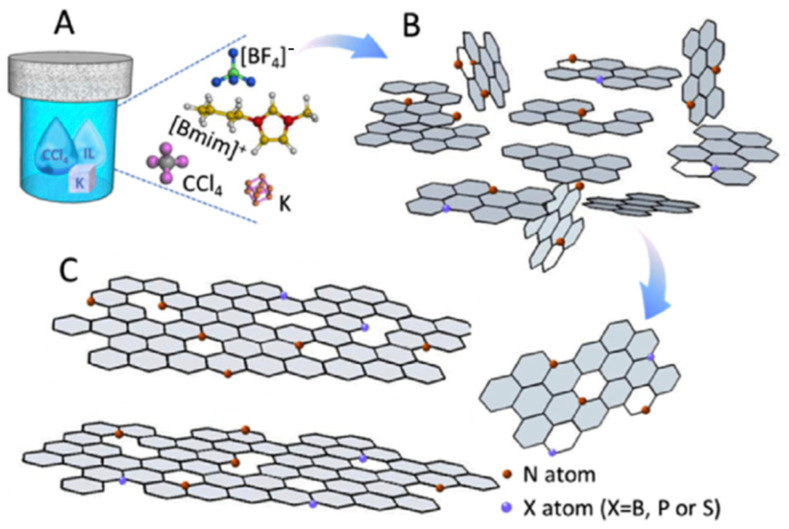
Proposed mechanism for the synthesis of N, X (B, P, or S) dual-doped graphene. Adapted from [208]. © 2016 Elsevier Ltd. (2016).

N,P-co-doping was mainly carried out at high temperature, which was necessary to push the large phosphorus atom into the graphene backbone or to form P-C on the edge [660,662,663,758,759,760,761,762,763,764,765,766,767]. There are very few papers dealing with synthesis under mild conditions [208,661,768,769]. Ma et al. [208] reported a safe, facile, and feasible solvothermal method for synthesizing N, X (X = B, P, S) dual-doped graphene by utilizing CCl_4_ and metal K as precursors and introducing ionic liquids into a Wurtz-type reductive coupling reaction (Scheme 6).

Ionic liquids, which are easily soluble in various solvents [770,771], play a dual role in the fabrication process: on one hand, the ionic liquids containing an organic, nitrogen-containing cation and a bulky inorganic anion can provide two or more doping elements with target materials. On the other hand, the released gas resulting from the decomposition of ionic liquids will act as a porogenic agent, giving rise to some pores in the resultant doped graphene. The improved electrochemical performance of all co-doped graphene samples indicates that dual-doping actually exerts a positive influence on electrochemical activity, which could be regarded as the synergistic effect. All three types of co-doped graphene have good selectivity for a four-electron pathway. Among them, N,P-co-doped graphene exhibited the best catalytic activity in terms of half-wave potential, methanol tolerance, and long-term durability (Table 8). This could be attributed to the change of charge density and high distortion of carbon structures resulting from the combination of the large electronegativity of N and the large covalent radius of P.

### 9.4. N,Halogen-Co-Doped Graphene

Fluorine and iodine are the only halogens used for co-doping graphene with nitrogen.

While in the case of a co-doped sulfur iodine graphene [690], the halogen is introduced via a well-defined mechanochemical ball milling, co-doping with nitrogen was achieved mainly via thermal treatment of graphene with iodine [678,772].

**Figure 49:**
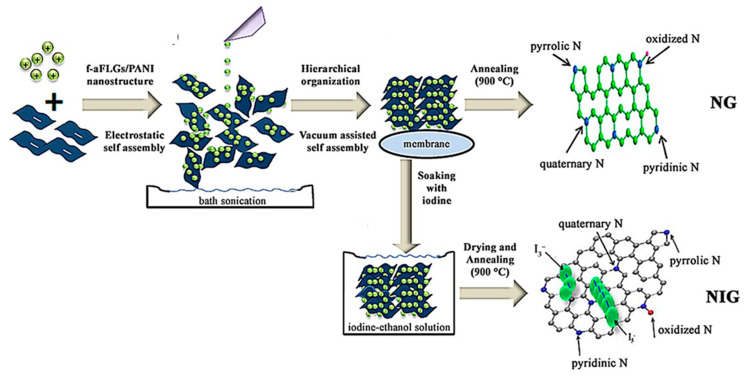
Schematic representation of the synthesis of iodine and nitrogen co-doped graphene [772] © 2014 Wiley-VCH Verlag GmbH & Co. KGaA, Weinheim.

Hassan et al. [772] performed, for the first time, iodine and nitrogen co-doping of graphene using iodine, poly(aniline) (PANI), and activated graphene as starting materials (Figure 49).

Compared with N-doped graphene (NG), iodine and nitrogen co-doped graphene (NIG) exhibited enhanced surface area, resulting in a direct four-electron reaction pathway, high onset potential, and high current density as well as improved resistance to methanol poisoning and superior catalytic durability for ORR in alkaline medium. The synergistically enhanced ORR performance of NIG was found to be a result of a high strain and size advantage of the larger iodine atom clusters (compared to nitrogen), which facilitate the simultaneous enrichment of anode electrons and O_2_ and H_2_O molecule transport at catalytic sites, inducing four-electron transfer in a single step.

Simple reduction of graphene oxide with 4-iodophenylhydrazine produced nitrogen and iodine co-functionalized graphene oxide with enhanced dispersibility in organic solvents and electrochemical ORR activity because of the electronic modifications of the graphene structure [773].

Analogously, graphene sheets functionalized with amino groups and fluorine in an out-plane scenario were obtained by Zhao et al. [774]; optimization of the ratio of NH_2_ and F groups allowed the achievement of a material with outstanding electrocatalytic properties, even delivering slightly better electrocatalysis than commercial Pt/C catalyst (Table 8).

Such materials were developed as alternatives to the other F,N-co-doped graphenes that have appeared since 2014 [669,670,692,775,776,777,778,779].

Vineesh et al. [669] developed bulk nitrogen and fluorine-doped graphene (FNG) via a simple method. The obtained FNG is characterized by enhanced electrocatalytic efficiency (Table 8) with respect to the N and F individually doped graphene, benchmarked electrocatalyst—Pt/C and other reported doped graphene systems, which is ascribed to the synergistic effect of the heteroatoms. In order to understand the synergistic doping effects and the role of ‘electron spin density’ in determining the catalytic efficiency of graphene, DFT calculations were carried out. According to the DFT calculations, FNG with all three types of nitrogen at close proximity exhibited the most catalytic activity towards ORR. The order of activity towards ORR deduced from the DFT calculations follows the order FNG > FG > NG and is in excellent agreement with the experimental results.

Qiao et al. [776], who prepared reduced graphene oxide co-doped with nitrogen and fluorine in a simple one-step thermal annealing process using a mixture of graphene oxide and ammonium fluoride in an argon atmosphere, evidenced that the additional doping with F considerably enhanced the performance of the catalyst. This could be attributed to fluorine’s high electronegativity and presence in the form of the most active semi-ionic C-F bond, which could have (i) increased the adsorption of oxygen, (ii) activated O-O bond cleavage, and (iii) formed a synergetic effect with the N dopant.

In any case, fluorine plays a determining role in activating the graphene systems not only when co-doped with nitrogen but also with boron. Nitrogen and fluorine-co-doped graphene and boron and fluorine-co-doped graphene prepared via a simple one-pot hydrothermal treatment method exhibit analogous catalytic and definitely better than the respective single-doped species [690].

### 9.5. Tri- and Tetra-Doped Graphenes

Multiple heteroatom species doping not only introduce more active sites but also generate new properties or create synergistic catalytic effects [780,781], a point that Wang et al. [782] showed in a bit of a satiric way in 2020. To complete the description of the various doped graphenes, this subsection reports graphenes with three or four different heteroatoms, consisting of nitrogen in combination with phosphorus and sulfur [658,783,784,785], with phosphorus and boron [691,786], with sulfur and fluorine [616], and with phosphorus, boron, and sulfur [273,787].

Graphene doped with N,P and S is the most studied tri-doped system and was the first to be reported [658]. P dopant is known to disrupt the charge uniformity of carbon, create new charge and structural defect sites, and increase the electron delocalization. On the other hand, S dopant can induce an enhanced spin density of carbon and polarizability and increase structural defects. Furthermore, these two elements, which have relatively larger atomic sizes than C, produce large defect sites, more edge sites, and high curvature in the carbon lattice [755,756]. Additionally, topographic defects produced under high temperature treatment may be another effective factor for improving onset potential.

**Figure 50:**
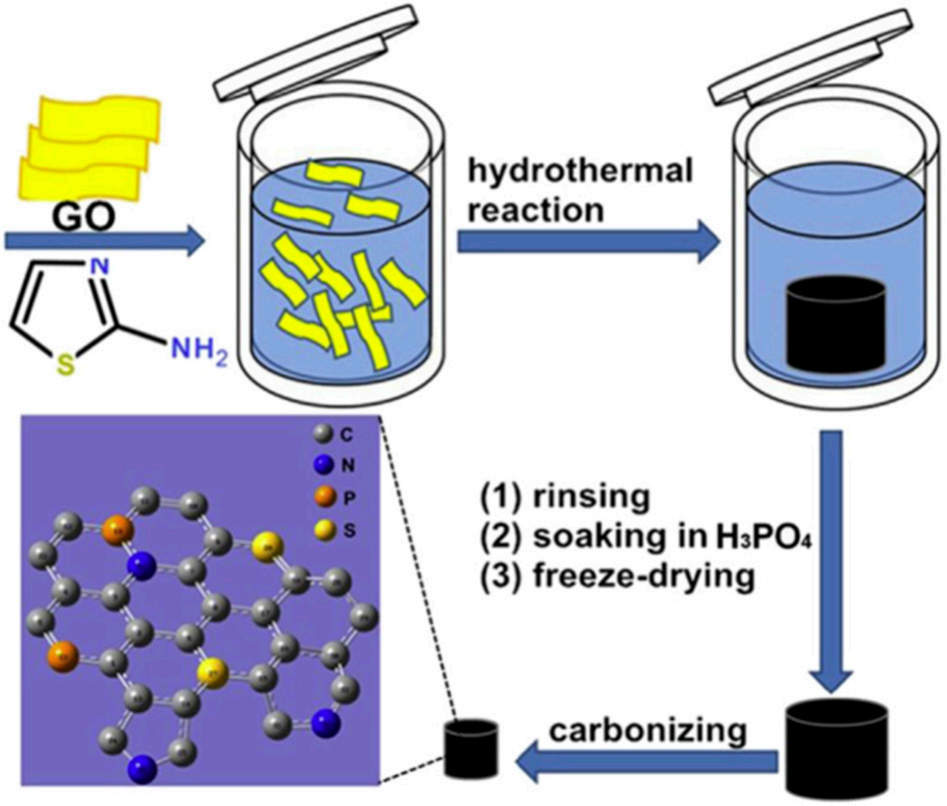
Schematic illustration of the preparation of N,S,P-doped graphene. Adapted from [715]. © 2017 Elsevier B.V. (2017).

Fan et al. [715] developed N,S,P-tri-doped graphenes from N,S-co-doped graphene, prepared by hydrothermal process of GO with 2-aminothiazole as the sulfur and nitrogen source, soaked (in phosphoric acid), and freeze-dried before final carbonization under N_2_ (Figure 50).

Fan et al. showed that multiple heteroatom species doping is beneficial for improving the ORR catalytic properties (Table 8); with increasing heteroatom species, the onset potentials of graphene positively move, and the electron number approaches the 4e pathway by the synergistic effect of different heteroatom species.

## 10. Graphene and Reduced Graphene Oxide

In this section, we deal with pristine graphene and reduced graphene oxide, in which the sp^2^ carbon skeleton may contain oxygen in variable amounts.

Evidence of ORR activity of graphene sheets in alkaline media was first given in 2009 by Tang et al. [788]; later on, ORR activity was also shown in acidic [789] and neutral media [790,791] (Table 10).

It is interesting to observe that in the early years, appreciable electrocatalytic activity in acidic and neutral media was achieved using graphene functionalized with covalently attached anthraquinone derivatives. The π-conjugated system and the quinone group could synergistically interact to enhance the electrocatalytic performance for O_2_ reduction [791,792,793] (Table 9). The highly conductive rGO provides convenient channels for charge transfer, thus resulting in more favorable electron transfer kinetics, which could also be achieved by linking directly C_60_ [794].

As an alternative to functionalization, a way for improving the electrochemical properties consisted of enriching graphene with edges and defects in order to perturb the electron configuration and activate the carbon π electrons for effective utilization by O_2_ [496,506,795,796,797,798,799,800,801,802,803,804,805,806,807]. It was demonstrated that CVD-grown high-quality graphene consisting of almost defect-free multilayer graphene on Ni substrate was rather inactive for oxygen reduction in alkaline medium compared with bare glassy carbon [808].

The role of lattice defects has been well illustrated by Zhang et al. [809] who showed via DFT that a pure graphene cluster could efficiently facilitate the ORR by introducing a point defect like a pentagon carbon ring at the edge or a line defect like a pentagon–pentagon–octagon grain line. These defects interact with the edge structure to generate active sites for ORR catalysis. The four-electron and two-electron ORR can occur on these defective graphenes simultaneously, and these sub-reactions are energetically favorable because the reaction-free energy of the sub-reactions is negative.

**Figure 51:**
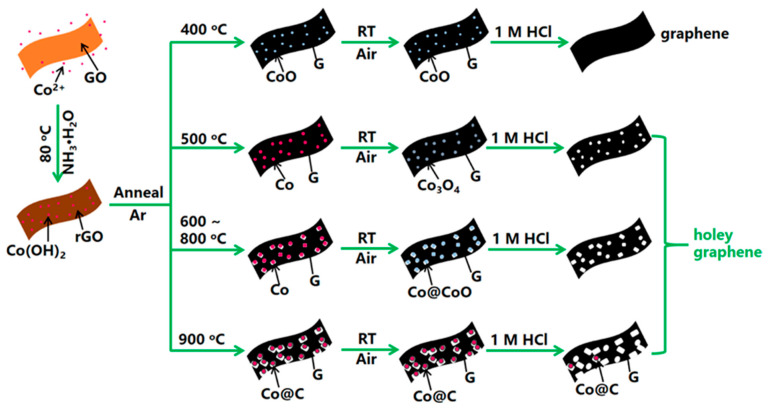
Schematic plot of the mechanism of formation of holey graphene. Adapted from [799]. © 2017 Elsevier Ltd. (2017).

Experimentally, it was reported that defects can also be more effective than heteroatom-doping for the ORR [494,810]; it was also possible to carry out a large scale and suitable cost synthesis of holey graphene with controllable size and distribution of pores in the basal plane correlating the defects density, the conductivity, and the ORR performance of the holey graphene [799]. Such holey graphene (Figure 51) was successfully and controllably prepared by carbothermal reaction of immobilized CoO_x_ with graphene in an Ar atmosphere.

The content of edge or defective carbon atom, that is the defect density, could be controlled by regulating the size and the density of holes based on different loading of Co precursor on graphene. The holey graphene with the highest defect density exhibited excellent ORR performance, with the highest ORR activity, extraordinary stability, and good methanol tolerance (Table 11).

On the other hand, because of the role of edge sites in the ORR mechanism [811] in the literature, it is well known that the importance of significantly increasing the edge sites on the basal plane is also crucial in order to overcome the ORR inertness of the basal plane [796]. This can be accomplished by reducing the size of graphene to achieve graphene nanostructures [506,795,804] mainly by mechanically reducing the dimension of graphite. Benson et al. [506] produced few layernanosized graphene with low oxygen content, with lateral dimensions smaller than a few hundred nanometers, via ionic liquid-assisted grinding of high-purity graphite and sequential centrifugation. Defect formation on the crystalline plane of graphene, or chemical reactions due to mechanochemical effects, are avoided, resulting in high-quality material with a plethora of edges. Benson et al. [506] showed that the size and thickness affect the catalytic activity of graphene nanosheets. The enhancement of the ORR performance of nanosized graphene is attributed to the abundance of edge sites accrued from the small lateral size and the efficient electron transfer between the active edge sites and the electrode.

Other graphenes with plenty of edges were obtained via, heat treatment [801], plasma [797,802] and PECVD [803]. San Roman et al. [803] produced a novel graphene-based hybrid nanomaterial, nanowire-templated out-of-plane three-dimensional fuzzy graphene, on which they exerted precise control over the size and density of out-of-plane graphene flakes and edges by varying the temperature of PECVD. In this way, they could get a tunable ORR activity as a function of graphene edge site density. The hierarchical structure allows for many exposed single-layer graphene edges that readily become oxygenated in situ under alkaline ORR conditions. Their combined experiment–theory approach suggests that edge sites are saturated by carbonyl (C=O) or hydroxyl (C-OH) groups under reaction conditions. The zigzag edge sites with high carbonyl group coverage, as well as other edge configurations with a similar local coordination environment, are predicted to enable selective, two-electron ORR.

Carbon materials possessing oxygen species are known to favor the 2e- process in ORR [677], but the 4e- process is also concomitant [791,792,796,798,799,802,803,812,813,814,815,816,817] and very recently Nagappan et al. [817] showed that a near-direct four-electron transfer reaction occurred with undoped graphene samples prepared via thermal annealing of GO. Substantial investigations have demonstrated that the 2e- ORR process is mainly determined by geometrical and electronic structures of carbon nanostructures because the change in geometrical and electronic structures affects the binding strength of the OOH intermediate and the breaking of the O-O bond [469,818,819,820].

Among the parameters directing the ORR reaction, the catalyst loading on the rotating ring-disk electrode has an important impact on activity and selectivity towards ORR in alkaline medium as evidenced by Zhang et al. [814] who investigated four representative catalysts, including graphene, exhibiting different behaviors for ORR with a 4e− pathway, 2e− pathway, or a series ‘2e− + 2e−’ pathway (Table 11).

The results confirmed that the catalyst loading influences the activity and the selectivity of the catalysts. Lower loadings favored the H_2_O_2_ electrogeneration, and therefore, for a new catalyst, it is recommended to study the loading effect in order to fully understand its electrochemical properties. The optimum loading range allows the full coverage of the electrode substrate, preventing at the same time the formation of a too-thick catalyst layer in order to maximize the catalyst utilization and to avoid the underestimation of the electrogenerated H_2_O_2_.

The presence of particular carbon oxygen groups in the catalyst is important to address the ORR activity as reported by Zhang et. al. [815]; 3D graphene materials were prepared via thermal treatment of GO, showing comparable ORR catalytic activity, better tolerance to methanol crossover effect as well as higher stability than those of commercial Pt/C. It was found, for the first time, that the C=O bonds on 3D graphene display a vital role in catalytic kinetics toward ORR, as subsequently observed also by Vasiliev et al. [624]; the C=O bonds might influence the adsorption type between the oxygen molecule and the catalytically active sites on the graphene surface. The remarkable results of a 3D graphene catalyst heated at 600 °C originate from the synergetic effect between broken C=O bonds on 3D graphene and the unique porous structure of 3D graphene. Actually, the unique porous structure of 3D graphene materials is able to trap oxygen molecules, contributing to a decrease in diffusion resistance while enhancing electrolyte-electrode accessibility for fast mass transport.

**Figure 52:**
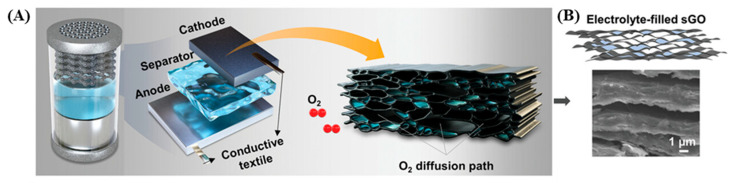
(**A**) Schematic of the structure of an aluminum–air battery. Structure of a layered spark-reduced graphene oxide (sGO) cathode in the inset. (**B**) SEM images of electrolyte-filled sGO [821] © 2021 Wiley GmbH.

Furthermore the adsorption of gaseous oxygen molecules at the liquid–solid phase boundary and the formation of a high-density gas–liquid–solid triple-phase boundary is important for accelerating the ORR as observed in aluminum–air batteries constructed using cathode based on sparked reduced graphene oxide possessing hierarchical pores [821] (Figure 52); moreover, hydrophilically treated graphene was found to be a much better material to enhance the energy production and performance of the dual-chamber MFC system [822].

## 11. Graphene Composites

As an alternative to heteroatom doping, another way to achieve active ORR electrocatalysts where the basal plane of graphene is preserved is the formation of a composite by combining graphene with carbons (doped and undoped), silicon, boron nitride, carbon nanotubes, fibers, carbon dots, electroactive molecules, and polymers.

In the literature, most composites contain graphene and carbons, and apart from a few articles dealing with composites with activated carbons [823,824], silicon nanosheets [825], coaxial cable-like electrocatalyst based on carbon fibers [826], nanodiamonds [827], fullerene [828], and with phosphorus or boron single-doped carbons [829,830], carbons always contain nitrogen; it is well known that nitrogen doping within a graphitic/turbostratic network of carbon atoms generates active sites for the ORR via C-N bond polarization, which induces a reduced energy barrier towards the ORR on the adjacent carbon atom [831]. About the chemical nature of such nitrogen-doped species, they are mostly reported as generic nitride or simply N-doped carbons, with more attention usually paid to the porosity requirements of the material [831,832,833,834,835,836,837,838,839,840,841,842,843,844,845,846,847,848].

**Scheme 7:**
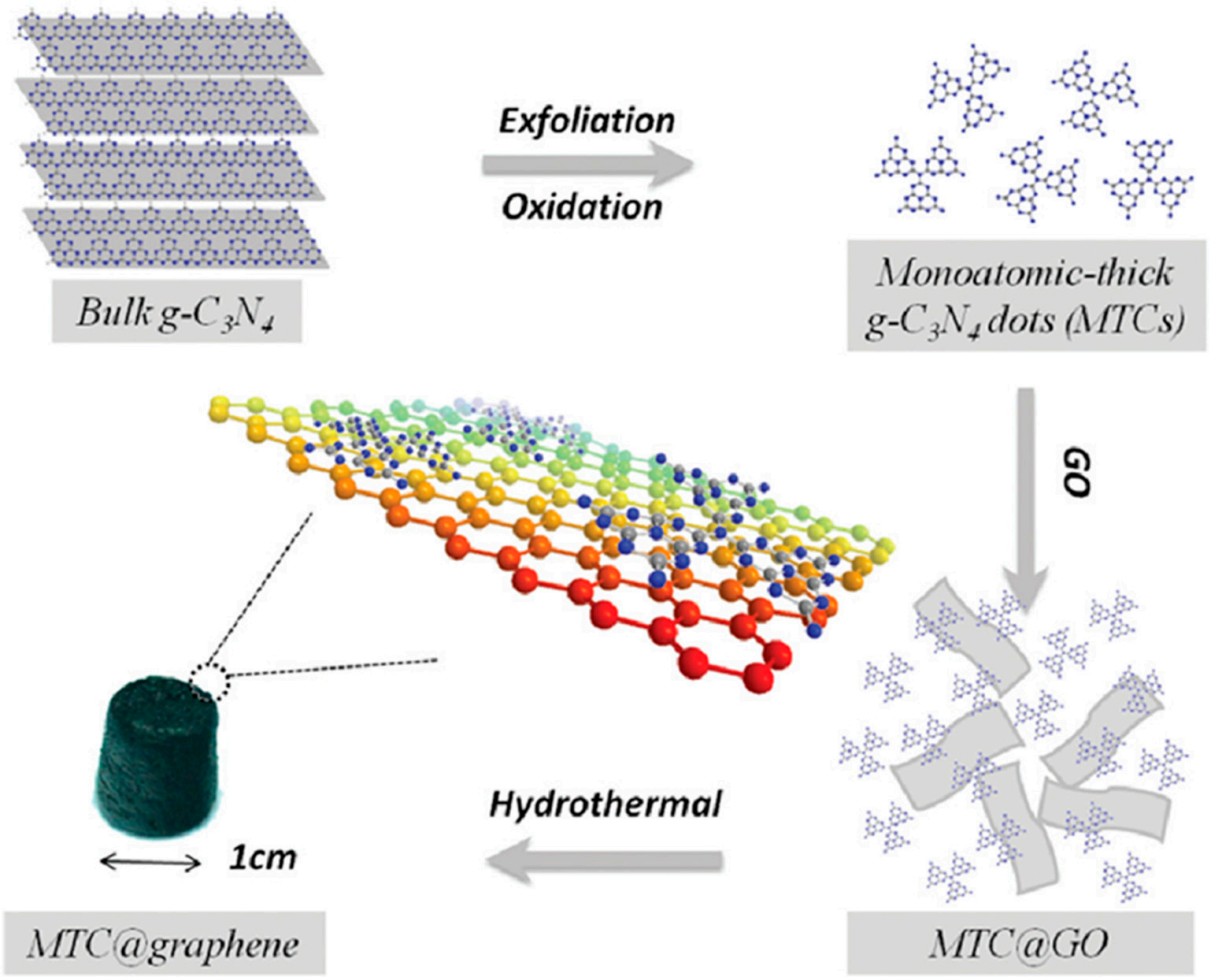
Proposed synthetic protocol for the monoatomic-thick g-C_3_N_4_ dots@graphene (MTCG). Adapted from [856] with permission from the Royal Society of Chemistry. https://pubs.rsc.org/en/content/articlelanding/2015/nr/c4nr05343e (accessed on 1 March 2025).

However, excluding the research by Kim et al. [849], who developed specific N-rich mesoporous carbon nitride with C_3_N_5_ stoichiometric and studied its graphene hybrids, in the case of graphitic carbon nitride, g-C_3_N_4_, we observe a more defined study. Graphitic carbon nitride is a promising catalyst for ORR. It is easily accessible but has a low specific surface area and low conductivity, which strongly restricts the electron transportation during the ORR process and also reduces its electrocatalytic activity. Therefore, the properties most directly related to ORR performance are thought to be manipulated by modulating the electronic structures of g-C_3_N_4_ with 2D materials [850]. There has been some effort to immobilize it onto graphene [851,852,853,854,855,856,857,858,859], which has a similar structure and relevant conductivity, since the first composite was reported by Sun et al. in 2010 [851]. Wang et al. [856] prepared monoatomic thick g-C_3_N_4_ dots (MTCs) by oxidative exfoliation of bulk g-C_3_N_4_ and put in an intimate contact to the basal plane of the graphene sheet to form the monolayer g-C_3_N_4_ dots on graphene (MTCG) through hydrothermal self-assembly (Scheme 7).

The face-to-face contact between g-C_3_N_4_ and graphene is believed to induce a synergistic coupling interaction in facilitating the ORR and electron transport through the composite with an electrocatalytic activity rivaling that of the commercial Pt/C catalyst (Table 12). Theoretical calculations and RRDE measurements indicate that the composite displayed a higher efficiency for the reduction of OOH− than the g-C_3_N_4_ alone, resulting in an enhanced performance in the ORR with an efficient four-electron pathway.

Very recently, Mane et al. [858] developed a rapid, straightforward and cost-effective synthesis technique for preparing a composite of g-C_3_N_4_ and few-layered graphene sheets; solar radiation was used to reduce GO, which forms its composite with g-C_3_N_4_ in a highly crumpled, porous, and wrinkled 2D/2D morphology with enriched Lewis base sites as active centers. This material catalyzes the ORR activity through a four-electron process, demonstrating high activity, stability, and durability, even in a methanol atmosphere. The superior performance of the catalyst is attributed to its structural characteristics and the relationship between these features and electrocatalytic activity, particularly Lewis bases as active centers.

A few more studies have been published on attempts to utilize single and multi-doped carbon dots in ORR electrocatalysis by constructing composites with graphene [623,829,860,861,862,863].

Anchoring onto rGO sheets N-doped C_dot_, S-doped C_dot,_ and N,S-co-doped C_dot_ prepared through similar hydrothermal reactions [864], Zhang et al. [623] formed composites which outperformed their rGO counterparts in terms of electrochemical properties in ORR. N,S-co-doping is a more effective strategy for realizing a four-electron transfer pathway in ORR due to the synergistic effects of N,S-co-doping. As a result, optimal N,S-C_Dot_/rGO samples exhibit the most positive half-wave potential, the highest kinetic current density, and the largest electron transfer number. With respect to substrate-constructing, active sites on C_dot_ surfaces exhibit better ORR activities than those in rGO planes, because the former are located in abundant edges/defects and have more access to oxygen molecules.

**Scheme 8:**
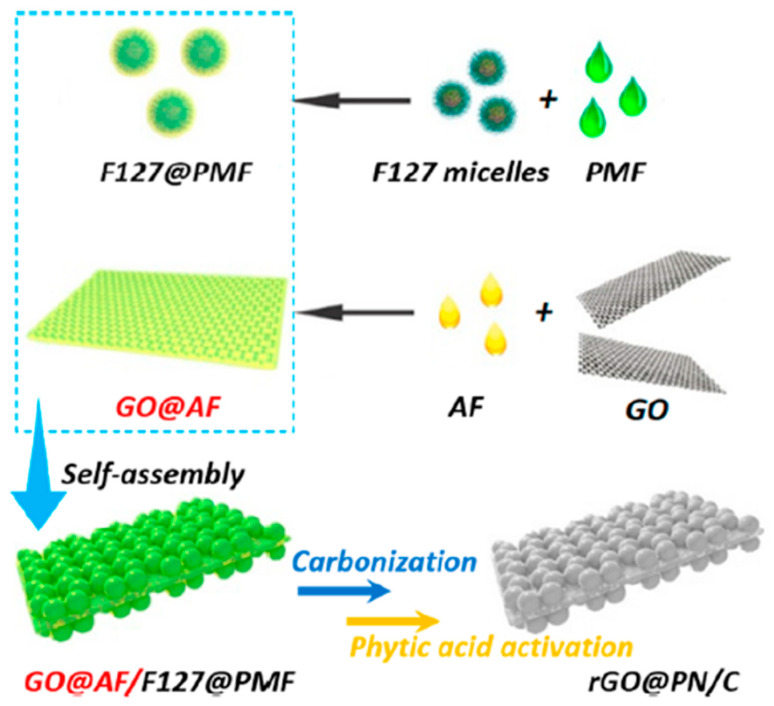
Illustration of the synthetic processes for nitrogen-doped carbon rGO@N/C and phosphorus- and nitrogen-doped carbon (rGO@PN/C). Reprinted with permission from [870]. Copyright {2020} American Chemical Society.

There are a few more examples of multi-doped carbon/graphene composites where the beneficial synergistic effect on ORR electrocatalytic performance is evident [834,865,866,867,868,869,870]. Tan et al. [870] reported 2D sandwich-like mesoporous phosphorus- and nitrogen-doped carbon nanosheets (rGO@PN/C) as a metal-free electrocatalyst for ORR (Table 12) by using graphene oxide as the substrate, triblock copolymer F127 micelles as the soft template, and resin and phytic acid as the organic precursor (Scheme 8).

The carbonization of the pre-prepared GO/F127/resin (from m-aminophenol and formaldehyde) precursor creates a 2D sandwich-structured nanoarchitecture composed of an inner layer of reduced graphene oxide and an outer layer of mesoporous nitrogen-doped carbon (rGO@N/C). The products consist of abundant mesopores formed by the assembly of F127/resin micelles, as well as the removal of F127 micelles. After activation of rGO@N/C with phytic acid (PA), phosphorus- and nitrogen-doped carbon (rGO@PN/C) nanosheets with well-maintained bimodal pores are obtained. PA can activate the carbon matrix during heat treatment under high temperatures, leading to increased porosity in carbon materials. Moreover, the incorporation of P determined considerable improvement in catalytic activity and mass transfer for the ORR. The introduction of P generates new active sites for the ORR, and the increased micro- and mesoporosity via activation with PA further facilitates mass transfer during the ORR.

In addition, the rGO@PN/C nanosheets show better durability and high selectivity compared to the commercial Pt/C catalyst.

There has been significant interest in developing 2D sandwich-like carbon nanostructures embedding graphene with ORR electrocatalytic activity [831,832,834,838,841], since the first one was reported and synthesized by Yang et al. [852] via a nanocasting method.

Liu et al. [838], following a rGO-templated 4,40-dicyanobiphenyl self-polymerization process, were able to construct a sandwich-like pyridinic N-enriched covalent triazine-based framework/rGO hybrid, which subsequently affords a sheet-like N-doped and hierarchically porous carbon/rGO composite obtained at 950 °C with dominant pyridinic and graphitic N incorporation, as a highly efficient ORR catalyst (Table 12). Thanks to the synergistic effect between the N-doped carbons and 2D rGO in terms of high surface area, hierarchical pores, controllable incorporation of active N species, favorable mass transfer, and high conductivity, the layered composite exhibits superb ORR activity and ultrahigh stability as well as excellent methanol tolerance.

It is interesting to observe that the composites of graphene with doped carbon are synthesized at high temperatures, resulting in high energy consumption, and several syntheses start from graphene polymer composites. On the other hand, composites of graphene with suitable polymers can themselves be electrocatalysts for ORR, and they can also be synthesized under mild conditions.

In the literature, composites are formed with poly(diallyldimethylammonium chloride) (PDDA) [871,872,873], polyethyleneimine (PEI) [874], amino-substituted sulfonic acid [875], polydopamine [876], adenine [877], but most with conductive polymers such as poly(3,4-ethylenedioxythiophene) (PEDOT) [878] and linear conjugated polymers based on covalently linked pyridine and thiophene [879], Nile blue [880], and PANI [881,882].

It is interesting to underline that the heteroatoms are not in the graphene carbon lattice, but in the polymers.

Barrios Cossio [881] et al. reported unique additive-free bifunctional electrodes composed of graphene oxide, reduced graphene oxide, PANI, and poly(vinyl alcohol) (PVA) on carbon cloths, which were fabricated using an easy and innovative in situ aniline polymerization strategy under mild conditions. The electrostatic interactions at GO/PANI interfaces controlled both the PANI distribution pattern and the electrical conductivity of the resulting nanohybrids, while PVA increased the ion accessibility. The GO/PANI/PVA nanocomposites showed improved conductivity properties, as well as rather uniformly distributed PANI networks, which have been directly associated with the unique synergistic interactions at the PANI/GO interfaces between the N-rich polymeric structure and the functional groups at the GO surfaces. The resulting electrodes were characterized by pH-universal oxygen reduction reaction properties, with an outstanding ORR onset potential value of 0.93 V vs. reversible hydrogen electrode in basic media. Additionally, the nanocomposite showed remarkable stability in acid media for the electroreduction of oxygen, maintaining 98% of the initial current applied after 25,000 s.

The use of polymers to impart better electrochemical properties has also been exploited by Lee et al. [873], who combined PDDA-functionalized hydrophobic multiwalled nanotubes (pMWNTs) with hydrophilic rGO nanosheets having a large surface area for the construction of metal-free carbon electrocatalysts to allow effective oxygen access in both gas and aqueous forms. They obtained 3D interconnected network structures of pMWNTs with interdispersed rGO nanosheets, driven from electrostatic stacking between oppositely charged carbon nanomaterials, which exhibited a remarkably enhanced ORR activity without extreme conditions for synthesis.

While pMWNTs provided the majority of the catalytically active sites due to their hydrophobic nature and the induced partial positive charge on their surface, introduction of rGO into their hybrid materials also improves the utility of both dissolved and gaseous oxygen and facilitates electron transfer during ORR due to its hydrophilic nature.

In this unique architecture, both two- and three-phase reactions in ORR can be maximized with a quasi-four-electron pathway.

Although composites of C_nT_ with graphene have already been illustrated in the previous paragraphs, a few are missing [883,884,885,886,887].

In particular, Cai et al. [884] prepared free-standing, N-doped vertically aligned carbon nanotubes (N-VA-CNTs) supported by graphene foam (GF), whose well-aligned structure not only works as a highly efficient electrocatalyst but also provides appropriate channels/pores for intermediate ions and gas diffusion. Due to their highly efficient catalytic activity and structural merits such as being lightweight and flexible, and possessing high conductivity, N-doped VACNTs/GF as integrated air-cathodes show significantly improved performances and current- and power-densities. A rechargeable Zn–air battery assembled from this N-VA-CNTs/GF hybrid electrode exhibits much higher power density than that of commercial Pt/C electrocatalysts and long-term durability.

To complete the overview, composites of graphene with boron nitride [888] and electroactive molecules such as antraquinone [889], tetracyanoethylene [890], and melanin [891] showed appreciable activity as ORR catalysts. In particular, Wu et al. [891] developed melanin-modified carboxylated graphene as a novel cathode catalyst in a microbial fuel cell with significantly increased bioelectricity production; this research showed the promising application of melanin in enhancing the ORR kinetics and improving microbial electron transfer in bio-electrochemical systems with interesting prospects for future sustainable and environmentally friendly applications.

## 12. Conclusions

This review offers a very wide overview of the research in the field of the ORR metal-free graphene-based ECs, paying particular attention to the synthetic strategy to improve the specific properties, determining the electroactivity of a species, which is one of the most crucial factors to be considered to fully utilize the structure of graphene and enhance its application value. The synthesis should be developed through an easily scalable protocol to be compatible with large-scale industrial production, and even in greener and more sustainable ways to meet ecological requirements.

Most of both theoretical and experimental studies on ORR metal-free graphene-based ECs (above 600 papers) which show how the electrocatalytic properties and the ORR performance of graphene-based catalysts strongly depend on their composition, including the type and number of doping elements, the materials’ structure and morphology, such as size and shape, as well as electronic effects, have been reported; this should help the scientist in addressing the research towards the design of a suitable electrocatalyst even if its activity cannot be easily predicted.

This review indicates that the first strategy, which can be suggested for a good electrocatalyst, is the introduction of heteroatoms into the carbon structure to bring about modifications to both its chemical and physical properties.

Nitrogen-doping has been largely investigated, resulting in active ECs; however, there are other dopants with suitable physicochemical properties, such as B, P, and S, that may be included in the carbon core. Future development would involve investigating the usage of other heteroatoms, such as selenium, which proved to be promising but was not developed further. It may also be beneficial to start from natural products that contain it, acting as green, single-source precursor. It can also be considered that the creation of desirable active motifs with distinctive coordination structures and electronic states that are beneficial for improved electrochemical performance could be guided by computational explorations such as artificial-intelligence-assisted material screening or DFT predictions with machine learning.

Moreover, it is still a challenge to control the number of heteroatoms, their distributional homogeneity, bonding forms, and other aspects, which are particularly evident in co-doped species. The position and number of heteroatoms in the carbon basal plane are still difficult to accurately adjust as well as the establishment of a clear connection between both the doped structure and catalytic performance deserving further experimental studies, state-of-the-art characterizations, and robust computational simulations to a deeper understanding of the structure, mechanism, and thermodynamics of the catalytic core.

In particular, multi-doping, creating sites simultaneously carrying positive charge and high spin density, is also considered a promising way to achieve the goal of enhancing ORR activity in acidic electrolytes in order to potentially or effectively substitute, in particular, platinum. The metal-free graphene ORR ECs offer the natural advantages of durability in acid due to the absence of metal leaching, metal-ion contamination, and Fenton reaction–related degradation but unfortunately, the best ORR performances are generally obtained under alkaline conditions, even if with appreciable durability.

Beyond the selection of elements, the catalytic performance, which is definitely related to the intrinsic activity, enrichment degree, and accessibility of the active sites, can be improved by the design of graphene structures via morphological and vacancy engineering of catalysts and interface effects.

Catalysts with 3D structures, such as hollow structures, core-shell structures, etc., usually have high mass transfer efficiency. In particular, the reaction kinetics of ORR can be effectively promoted in 3D interconnected layered porous structures and high specific surface areas: additional active sites can be provided and facilitate the adsorption and desorption of intermediates because of efficient charge/material transport. Therefore, porosity is very important, even if what better defines the catalytic activity is the electrochemically active surface area.

Considering vacancy engineering of catalysts and interface effects, it is important to control the vacancy defects and edges, which can induce charge redistribution, change the local electronic structure, but without shattering the conductivity, and effectively improve intrinsic catalytic activity. Interface engineering is also one of the effective strategies for improving catalytic activity. The interface formed between the components in the electrocatalyst can effectively change the electronic structure and atomic configuration of the active site.

It has to be highlighted that most of the existing catalysts were tested in the laboratory, and, therefore, it is necessary to convert laboratory techniques into practical results to achieve high efficiency and stability in practical environments. A lot of effort must therefore be made to fill the gap between “simple” RDE or RRDE measurements and, for example, tests in real MEA.

To conclude, the author must warn that what are largely claimed to be metal-free electrocatalysts often lack proper, substantial elemental analysis. This can be achieved, for example, via inductively coupled plasma, which should evidence the absence of metals; metals derived, for instance, from starting reagents, even in trace amounts, can act as effective ORR electrocatalytic species. The presence of metal impurities is a current issue in catalysis; however, this observation does not negate that graphene is a promising material for ORR electrocatalysis.

**Table 2:** Comparative analysis of some ORR parameters of the synthesized N-doped graphene-based ECs ^1^ with best performance in alkaline electrolytes.

Catalyst ^2^		Catalyst Loading ^3^ (μgcm^−2^)	E_onset_ vs. RHE ^4^ (V)	Electrolyte pH	Synthetic Method ^5^	Ref.
*N-GQDs_3* N-doped graphene quantum dots	**NG_qDor_**	283.08	0.610	13	electrochemical method	[316]
*N-rGO* N-doped reduced graphene oxide	**N-rGO**	121.43	0.620	13	annealing	[561]
*N/3D-GNS 850* three-dimensional N-doped graphene nanosheets	**NG_3DnSh_ 850** **NG_3DnSh_ 850**	200 200	0.630 0.870	13 1 ^6^	thermal heating	[400]
*TDMAC-RGO* Tridodecylmethylammonium chloride (TDMAC)-functionalized reduced graphene oxide	**TDMAC-RGO** TDMAC = Tridodecylmethylammonium chloride	50.96	0.650	13	solution process	[428]
*Nr-GO4* N-doped reduced graphene oxide	**N-rGO (III)**	25	0.650	13	annealing	[516]
*GF* N-doped 3D graphene framework	**NG_3Dχ_**	12	0.670	13	hydrothermal process	[521]
*NG60* nitrogen-doped graphene	**NG (II)**	1187.31	0.680	13	sonochemical process	[436]
*NB-graphene* Nitrobenzene-doped graphene	**Nitrobenzene-G**	151.65	0.700	13	solution process	[427]
*1F-800* N-doped graphene foam	**NG_Fo_ 800**	400	0.700	13	carbonization	[442]
*N-rGO (E)* N-doped reduced graphene oxide	**N-rGO (II)**	15	0.710	13	hydrothermal process	[570]
*NG−1* N-doped graphene	**NG(I)**	40	0.710	13	pyrolysis	[421]
*NGQDs/G* N-doped graphene quantum dots supported by graphene	**NG_qDot_/G**	283.08	0.710	13	hydrothermal process	[283]
*run 7* N-doped reduced graphene oxide	**N-rGO (II)**	1000	0.710	13	microwave irradiation	[417]
*N-dGOA* N-doped graphene oxide aerogel (GOA)	**NGO_Ae_**	27.75	0.720	13	solution process	[529]
*NG/CCN light* N-doped graphene/carbon-rich C_3_N_4_ composite	**NG/C_3_N_4_**	500	0.730	13	calcination	[439]
*PyNG-3* Pyridine-nitrogen-doped graphene sheets	**Pyridine-G_sh_ (II)**	141.54	0.738	13	solution process	[358]
*N-GQDs/G-12* N-doped graphene quantum dots/graphene hybrid	**NG_qDot_/G (I)**	70.77	0.740	13	hydrothermal process	[319]
*N-Gr* nitrogen-doped graphene	**NG** **NG** **NG**	81.53 81.53 81.53	0.740 0.440 0.210	13 7 ^6^ 0.3 ^6^	electrochemical process	[558]
*N-FLG* Nm-FLG8 nitrogen-rich few-layered graphene	**NG_fl_ (III)**	101.21	0.740	13	annealing	[418]
*NGSH* Nitrogen-doped graphene/carbon nanotube hybrids	**NC_swnT_/NG**	254.77	0.740	13	CVD	[541]
*NG* N-doped graphene	**NG**	141	0.744	13	pyrolysis	[381]
*NHG* Nitrogen-doped holey graphene	**NG_hl_**	25	0.750	13	hydrothermal process	[490]
*N-RGO3* N-doped reduced graphene oxide	**N-rGO (III)**	70.77	0.750	13	annealing	[310]
*G N-max* N-doped graphene	**NG (I)**	150	0.750	13	thermal treatment	[473]
*HPN-MQGs* high-power N-doped medium-quality graphene sheets	**NG_Sh_ (III)**	200	0.750	13	microwave plasma torch	[318]
*HT-N-RGO* N-doped reduced graphene oxide	**N-rGO**	600	0.750	13	hydrothermal process	[472]
*N8,3-NGR* Nitrogen-doped graphene nanoribbon	**NG_nR_ (IV)**	141.54	0.765	13	pyrolysis	[456]
*NG* N-doped graphene	**NG**	142.86	0.765	13	thermal annealing	[380]
*HTNG/GCE* N-doped graphene	**NG (II)**	79.62	0.769	13	thermal annealing	[383]
*N-rGO-90* N-doped reduced graphene oxide	**N-rGO 90**	2000	0.770	13	solution process	[443]
*N-G 900* N-doped graphene	**NG**	1166.86	0.778	13	annealing	[462]
*NG10* N-doped graphene	**NG**	20.30	0.780	13	thermal annealing	[437]
*NGnP* N-doped graphene nanoplatelets	**NG_nPl_**	76.43	0.780	13	ball milling	[505]
*NG-2/GC* N-doped graphene	**NG (II)**	137.62	0.780	13	hydrothermal process	[504]
*N-RGO10* 3-D mesoporous N-doped reduced graphene oxide	**N-rGO_3Dmpo_ (II)**	160	0.780	13	annealing	[317]
*N/G1050* N-doped graphene	**NG 1050**	128	0.785	13	annealing	[323]
*rGO-sp3-rGO*	**Diaminobutano-rGO (II)**	51.02	0.790	13	solution process	[425]
*NG-PPy* N-doped graphene	**NG_poL_**	500	0.790	13	carbonization	[407]
*NG* nitrogen-doped graphene	**NG**	243	0.795	13	hydrothermal method	[457]
*N-rGO900* N-doped reduced graphene oxide	**N-rGO**	141.54	0.798	13	thermal annealing	[557]
*NG-NCNT* Nitrogen-doped graphene/carbon nanotube nanocomposite	**NC_nT_/NG**	50.96	0.799	13	hydrothermal process	[538]
*NG 900* N-doped graphene	**NG 900**	141	0.800	13	pyrolysis	[384]
*N-doped MSMG-P* N-doped mechanochemically synthesized multilayer graphene	**NG_mL_**	1530	0.800	13	mechanochemical synthesis	[502]
*rGO N2H4* N-doped reduced graphene oxide	**N-rGO**	424	0.800	13	hydrothermal process	[120]
*NMGF* nitrogen-doped mesoporous graphene framework	**NG_mpoχ_**	255.10	0.801	13	CVD	[497]
*N-GP 4-hr 600 °C* N-doped graphene nanoplatelets	**NG_nPl_ 600 (II)**	424	0.805	13	pyrolysis	[446]
*N-G* N-doped graphene	**NG**	200	0.808	13	annealing	[128]
*G-NH3* H2O N-doped graphene	**NG (II)**	204.08	0.810	13	annealing	[313]
*GO/NH3·H2O* oxo-G-derived nitrogen-doped graphene	**N-rGO (III)**	100	0.810	13	hydrothermal treatment	[499]
*PB-N-rGO* 1-pyrenebutyrate functionalized N-doped graphene	**1-pyrenebutyrate-rGO**	170	0.810	13	solution process	[320]
						
*N5-rGO* N-doped reduced graphene oxide	**N-rGO**	510	0.810	13	hydrothermal process	[476]
*A-rGO* ammonia-reduced graphene oxide	**rGON**	597.13	0.810	13	Solution process	[568]
*N-pGF* pyridinic-N-doped graphene film	**NG_Fm_ (II)** **NG_Fm_ (II)**	50 50	0.815 0.211	13 0.3 ^6^	solution process	[333]
*NG-750* N-doped graphene	**NG 750**	61.22	0.815	13	thermal treatment	[392]
*NG 1* nitrogen-doped reduced graphene oxides	**N-rGO (I)**	103	0.815	13	annealing	[395]
*NGR^−1^000* nitrogen-doped graphene	**NG 1000**	140	0.815	13	pyrolysis	[445]
*IRnG-A2* A2 imine-rich nitrogen-doped graphene nanosheets	**[(CF_3_SO_2_)C_6_H_4_N)]-G**	200	0.815	13	solution process	[429]
*Graphene:glycine 1:4* N-doped graphene	**NG (II)**	509.55	0.815	13	pyrolysis	[388]
*NHG* nitrogen-doped holey graphene	**NG_hl_**	10	0.819	13	ball milling	[491]
*T 1000* N-doped graphene-wrapped carbon nanoparticles	**NG 1000/NC_nP_**	152.87	0.819	13	solvothermal process	[459]
*Lem-rGO (_8)* N-functionalized rGO	**N-rGO (I)**	51.02	0.820	13	solution process	[533]
*NG 1* N-doped graphene	**NG**	103	0.820	13	thermal annealing	[398]
*N-rGO 800* N-doped reduced graphene oxide	**N-rGO (IV)**	203.11	0.820	13	annealing	[328]
*N-rGO^-^180* N-doped reduced graphene oxide	**N-rGO (IV)**	492.96	0.820	13	microwave treatment	[335]
*GD1* N-doped graphene	**NG (I)**	509.55	0.820	13	solution process	[487]
*CS@N-G/CNT* carbon spheres@nitrogen-doped graphene/carbon nanotubes hybrid	**C_Sp_/NG/C_nT_**	570	0.825	13	ultrasonic-assisted process	[545]
*NG 900* N-doped graphene	**NG 900**	200	0.828	13	calcination	[338]
*N-RGO* N-doped graphene	**N-rGO**	190	0.829	13	annealing	[489]
*1000-O2* N-doped graphene	**NG (II)**	120	0.830	13	annealing	[494]
*NG* N-doped graphene	**NG**	194	0.830	13	microwave plasma	[438]
*GC900* three-dimensional N-doped graphene	**NG_3D_ 900**	200	0.830	13	pyrolysis	[405]
*N-rGO–CNT-0.2* N-doped rGO–carbon nanotube composites	**N-rGO/NC_nT_ (II)**	600	0.830	13	annealing	[542]
*NG* N-doped graphene	**NG**	39.81	0.835	13	annealing	[127]
*N-graphene* N-doped reduced graphene oxide	**N-rGO**	160	0.835	13	shock synthesis of graphene material from CO_2_	[559]
*NCDs-NG^−1^2* N-doped reduced graphene oxide	**NC_Dot_/NG (II)**	79.22	0.840	13	hydrothermal process	[326]
*3-NG* N-doped graphene	**NG (III)**	100	0.840	13	pyrolysis	[311]
*N2-3DrGO* N-doped three-dimensional reduced graphene oxide	**N-rGO_3D_ (II)**	101.91	0.840	13	calcination	[464]
*N-OMMC-G* N-doped graphene natively grown on hierarchical ordered porous carbon	**NG/CN_mamop_**	416.67	0.840	13	heating and silica etching	[522]
*N-rGO 800* N-doped reduced graphene oxide	**N-rGO 800**	1100	0.840	13	pyrolysis	[564]
*NDG* N-doped graphene	**NG**	51	0.845	13	annealing	[435]
*CN1000* N-doped graphene	**NG 1000** **NG 1000**	144 144	0.847 0.567	14 7 ^6^	thermal treatment	[390]
*NDTG* N-doped double-layer templated graphene	**NG (I)**	254.8	0.849	13	CVD	[534]
*NG^−1^000* nitrogen-doped graphene	**NG 1000 (I)**	97.99	0.850	13	annealing	[386]
*CHs material* N-doped graphene	**NG_poL_**	106.16	0.850	13	carbonization	[449]
*GN-CNT 2* N-rich graphene nanoclusters–carbon nanotube composite	**NC_nT_/NG_nCl_**	106.16	0.850	13	deflagration with NaNO_3_	[477]
*PM-NGr/NCNT* N-doped graphene/carbon nanotube	**NG/NC_nT_ (I)**	106.16	0.850	13	pyrolysis	[548]
*NGM* N-doped graphene mesh	**NG_mh_** **NG_mh_**	254.78 254.78	0.850 0.540	13 1 ^6^	carbonization	[496]
*DNGS 480 900* dendritic N-doped graphene spheres	**NG_ddSp_ (I)** **NG_ddSp_ (I)** **NG_ddSp_ (I)**	1000 1000 1000	0.770 0.550 0.470	13 0.3 ^6^ 7 ^6^	pyrolysis	[532]
*NG-SCCf* N-doped graphene grown on carbon fibers derived from silk cocoon (SCCf)	**NG/NC_Fb_**	320	0.850	13	thermal treatment	[524]
*G800* N-doped few-layered graphene sheets	**NG_fLSh_**	400	0.850	13	carbonization	[451]
*NVG-30* N-doped vertical graphene nanosheets	**NG_vSh_**	470	0.850	13	Plasma-enhanced CVD	[351]
*HNG-900* holey N-doped graphene	**NG_hl_ 900**	200	0.860	13	thermal heating	[399]
*NGF* N-doped graphene framework	**NG_χ_**	200	0.860	13	thermal heating	[402]
*NGE 1000* nitrogen-doped graphene	**NG 1000**	225	0.860	13	heat treatment	[416]
*N-Gr* N-doped graphene	**NG**	420	0.860	13	thermal treatment	[408]
*NG-30* N-doped graphene	**NG (IV)**	485	0.868	13	microwave heating	[389]
*NG/NCNT-BR* N-doped graphene from biuret	**NG/NC_nT_ (III)**	100	0.869	13	pyrolysis	[321]
*N-MG-800* 3D bicontinuous N-doped mesoporous graphene	**NG_3Dmpo_ 800**	100	0.870	13	thermal heating	[336]
*NGA^−1^50* N-doped graphene aerogels	**NG_Ae_ (III)**	102	0.870	13	pyrolysis	[469]
*N-graphene 900* N-doped graphene	**NG (I)**	160	0.870	13	thermal treatment	[378]
*ENG* N-doped graphene	**NG (II)**	200	0.870	13	electrochemical exfoliation	[515]
*CN 900* N-doped graphene	**NG 900**	280	0.870	13	carbonization	[556]
*N-GNRs-A* N-doped graphene nanoribbons aerogel	**NG_hl_ 900**	80	0.875	13	pyrolysis	[527]
*DG* N-doped graphene annealed at 1150 °C	**NG (II)** **NG(II)**	79.62 79.62	0.880 0.530	13 1 ^6^	annealing	[810]
*N-GNR* nitrogen-doped graphene nanoribbon	**NG_nR_**	175.07	0.880	13	solution process	[325]
*N-RGO-PPV(c)-CNTs* three-dimensional N-doped carbon nanotube/reduced graphene oxide composite	**NC_nT_/N-rGO**	300.57	0.880	13	pyrolysis	[546]
*N-rGO Diethyl ether* N-doped reduced graphene oxide	**N-rGO (III)**	600	0.880	13	annealing	[498]
*N-GRW* three-dimensional (3D) graphene nanoribbon networks	**NG_3DnRχ_**	600	0.880	14	pyrolysis	[396]
*N-aGS-900* N-doped activated graphene/single-wall carbon nanotube hybrid	**NG/NC_sWnT_**	177	0.885	13	pyrolysis/annealing	[547]
*NG-C* N-doped graphene	**NG (III)**	200	0.885	13	ultrasound	[315]
*ENR-GNPs* edge-nitrogen-rich graphene nanoplatelets	**NG_edrnPl_** **NG_edrnPl_**	283 283	0.885 0.584	13 0.3 ^6^	ball milling	[387]
*NHGs* nitrogen-doped hollow graphene microspheres	**NG_hwmSp_**	10	0.890	13	pyrolysis	[525]
*NMG^−1^/4* nitrogen-doped mesoporous graphene	**NG_mpo_**	141.54	0.890	13	annealing	[526]
*N-rGO/PAA-900* N-doped reduced graphene oxide	**N-rGO**	394.89	0.890	13	pyrolysis	[484]
*NOGB-800* N,O-co-doped graphene nanorings-integrated boxes	**ONG_nBx_ 800** **ONG_nBx_ 800**	400 400	0.890 0.710	13 1 ^6^	calcination	[342]
*NGA* N-doped three-dimensional porous graphene frameworks	**NG_3Dpoχ_**	101.91	0.898	13	calcination	[470]
*PHNG-800* porous holey nitrogen-doped graphene	**NG_pohl_ 800**	254.78	0.900	13	pyrolysis	[465]
*NG-2* N-doped graphene	**NG_poL_**	606.61	0.907	14	solvothermal process	[205]
*NG1000* N-doped graphene	**NG 1000**	38.22	0.908	13	thermal treatment	[454]
*N-GQD/rGO* nitrogen-doped graphene quantum dots anchored on N-doped graphene	**NG_qDot_/rGO**	100	0.910	13	calcination	[410]
*NG 1000* N-doped graphene	**NG 1000** **NG 1000**	152 152	0.910 0.750	13 1 ^6^	pyrolysis	[495]
*O,N-graphene* O,N-co-doped 3D graphene hollow sphere	**ONG_hSp_**	250	0.910	13	annealing	[479]
*DN-UGNR 3nm 900* N-doped ultranarrow graphene nanoribbons	**NG_unwnR_ (I)**	300	0.910	13	thermal treatment	[341]
*N-GN* N-doped graphene	**NG**	400	0.910	13	thermal process	[433]
*GHN-C 900* graphene hydrogel-based nitrogen carbon materials	**NC/NG 900** **NC/NG 900** **NC/NG 1000**	450 450 250	0.910 0.640 0.620	13 1 ^6^ 13	pyrolysis	[440]
*N-GPp(few layer)* N-doped graphene (few layers)	**NG (I)**	800	0.910	13	electrochemical exfoliation	[343]
*Twostep* N-doped graphene	**NG (I)**	1900	0.910	13	hydrothermal process	[401]
*NG 1000* nitrogen-doped graphene	**NG 1000**	283	0.915	13	pyrolysis	[240]
*N-rGO-P* N-doped reduced graphene oxide	**N-rGO (II)**	100	0.920	13	pyrolysis	[406]
*NGS_1000* porous N-doped graphene layers	**NG_poL_** **NG_poL_**	204.08 204.08	0.920 0.680	13 0.3 ^6^	pyrolysis	[404]
*N-hG6* holey N-doped graphene	**NG_hl_**	250	0.920	13	annealing	[352]
*NGA950* N-doped graphene after annealing at 950 °C	**NG 950**	283.09	0.920	13	pyrolysis	[458]
*3D-PNG* three-dimensional nanoporous nitrogen-doped graphene	**NG_3Dnpo_**	485	0.920	13	pyrolysis and etching	[461]
*NGA* N-doped graphene aerogel	**NG_Ae_**	101.91	0.930	13	annealing	[472]
*NG 800* N-doped graphene	**NG 800**	180	0.930	13	CVD	[304]
*N-GNRs/G* N-doped graphene nanoribbons	**NG_nR_/NG**	255	0.930	13	pyrolysis	[482]
*Ai-HGs* 3 3,4-diaminopyridine grafted N-doped holey graphene	**3,4diaminopyridine-G_hl_**	305.73	0.930	13	solution chemistry	[349]
*g-C3N4@N-G* Graphitic carbon nitrides supported by N-doped graphene	**g-C_3_N_4_/NG**	200	0.940	13	carbonization	[514]
*NDGs-800* N-dominated doped defective graphene	**NG_df_**	204	0.940	13	thermal heating	[339]
*NGNs-900* N-doped edge-rich graphene nanosphere	**NG_edrSp_**	254.78	0.940	13	pyrolysis	[483]
*N-G 1000* N-doped graphene	**NG (I)** **NG (I)**	485 485	0.940 0.800	13 1 ^6^	pyrolysis	[403]
*CrG-900* N-doped crumpled graphene	**NG_cr_ 900**	600	0.940	13	solvothermal process	[397]
*N-SMCTs@N-rGO* N-doped sub-micron carbon tubes with N-doped reduced graphene oxide	**NC_sμT_/N-rGO**	204	0.950	13	carbonization	[481]
*N-GNR@CNT* N-doped graphene nanoribbons on carbon nanotubes	**NG_nR_/NC_nT_** **NG_nR_/NC_nT_**	398 398	0.950 0.700	13 0.3 ^6^	annealing	[549]
*HPGF−1* hierarchical porous N-doped graphene foams	**NG_hipo_-_Fo_ (I)** **NG_hipo_-_Fo_ (I)**	485 485	0.950 0.780	13 1 ^6^	calcination and silica etching	[460]
*N-rGO* N-doped reduced graphene oxide	**N-rGO**	170	0.952	14	annealing	[176]
*NA-3DGFs 600* N-doped densely arranged sharp edges graphene fibers	**NG_3DFb_ (II)**	150	0.960	13	thermal treatment	[348]
*NSAOrGO* N-doped macroporous carbon materials	**NSC/rGON (I)**	159.24	0.960	13	pyrolysis	[478]
*NGS4 1000* nitrogen-doped graphene nanosheets	**N_nsha_ 1000**	407	0.960	13	annealing	[350]
*N-GN15* N-doped graphene	**NG (I)** **NG (I)**	102 102	0.980 0.850	13 1 ^6^	annealing	[393]
*NPG 1-05* N-doped graphene nanoporous graphene	**NG_po_ (I)**	350	0.980	13	pyrolysis	[330]
*NHGNs* nitrogen-doped graphene sheets	**NG_hlnCs_**	394.15	0.98	13	hydrothermal process	[448]
*NGTB-900* high-density pyridinic-N-doped graphene− nanotube complexes with hierarchical networks	**NC_nT_/NG 900** **NC_nT_/NG 900**	600 600	1.01 0.780	13 1^6^	annealing	[550]

^1^ For proper comparison, this table contains only data from samples whose catalyst loading can be defined. ^2^ The catalyst name in italic is the catalyst name reported in the paper with the best E_onset_, reported in the third column and below the explanation reported in the paper; in bold, the name is set using the terms reported in the legend following mainly the indications reported in [892]. ^3^ Catalyst loading, when not reported in the paper, has been calculated from data provided by the authors. ^4^ E_onset_ was generally extracted from the LSV curves analyzed using the WebPlot program. ^5^ The synthesis method is not fully described but usually refers to the last treatment. ^6^ For a direct comparison, values at different pH have also been reported.

**Table 3:** Comparative analysis of some ORR parameters of the synthesized N-doped graphene-based ECs ^1^ with best performance in acidic electrolytes.

Catalyst ^2^		Catalyst Loading ^3^ (μgcm^−2^)	E_onset_ vs. RHE ^4^ (V)	Electrolyte pH	Synthetic Method ^5^	Ref.
*N-Gr* N-doped graphene	**NG** **NG** **NG**	81.53 81.53 81.53	0.210 0.740 0.440	0.3 13 ^6^ 7 ^6^	electrochemical process	[558]
*N-pGF* pyridinic-N-doped graphene film	**NG_Fm_ (II)**	50 50	0.211 0.815	0.3 13 ^6^	solution process	[333]
*N-GNS 800* N-doped graphene	**NG_nSh_**	200	0.310	0.3	pyrolysis	[434]
*N-Graphene*	**NG**	100	0.420	1	annealing	[334]
*NG* N-doped graphene	**NG**	106	0.470	0.3	annealing	[345]
*DG* N-doped graphene	**NG (II)** **NG (II)**	79.62 79.62	0.530 0.880	1 13 ^6^	annealing	[810]
*NGM* N-doped graphene mesh	**NG_mh_** **NG_mh_**	254.78 254.78	0.540 0.850	1 13 ^6^	carbonization	[496]
*DNGS 480 900* dendritic N-doped graphene spheres	**NG_ddSp_/C_sp_ 900 (I)** **NG_ddSp_/C_sp_ 900 (I)** **NG_ddSp_/C_sp_ 900 (I)**	1000 1000 1000	0.550 0.770 0.470	0.3 13 ^6^ 7 ^6^	pyrolysis	[532]
nitrogen-doped graphene foam	**NG_Fo_**	580	0.570	1	solvothermal process	[492]
*ENR-GNPs* edge-nitrogen-rich graphene nanoplatelets	**NG_edrnPl_** **NG_edrnPl_**	283 283	0.584 0.885	0.3 13 ^6^	ball milling	[387]
*Nr-GO4* N-doped reduced graphene oxide	**N-rGO (III)** **N-rGO (III)**	25 25	0.580 0.660	5 1	annealing annealing	[516]
*N-MG-801* 3D bicontinuous N-doped mesoporous graphene	**NG_3Dmpo_ 800**	400	0.590	0.3	thermal heating	[336]
*N-HPC/RGO^−1^* N-doped hierarchical porous carbon reduced graphene oxide	**NC_hipo_/NrGO**	101.91	0.625	0.3	pyrolysis	[463]
*GHN-C 900* graphene hydrogel-based nitrogen/carbon materials	**NC/NG 900** **NC/NG 900**	450 450 250	0.640 0.910 0.620	1 13 ^6^ 13 ^6^	pyrolysis	[440]
*N-Gr* N-doped graphene	**NG**	707.71	0.670	1	pyrolysis	[640]
*HS-MW 1200* nanoporous N-doped reduced graphene oxide	**NrGO_npo_ (V)**	325	0.680	1	annealing	[503]
*N-GNR@CNT* N-doped graphene nanoribbons on carbon nanotubes	**NG_nR_/NC_nT_** **NG_nR_/NC_nT_**	398 398	0.700 0.950	0.3 13 ^6^	annealing	[549]
*NOGB-800* N,O-co-doped graphene nanorings-integrated boxes	**ONG_nBx_ 800** **ONG_nBx_ 800**	400 400	0.710 0.890	1 13 ^6^	calcination	[342]
*GN 1000* N-doped graphene	**NG 1000** **NG 1000**	152 152	0.750 0.910	1 13 ^6^	pyrolysis	[495]
*GCA 4* N-doped graphene–carbon nanotube self-assembly	**NC_nT_/NG (IV)**	707.71	0.760	1	thermal heating	[540]
*HPGF^−1^* hierarchical porous N-doped graphene foams	**NG_hipoFo_ (I)** **NG_hipoFo_ (I)**	485 485	0.780 0.950	1 13 ^6^	calcination and silica etching	[460]
*NGTB-900* high-density pyridinic-N-doped graphene− nanotube complexes with hierarchical networks	**NC_nT_/NG 900** **NC_nT_/NG 900**	600 600	0.780 1.010	1 13 ^6^	annealing	[550]
*PNGF* porous N-doped graphene foam	**NG_poFo_**	485	0.790	1	pyrolysis and etching	[518]
*N-G 1000* N-doped graphene	**NG (I)** **NG (I)**	485 485	0.800 0.940	1 13 ^6^	pyrolysis	[403]
*NGCA* N-doped graphene/CNT self-assembly	**NG/CN_mamop_**	714	0.810	1	pyrolysis	[539]
*CNG-3* 3D interconnected carbon nitride (CNx) tetrapods wrapped with nitrogen-doped graphene	**NG/CN_xtPod_ (II)**	255	0.820	1	annealing	[432]
*NG@MMT* N-doped graphene	**NG**	600	0.830	1	pyrolysis	[452]
*N-GN15* N-doped graphene	**NG (I)** **NG (I)**	102 102	0.850 0.980	1 13 ^6^	annealing	[393]
*N/3D-GNS 850* three-dimensional N-doped graphene nanosheets	**NG_3DnSh_ 850** **NG_3DnSh_ 850**	200 200	0.630 0.870	0.3 13 ^6^	thermal heating	[400]
*NGS_1000* porous N-doped graphene layers	**NG_poL_**	204.08 204.08	0.680 0.920	0.3 13 ^6^	pyrolysis	[404]

^1^ For a proper comparison, this table contains only data from samples whose catalyst loading can be defined. ^2^ The catalyst name in italic is the catalyst name reported in the paper with the best E_onset_, reported in the third column and below the explanation reported in the paper; in bold is the name set using the terms reported in the legend, following mostly the indications reported in [892]. ^3^ Catalyst loading, when not reported in the paper, has been calculated from data provided by the authors. ^4^ E_onset_ was generally extracted from the LSV curves analyzed via the WebPlot program. ^5^ The synthesis method is not fully described but it usually refers to the last treatment. ^6^ For a direct comparison, values at different pH have also been reported.

**Table 4:** Comparative analysis of ORR parameters of the synthesized S-doped graphene-based ECs ^1^ with best performance in alkaline electrolytes.

Catalyst ^2^		Catalyst Loading ^3^ (μgcm^−2^)	E_onset_ vs. RHE ^4^ (V)	Electrolyte pH	Synthetic Method ^5^	Ref.
*S-GNF4* N-doped graphene nanoflakes	**SG_nFk_ (I)**	100	0.610	13	thermal plasma	[605]
*c-FLGS* sulfur-containing few-layer graphene	**SG_fL_(II)**	40	0.680	13	plasma-assisted electrochemical exfoliation	[624]
*SG 900* S-doped graphene	**SG 900**	38.22	0.728	13	thermal treatment	[454]
*S-rGO* S-doped reduced graphene oxide	**S-rGO** **S-rGO**	255 255	0.740 0.170	13 1 ^5^	solution process	[615]
*SPG* S-doped porous holey graphene frameworks	**SG_pohoχ_**	125	0.745	13	pyrolysis	[595]
*SGnP* edge-selectively sulfurized graphene nanoplatelet	**GS_nPl_**	76.43	0.750	13	ball milling	[587]
*S-RGO−180* sulfur-doped reduced graphene oxide	**S-rGO 180**	50	0.760	13	hydrothermal process	[620]
*rGO H2SO4* S-doped reduced graphene oxide	**S-rGO**	424	0.760	13	hydrothermal process	[120]
*rGO_Se* selenium-doped reduced graphene oxide	**Se-rGO**	169.85	0.770	13	solution process	[629]
*SDGN(10)* S-doped graphene nanosheets	**SG_nSh_ (IV)**	476.19	0.790	13	electrochemical exfoliation	[610]
*SG-700* S-doped graphene	**SG 700**	320	0.810	13	thermal reduction	[602]
*S-doped MSMG-C* mechanochemically synthesized multilayer graphene	**SG_mL_**	1530	0.810	13	mechanochemical synthesis	[502]
*S-GNs^−^1000-CB* sulfur-doped graphene nanosheets/carbon black composite	**SG_nSh_/C_b_ 1000**	203.82	0.820	13	annealing	[599]
*rGO3TP* 2,2′:5′,2″-terthiophene-rGO	**3TP-rGO (I)** **3TP= 2,2′:5′,2**″**-terthiophene**	50.96	0.825	13	solution process	[625]
*S-GQDs/CNPs* hybrid S-doped graphene quantum-dot-decorated carbon nanoparticles	**SG_qDot_/C_nP_**	318.47	0.840	13	pyrolysis	[607]
*S-G* S-doped graphene	**SG**	200	0.860	13	annealing	[128]
*Se-CNTs-graphene-900* selenium-doped carbon nanotube/graphene networks	**CSe_nT_ 900/GSe**	141.54	0.883	13	annealing	[129]
*3D S-GNs* 3D sulfur-doped graphene networks	**SG_3Dχ_**	200	0.920	13	thermal annealing	[594]

^1^ For a proper comparison, this table contains only data from samples whose catalyst loading can be defined. ^2^ The catalyst name in italic is the catalyst name reported in the paper with the best E_onset_, reported in the third column and below the explanation reported in the paper; in bold is the name set using the terms reported in the legend, following mostly the indications reported in [892]. ^3^ Catalyst loading, when not reported in the paper, has been calculated from data provided by the authors. ^4^ E_onset_ was generally extracted from the LSV curves analyzed via the WebPlot program. ^5^ The synthesis method is not fully described but usually refers to the last treatment.

**Table 5:** Comparative analysis of some ORR parameters of the synthesized B-doped graphene ECs ^1^ with best performance in alkaline electrolytes.

Catalyst ^2^		Catalyst Loading ^3^ (μgcm^−2^)	E_onset_ vs. RHE ^4^ (V)	Electrolyte pH	Synthetic Method ^5^	Ref.
*BG* B-doped graphene	**GB**	39.81	0.790	13	thermal annealing	[636]
*PN-B2G* polynitrogen N8- (PN) deposited on boron-doped graphene	**N8-/GB (II)** **N8- = polynitrogen**	141.5	0.828	13	hydrothermal process	[653]
*B-G* B-doped graphene	**GB**	200	0.808	13	annealing	[128]
*B2-3DrGO* B-doped 3D-reduced graphene oxide	**B-rGO_3D_ (I)**	101.91	0.925	13	supercritical fluid process	[651]
*GH-BGQD2* B-doped graphene quantum dots anchored on a graphene hydrogel	**GB_qDot_/G_Hy_**	7961.78 7961.78	0.940 0.860	13 0.3	hydrothermal process	[638]

^1^ For a proper comparison, this table contains only data from samples whose catalyst loading can be defined. ^2^ The catalyst name in italic is the catalyst name reported in the paper with the best E_onset_, reported in the third column and below the explanation reported in the paper; in bold is the name set using the terms reported in the legend, following mostly the indications reported in [892]. ^3^ Catalyst loading, when not reported in the paper, has been calculated from data provided by the authors. ^4^ E_onset_ was generally extracted from the LSV curves analyzed via the WebPlot program. ^5^ The synthesis method is not fully described but usually refers to the last treatment.

**Table 6:** Comparative analysis of some ORR parameters of the synthesized P-doped graphene-based ECs ^1^ with best performance in alkaline electrolytes.

Catalyst ^2^		Catalyst Loading ^3^ (μgcm^−2^)	E_onset_ vs. RHE ^4^ (V)	Electrolyte pH	Synthetic Method ^5^	Ref.
*PG* P-doped graphene	**GP**	255	0.760	13	thermal annealing	[273]
*PG* P-doped graphene	**GP**	255	0.760	13	calcination	[869]
*P-G* P-doped Graphene	**GP**	200	0.772	13	annealing	[128]
*PG* Graphene and Carbon black composite	**C_b_/GP**	50.96	0.828	13	thermal annealing	[657]
*P-TRG* Metal-free phosphorus-doped graphene nanosheets	**GP_nSh_**	141.5	0.920	13	thermal annealing	[656]

^1^ For a proper comparison, this table contains only data from samples whose catalyst loading can be defined. ^2^ The catalyst name in italic is the catalyst name reported in the paper with the best E_onset_, reported in the third column and below the explanation reported in the paper; in bold is the name set using the terms reported in the legend, following mostly the indications reported in [892]. ^3^ Catalyst loading, when not reported in the paper, has been calculated from data provided by the authors. ^4^ E_onset_ was generally extracted from the LSV curves analyzed via the WebPlot program. ^5^ The synthesis method is not fully described but usually refers to the last treatment.

**Table 7:** Comparative analysis of some ORR parameters of the synthesized halogen-doped graphene-based ECs ^1^ with best performance in alkaline electrolytes.

Catalyst ^2^		Catalyst Loading ^3^ (μgcm^−2^)	E_onset_ vs. RHE ^4^ (V)	Electrolyte pH	Synthetic Method ^5^	Ref.
*ClFG* Fluoro and chloro-doped graphene	**FClG**	283.09	0.760	13	pyrolysis	[674]
*HIGnP* high-quality iodine-doped graphene nanoplatelets	**IG_nPl_**	101.91	0.770	13	annealing	[682]
*IGnP* edge-selectively-iodinated graphene nanoplatelets *BrGnP* edge-selectively-chlorinated graphene nanoplatelets *ClGnP* edge-selectively-iodinated graphene nanoplatelets	**IG_nPl_** **BrG_nPl_** **ClG_nPl_**	76.43 76.43 76.43	0.812 0.806 0.768	13 13 13	ball milling ball milling ball milling	[675] [675] [675]
*FIIRGO* Fluorinated reduced graphene oxide	**F-rGO (II)**	800	0.830	13	solution process	[672]
*I-graphene-900* Iodine-doped graphene	**IG 900**	28.31	0.855	13	annealing	[131]
*HI-RGO/CDs* Iodine-doped reduced graphene oxide/carbon dot composite	**IC_Dot_/I-rGO**	101.91	0.860	13	solution process	[684]
*I/rGO* Iodine-doped reduced graphene oxide	**I-rGO**	661.38	0.870	13	microwave heating	[688]
*FG−1100* Fluorine-doped graphene	**FG 1100**	100	0.940	13	thermal treatment	[673]

^1^ For a proper comparison, this table contains only data from samples whose catalyst loading can be defined. ^2^ The catalyst name in italic is the catalyst name reported in the paper with the best E_onset_, reported in the third column and below the explanation reported in the paper; in bold is the name set using the terms reported in the legend, following mostly the indications reported in [892]. ^3^ Catalyst loading, when not reported in the paper, has been calculated from data provided by the authors. ^4^ E_onset_ was generally extracted from the LSV curves analyzed via the WebPlot program. ^5^ The synthesis method is not fully described but usually refers to the last treatment.

**Table 8:** Comparative analysis of some ORR parameters of the synthesized multidoped graphene-based ECs ^1^ with best performance in alkaline electrolytes.

Catalyst ^2^		Catalyst Loading ^3^ (μgcm^−2^)	E_onset_ vs. RHE ^4^ (V)	Electrolyte pH	Synthetic Method ^5^	Ref.
*N–F-rGO* N and F-doped reduced graphene oxide	**F,N-rGO**	500	0.730	13	hydrothermal process	[692]
*NPHG 8* N,P-enriched hierarchically porous graphene	**NGP_hipo_**	127	0.735	13	carbonization	[766]
*SN-rGO 900* N and S-doped reduced graphene oxide	**N,S-rGO 900**	139,795	0.740	13	pyrolysis	[725]
*N/S co-doped graphene* Nitrogen and sulfur co-doped graphene	**NSG**	194.11	0.750	13	annealing	[709]
*B&N-rGO* N- and B-doped reduced graphene oxide	**N,B-rGO**	324.97	0.770	13	pyrolysis	[649]
*CNPS -900* N-, P-, S-tridoped graphene	**NSGP (III)**	668.79	0.780	13	annealing	[783]
*0.05-S, N-rGO* N,S co-doped graphene	**N,S-rGO (II)**	80	0.790 0.290	13 1 ^6^	hydrothermal process	[727]
*L-Cy-rGO* heteroatom (N, O, and S)-based L-cysteine-functionalized rGO	**LCy-rGO**	169.73	0.790	13	microwave treatment	[752]
*BNG* boron and nitrogen co-doped graphene	**NGB**	101.91	0.795	13	annealing	[744]
*N,S-CD/rGO* nitrogen and sulfur-doped carbon dot/graphene composite	**NSC_Dot_/rGO**	353.86	0.800	13	solvo-/hydrothermal process	[623]
*GC-NLS* graphene/C_nT_ composite doped sequentially with both nitrogen and sulfur	**NSG/CSN_nT_ (I)**	407.43	0.800	13	pyrolysis	[598]
*NSP-G^−^1636,94* *00* N,P,S-doped graphene	**NSGP 1000**	283.09	0.820	13	hydrothermal process	[715]
*S-N-rGO, 180* nitrogen and phosphorus co-doped reduced graphene oxide	**N,S-rGO (I)**	636.94	0.820	13	solution process	[730]
*N-P-rGO* nitrogen and phosphorus-co-doped graphene nanoribbons/CNTs composite	**N,P-rGO (I)**	191.39	0.830	13	solution process	[661]
*nGR-NF* graphene co-doped with nitrogen and fluorine	**FNG**	420	0.830	13	mechanochemical treatment	[777]
*B,N-graphene* B- and N-co-doped graphene	**NGB**	282.69	0.835	13	annealing	[639]
*NSG cv* S and N-doped graphene	**NSG (I)**	128	0.840	13	freeze drying	[618]
*NS-3DrGO 950* nitrogen and sulfur dual-doped three-dimensional reduced graphene oxide	**N,S-rGO_3D_ 950**	203.82	0.840	13	pyrolysis	[735]
*BNG 1000* boron and nitrogen-dual-self-doped graphene sheets	**NBG_Sh_ 1000**	607.15	0.840	13	pyrolysis	[750]
*NPG^−^1000* N,P-co-doped graphene	**NGP 1000**	80	0.845	13	pyrolysis	[760]
*N,S-GQDs/rGO* defect-rich nitrogen and sulfur-co-doped graphene quantum dots	**NSG_qDot_/rGO**	50.96	0.850	13	hydrothermal process	[619]
*BCN 4* B, N-co-doped graphene	**NGB (III)**	100	0.850	13	pyrolysis	[646]
*F,N,S-rGO* fluorine, nitrogen, and sulfur-doped graphene	**F,N,S-rGO** **F,N,S-rGO**	255 255	0.850 0.640	13 1 ^6^	pyrolysis	[616]
*carbonaceous rGO/E. coli* nitrogen and phosphorus-doped graphene–bacteria composite	**NCP/N,P-rGO**	509.55	0.85	13	annealing	[867]
*N-S/Gr1000* N- and S-doped graphene	**NSG 1000**	600 400 200 100	0.850 0.850 0.830 0.740	13	annealing	[720]
*SNGL-20* S, N co-doped, few-layered graphene oxides	**NSGO_fL_**	306	0.855	13	CVD	[593]
*PN-rGO* N and P-doped reduced graphene oxide	**N,P-rGO**	500	0.858	13	thermal annealing	[761]
*N–S-GAs 900* nitrogen and sulfur dual-doped three-dimensional (3D) graphene aerogels	**NSG_3DAe_ (I)**	425	0.859	13	pyrolysis	[714]
*NBGHS* boron and nitrogen co-doped hollow graphene microspheres	**NGB_hwmSp_**	130	0.860	13	calcination and etching	[643]
*NPS-G1* nitrogen, Sulfur, and Phosphorus co-doped graphene	**NSGP**	255	0.860	13	pyrolysis	[784]
*NS-G* nitrogen, sulfur-doped graphene nanoribbon	**GSN**	283	0.860	13	pyrolysis	[713]
*BNr-GO(S)* N- and B-doped reduced graphene oxide	**N,B-rGO_edr_**	408	0.860	13	freeze drying	[746]
*N,S-GNRs-2s* N and S-doped graphene nanoribbons	**NSG_nR_ (III)**	161.62	0.870	13	Solution process	[732]
*N, S-GCNT* N-doped graphene Sulfur, nitrogen co-doped nanocomposite of graphene and carbon nanotube	**NSC_nT_/NSG**	230	0.870	13	annealing	[608]
*HRBNG* highly reduced boron and nitrogen co-doped graphene	**N,B-rGO**	203.82	0.870	13	ball milling	[435]
*NPG* nitrogen and phosphorus-doped graphene	**NGP**	320	0.870	13	solvothermal process	[208]
*C/oMUS* nitrogen- and sulfur-co-doped carbon	**NSC_nT_/NSG**	400	0.870	13	pyrolysis	[702]
*BN-rGO* N and B-doped reduced graphene oxide	**N,B-rGO**	1019.11	0.870	13	annealing	[453]
*CDs/M-rGO* N,S, and P-doped carbon decorated reduced graphene oxide	**NSCP_Dot_/N,S,P-rGO**	101.92	0.879	13	hydrothermal process	[862]
*BN-GAs-2* boron and nitrogen co-doped graphene aerogels	**NGB_Ae_ (II)**	140	0.880	13	freeze drying	[751]
*NPCGF-7:3* carbon nanotubes (C) loaded on graphene microfolds	**N,P-C_nT_/NGP**	300	0.880	13	annealing	[764]
*inG* Iodine/nitrogen co-doped graphene	**NIG**	509.55	0.880	13	annealing	[678]
*N, P-GDs/N-3DG* nitrogen and phosphorus co-doped graphene dots supported on nitrogen-doped three-dimensional graphene	**NGP_Dot_/GN_3D_**	800	0.880	13	annealing	[663]
*N,P-CGHNs* nitrogen and phosphorous-doped CNTs and graphene hybrid nanospheres	**(NPC_nT_/NGP)_nSp_** **(NPC_nT_/NGP)_nSp_**	300 600	0.890 0.830	13 1 ^6^	annealing	[758]
*GSP* pyrolyzed PG with SO3*Py	**NSGpo (II)**	300	0.890	13	pyrolysis	[721]
*Gl300G-900* N,S,B, P-doped graphene	**NSGPB (I)**	300	0.890	13	pyrolysis	[786]
*NFG* nitrogen-doped fluorinated graphene	**FNG**	283	0.898	13	annealing	[669]
*BNPGA−1000^−1^5* boron, nitrogen, phosphorus ternary doped graphene aerogels	**NGPB_Ae_ 1000 (I)**	600	0.900	13	thermal treatment	[691]
*N,B-GA-900* nitrogen and boron co-doped graphene aerogel	**NGB_Ae_ 900**	212	0.909	13	pyrolysis	[645]
*NSGCB* carbon black incorporated nitrogen and sulfur co-doped graphene	**NSG/NSC_b_**	306.12	0.910	13	pyrolysis	[708]
*PNGF_ADP(op)* N and P co-doped graphene framework	**NGPχ (IV)**	100.57	0.910	13	calcination	[768]
*BN-Gas* N and B-three-dimensional graphene aerogels	**NGB_3DAe_**	160	0.910	13	thermal reduction	[748]
*N-F/rGO* nitrogen and fluorine-doped reduced graphene oxide	**F,N-rGO**	500	0.910	13	thermal annealing	[776]
*N/S-G-7d* nitrogen/sulfur doped graphene	**NSG (III)**	242.64	0.911	13	annealing	[719]
*NH_2_-G-F5* amino (NH2)-/fluoro (F)-cofunctionalized graphene	**NH_2_-FG (II)**	236	0.915	13	solution process	[774]
*NF–MG3* nitrogen and fluorine-dual-doped mesoporous graphene	**FNG_mpo_ (III)**	400	0.920	13	pyrolysis	[670]
*NB-3DGN* N and B-doped Three-dimensional graphene networks	**NGB_3Dχ_**	16	0.928	13	electrochemical doping	[655]
*tdgc-900* *three-dimensional* (3D DOPED graphene-based carbon (interconnected porous foam)	**NCP 900/NGP_3DpoFo_**	153	0.930	13	pyrolysis	[726]
*CNx/CSx-GNRs 1000 2.5h* nitrogen/sulfur co-doped graphene nanoribbons	**NSG_henR_ 1000** **NSG_henR_ 1000**	81.53 81.53	0.930 0.500	13 0.3 ^6^	thermal annealing	[604]
*NSG@CNT-2* N/S co-doped CNT-graphene hybrids	**NSG/C_nT_ (II)**	180	0.930	13	pyrolysis	[701]
*NSG* nitrogen and sulfur dual-doped graphene	**NSG**	230	0.930	13	pyrolysis	[609]
*NP + NPY/PG* nitrogen and phosphorus co-doped graphene	**NGP**	600	0.930	13	pyrolysis	[769]
*NS/G-AA* N,S co-doped graphene nanosheets	**NG_nSh_ (II)**	600	0.930	13	pyrolysis	[734]
*BN-GQD/G-30* boron- and nitrogen-doped graphene quantum	**NGB_qDot_/G (II)**	81.53	0.935	13	CVD	[747]
*NSG* three-dimensional hierarchical porous nitrogen and sulfur co-dopedgraphene nanosheets	**NSG_3DhiponSh_** **NSG_3DhiponSh_**	102 102	0.935 0.685	13 0.3 ^6^	pyrolysis	[606]
*N-S-G 1000* nitrogen and sulfur co-doped graphene	**NSG 1000**	566	0.939	13	solution polymerization	[705]
*N,S-PGN 700 rGO* nitrogen/sulfur co-doped porous graphene networks	**NSG_poχ_ 700 (III)**	300	0.940	13	annealing	[729]
*NS graphene (1:1)* N and S-doped graphene	**NSG (IV)**	600	0.940	13	pyrolysis	[733]
*BNG etched* boron, nitrogen-doped graphene	**NGB (I)**	80.95	0.945	13	annealing	[745]
*N,P-GCNS* nitrogen and phosphorus dual-doped graphene/carbon nanosheets	**NCP/NGP_nSh_**	141	0.950	13	electrochemical preparation	[759]
*P–N-GFs-HMPA* high performancemetal-free P,N-doped carbon catalysts	**NCP_nT_/NGP_Fo_ (I)** **NCP_nT_/NGP_Fo_ (I)**	360 600	0.950 0.880	13 1 ^6^	solution process	[660]
*FN3SG* N,F, and S tri-doped porous graphene	**FNSG_po_** **FNSG_po_**	200 200	0.960 0.710	13 0.3 ^6^	pyrolysis	[778]
*B,N-PG-O^−1^5* boron and nitrogen-doped porous graphene	**N,B-rGO_po_ (I)**	255 255	0.960 0.760	13 0.3 ^6^	annealing	[742]
*GNF-H/N-F* heteroatom (N,F)-doped various graphitic carbon nanofibers	**FNC_Fb_/FNG_nSh_(III)**	500	0.980	13	pyrolysis	[779]

^1^ For a proper comparison, this table contains only data from samples whose catalyst loading can be defined. ^2^ The catalyst name in italic is the catalyst name reported in the paper with the best E_onset_, reported in the third column and below the explanation reported in the paper; in bold is the name set using the terms reported in the legend, following mostly the indications reported in [892]. ^3^ Catalyst loading, when not reported in the paper, has been calculated from data provided by the authors. ^4^ E_onset_ was generally extracted from the LSV curves analyzed via the WebPlot program. ^5^ The synthesis method is not fully described but usually refers to the last treatment. ^6^ For a direct comparison, values at different pH have also been reported.

**Table 9:** Comparative analysis of some ORR parameters of the synthesized graphene, composites, and variously doped graphene-based ECs ^1^ with best performance in acidic electrolytes.

Catalyst ^2^		Catalyst Loading ^3^ (μgcm^−2^)	E_onset_ vs. RHE ^4^ (V)	Electrolyte pH	Synthetic Method ^5^	Ref.
*EG-AQ 1* electrochemical grafted anthraquinone functionalized graphene sheets	**AQ-G_Sh_ (I)** **AQ = anthraquinone C14H8O2**	102.04 102.04	0.140 0.729	1 13 ^6^	electrochemical grafting and functionalization	[793]
*GNR1* graphene nanoribbons	**G_nR_ (I)** **G_nR_ (I)** **G_nR_ (I)**	80 80 80	0.170 0.450 0.760	1 7 ^6^ 13 ^6^	solution process	[447]
*S-rGO* S-doped reduced graphene oxide	**S-rGO** **S-rGO**	255 255	0.170 0.740	1 13 ^5^	solution process	[615]
*GP-BM* graphene prepared via ball milling	**G**	310.70	0.269	1	ball milling	[795]
*0.05-S, N-rGO* N,S co-doped graphene	**N,S-rGO (II)** **N,S-rGO (II)**	80	0.290 0.790	1 13 ^6^	hydrothermal process	[727]
*S-rGO* S-doped reduced graphene oxide	**S-rGO**	255	0.300	1 ^6^	reflux solution	[615]
*P(1,5-DAAQ)/RGO* Poly(1,5- diaminoanthraquinone)/reduced graphene oxide nanohybrid	**P(1,5-DAAQ)/rGO** **P(1,5-DAAQ) =** **Poly(1,5-** **Diaminoanthraquinone) = (C4H7O2N2)n**	200	0.327	0.3	potentiodynamic deposition	[792]
*CNx/CSx-GNRs 1000 2.5h* nitrogen/sulfur co-doped graphene nanoribbons	**NSG_henR_ 1000** **NSG_henR_ 1000**	81.53 81.53	0.500 0.930	0.3 13 ^6^	thermal annealing	[604]
*rGO/PANI/PVA*	**PANI/PVA/rGO** **PVA = polyvinyl alcohol (C2H4O)n** **PANI =** **POYLANILINE [C6H4NH]n**	9	0.610	0.3	chemical reduction in solution	[881]
*F,N,S-rGO* fluorine nitrogen and sulfurl-doped graphene	**F,N,S-rGO** **F,N,S-rGO**	255 255	0.640 0.850	1 13 ^6^	pyrolysis	[616]
*NSG* three-dimensional hierarchical porous nitrogen and sulfur-codoped graphene nanosheets	**NSG_3DhiponSh_ NSG_3DhiponSh_**	102 102	0.685 0.935	0.3 13 ^6^	pyrolysis	[606]
*FN3SG* N, F, S tri-doped porous graphene	**FNSG_po_** **FNSG_po_**	200 200	0.710 0.960	0.3 13 ^6^	Reflux solution	[778]
*NPC/G* N, P dual-doped carbon/graphene	**G/NCP (I)**	255	0.730	1	calcination	[869]
*B,N-PG-O-15* Boron and nitrogen-doped porous graphene	**N,B-rGO_po_ (I)** **N,B-rGO_po_ (I)**	255 255	0.760 0.960	0.3 13 ^6^	annealing	[742]
*N-HC@G-900* ultrathin N-doped holey carbon/graphene hybrid	**CN_hl_ 900/G** **CN_hl_ 900/G**	250 250	0.770 0.940	0.3 13 ^6^	calcination	[842]
*PNGr* phosphorous and nitrogen-doped graphene	**NGP**	707.71	0.770	1	pyrolysis	[640]
*NSG@CNT-3* hybrid N,S-doped CNT-graphene nanocomposites	**NSG/C_nT_ (III)**	300	0.820	1	pyrolysis	[701]
*N,P-CGHNs* nitrogen and phosphorous doped CNTs and graphene hybrid nanospheres	**(NCP_nT_/NGP)_nSp_** **(NCP_nT_/NGP)_nSp_**	600 300	0.830 0.890	1 13 ^6^	annealing	[758]
*GH-BGQD2* B-doped graphene quantum dots anchored on a graphene hydrogel	**GB_qDot_/G_Hy_ (II)** **GB_qDot_/G_Hy_ (II)**	7961.78 7961.78	0.860 0.940	0.3 13 ^6^	pyrolysis	[638]
*P–N-GFs-HMPA* high performancemetal-free P,N-doped carbon catalysts	**NCP_nT_/NGP_Fo_ (I)** **NCP_nT_/NGP_Fo_ (I)**	600 0.360	0.880 0.950	1 13 ^6^	solution process	[660]

^1^ For a proper comparison, this table contains only data from samples whose catalyst loading can be defined. ^2^ The catalyst name in italic is the catalyst name reported in the paper with the best E_onset_, reported in the third column and below the explanation reported in the paper; in bold is the name set using the terms reported in the legend, following mostly the indications reported in [892]. ^3^ Catalyst loading, when not reported in the paper, has been calculated from data provided by the authors. ^4^ E_onset_ was generally extracted from the LSV curves analyzed via the WebPlot program. ^5^ The synthesis method is not fully described but usually refers to the last treatment. ^6^ For a direct comparison, values at different pH have also been reported.

**Table 10:** Comparative analysis of some ORR parameters of the synthesized graphene, composites, and variously heteroatom-doped graphene-based ECs ^1^ with best performance in neutral electrolytes.

Catalyst ^2^		Catalyst Loading ^3^ (μgcm^−2^)	E_onset_ vs. RHE ^4^ (V)	Electrolyte pH	Synthetic Method ^5^	Ref.
*AQS/RGO* anthraquinone monosulfonate sodium/reduced graphene oxide nanocomposite	**AQS/rGO** **AQS = C_14_H_7_NaO_5_S**	5.67	0.440	7	solution process	[889]
*N/S-G* nitrogen and sulfur dual-doped graphene	**NSG**	141.54	0.441	7	pyrolysis	[731]
*RGSs* reduced graphene oxide sheets amino-functionalized graphene	**rGO_Sh_**	200	0.460	7	electrochemical reduction	[790]
*DNGS 480 900* dendritic N-doped graphene spheres	**NG_ddSp_ 900** **NG_ddSp_ 900** **NG_ddSp_ 900**	1000 1000 1000	0.470 0.550 0.770	7 0.3 ^6^ 13 ^6^	pyrolysis	[532]
*CN 1000* *N-doped graphene*	**NG 1000** **NG 1000**	144 144	0.567 0.847	7,4 14^6^	thermal treatment	[390]
*GNR1* graphene nanoribbons	**G_nR_** **G_nR_** **G_nR_**	80 80 80	0.450 0.760 0.170	7 13 ^6^ 1 ^6^	solution process	[447]
*NG/CB 10* porous N-doped graphene/carbon black composite	**NG/C_b_ (IV)**	100	0.780	7,4	pyrolysis	[344]
*5rG@NHCS* reduced GO-modified ultra-thin nitrogen-doped hollow carbon sphere composites	**rGO/CN_hwnSp_ (II)** **rGO/CN_hwnSp_ (II)**	1400 1400	0.851 0.882	7 13 ^6^	pyrolysis	[845]
*I-NG* N-doped graphene with implantation of nitrogen active sites via C3N4	**C_3_N_4_/NG**	283.09	0.990	7	sonochemical process	[312]

^1^ For a proper comparison, this table contains only data from samples whose catalyst loading can be defined. ^2^ The catalyst name in italic is the catalyst name reported in the paper with the best E_onset_, reported in the third column and below the explanation reported in the paper; in bold is the name set using the terms reported in the legend, following mostly the indications reported in [892]. ^3^ Catalyst loading, when not reported in the paper, has been calculated from data provided by the authors. ^4^ E_onset_ was generally extracted from the LSV curves analyzed via the WebPlot program. ^5^ The synthesis method is not fully described but usually refers to the last treatment. ^6^ For a direct comparison, values at different pH have also been reported.

**Table 11:** Comparative analysis of some ORR parameters of the synthesized graphene-based ECs ^1^ with best performance in alkaline electrolytes.

Catalyst ^2^		Catalyst Loading ^3^ (μgcm^−2^)	E_onset_ vs. RHE ^4^ (V)	Electrolyte pH	Synthetic Method ^5^	Ref.
*LIG-O* oxidized laser-induced graphene	**G (I)**	150.98	0.690	13	plasma treatment	[802]
*Gdots* graphene quantum dts	**G_qDot_**	100.90	0.539	13	electrochemical process	[775]
*GO 1000* edge-rich non-doped graphene	**G_edr_ 800**	40	0.640	13	pyrolysis	[801]
*H-rGO-sp* heat-treated reduced graphene oxide-based hollow spheres	**rGO_hwSp_**	141.54	0.710	13	solution process	[444]
*EG-AQ 2* electrochemical-grafted anthraquinone functionalized graphene sheets	**AQ-G_Sh_ (II)** **AQ = anthraquinone**	102.04 102.04	0.729 0.140	13 1 ^6^	electrochemical grafting and functionalization	[793]
*Graphene*	**G**	90.8 453.9	0.740 0.770	13 13	thermal treatment	[814]
*G* graphene	**G**	121.46	0.740	13	pyrolysis	[816]
*graphene–C60 hybrid*	**C_60_-G**	50	0.750	13	freeze drying	[794]
*10K* graphene nanosheets	**G_nSh_ (III)**	283	0.758	13	solution process	[506]
*rGO pH 10* reduced graphene oxide nanoscrolls	**rGO_nSc_**	60	0.760	13	freeze casting process	[530]
*GNR1* graphene nanoribbon	**G_nR_ (I)** **G_nR_ (I)** **G_nR_ (I)**	80 80 80	0.760 0.170 0.450	13 1 ^6^ 7 ^6^	solution process	[447]
*GC/Graphene1*	**G(I)**	100	0.780	13	solution process	[813]
*DMG−10* doping-free, multi-defect graphene	**G_df_ (I)**	253.44	0.810	13	hydrothermal process	[805]
*DTG* 3D double-layer templated graphene	**G_3D_**	255	0.820	13	CVD	[798]
*MC-G700* mechanochemically synthesized multilayer graphene	**G_mL_ (II)**	1530	0.820	14	thermal treatment	[806]
*H-rGO* hydrazine-reduced graphene oxide	**rGO**	151.65	0.840	13	solution process	[500]
*P-G* plasma-treated graphene	**G(I)**	151.65	0.840	13	Plasma treatment	[797]
*GP-BM* graphene prepared via ball milling	**G**	310.70	0.867	14	ball milling	[795]
*8Co/G 700-HCl* holey graphene	**G 700 (V)**	152.8	0.870	13	chemical etching	[799]
*F-graphene* graphene fragments	**G_Fg_ (I)**	283.09	0.880	13	grinding	[800]
*3D-PGM^−1^150* 3D interconnected hierarchical porous graphene mesh	**G_3Dhipomh_ 1150**	100	0.930	13	self-assembly and chemical etching	[807]

^1^ For a proper comparison, this table contains only data from samples whose catalyst loading can be defined. ^2^ The catalyst name in italic is the catalyst name reported in the paper with the best E_onset_, reported in the third column and below the explanation reported in the paper; in bold is the name set using the terms reported in the legend, following mostly the indications reported in [892]. ^3^ Catalyst loading, when not reported in the paper, has been calculated from data provided by the authors. ^4^ E_onset_ was generally extracted from the LSV curves analyzed via the WebPlot program. ^5^ The synthesis method is not fully described but usually refers to the last treatment. ^6^ For a direct comparison, values at different pH have also been reported.

**Table 12:** Comparative analysis of some ORR parameters of the synthesized graphene composite-based ECs ^1^ with best performance in alkaline electrolytes.

Catalyst ^2^		Catalyst Loading ^3^ (μgcm^−2^)	E_onset_ vs. RHE ^4^ (V)	Electrolyte pH ^5^	Synthetic Method ^6^	Ref.
*C50* vertical graphene sheets on separated papillary granules formed nanocrystalline diamond films	**G_v_/C_DdFm_ (III)**	80	0.670	13	plasma etching	[827]
*Si–GQD1.0 NCs* hybrid silicon nanosheets (NSs)–graphene quantum dot nanocomposites	**Si_nSh_/G_qDot_**	1040	0.670	13	solution process	[825]
*GOBN5 700* reduced graphene oxide/boron nitride composite annealed at 700	**BN_hg_/rGO**	42.26	0.680	13	annealing	[888]
*NCGO0.05 1000* nitrogen-doped carbon graphene composite	**CNpo_shell/_rGO_core_ 1000**	127	0.700	13	thermal treatment	[831]
*C3N4@rGO* graphitic carbon nitride on reduced graphene oxide	**C3N4/rGO**	849	0.720	13	pyrolysis	[859]
*N-DC/G* three-dimensional honeycomb-like nitrogen-doped carbon nanosheet/graphene	**NC_3DhcnSh_/G**	99.8	0.735	13	carbonization	[839]
*CNx/G-600* carbon-nitrogen/graphene composite	**NC 600/rGO**	509.55	0.760	13	pyrolysis	[832]
*GH-GQD 90* graphene quantum dots/graphene hydrogel composites	**G_qDot_/G_Hy_ (II)**	100	0.780	13	hydrothermal process	[804]
*NCNTs/G* three-dimensional (3D) nitrogen-doped carbon nanotubes/graphene	**NC_nT_/G_3D_**	1000	0.780	13	pyrolysis	[866]
*MCN−1_G3* triazole-based mesoporous C3N5 a graphene hybrid	**C_3_N_5mpo_/G (I)**	13.876	0.780	13	calcination and etching	[849]
*PEDOT/rGO/H_2_SO_4_* PEDOT:PSS/Graphene Composite	**PEDOT + PSS/rGO (I)** **PEDOT = Poly(3,4-ethylenedioxythiophene)** **PSS = Polystyrene**	120	0.785	13	heating in solution	[878]
						
*g-C3N4/rGO* ultrathin g-C3N4 nanosheets and reduced graphene oxide hybrid	**g-C3N4_uth_/rGO (I)**	300	0.795	13	photoreduction	[855]
*G-PMF1−1* graphene-based composite sheets	**NC/G/NC (II)**	80	0.799	13	annealing	[833]
*N-CDs/G* nitrogen-doped carbon dots decorated on the graphene surface	**NC_tDot_/G**	50.95	0.820	13	hydrothermal process	[860]
*hp-GSGCN_2x* hierarchically porous graphene sheets/graphitic carbon nitride intercalated composites	**G_Sh_/g-C_3_N_4_ (I)**	283.08	0.825	13	thermal annealing	[854]
*HMCG Cys* heteroatom (N and S) doped mesoporous carbon/graphene	**NSC_mpo_/G (I)**	200	0.838	13	nanocasting approach	[865]
*0.47%* boron-doped carbon catalysts on graphene	**BC/G (I)**	223	0.840	13	liquid-phase electrodeposition	[830]
*A/graphene* adenine functionalized graphene	**A/G** **A = adenine**	250	0.840	13	ultrasound	[877]
*N-VA-CNTs/GF* free-standing vertically aligned nitrogen-doped carbon nanotube supported by graphene foam	**NC_valnT_/G_Fo_**	143.31	0.850	13	PECVD	[884]
*P-T/rGO* covalently linked pyridine and thiophene molecule (P-T) polymer composite with reduced graphene oxide	**(C45H74N4B2F2S)n/rGO**	521.13	0.850	13	sonication	[879]
*GMC-s* graphene-based microporous carbons	**SC/G/SC_L_**	600	0.850	13	pyrolysis	[834]
*FPCFs@rGO* fullerene-derived porous carbon fibers (FPCFs)/reduced graphene oxide composite	**C60_poFb_/rGO (II)**	250	0.870	13	calcination	[828]
*NS-CD@gf_a900* carbon dot-embedded porous graphene	**NSC_Dot_ 900/rGO**	280	0.870	13	annealing	[863]
*G-CN 800* graphene-based carbon nitride nanosheets	**CN_x_ 800/G**	70,7	0.878	13	pyrolysis	[852]
*5rG@NHCS* reduced GO (rG) modified ultra-thin nitrogen-doped hollow carbon sphere (NHCS) composites	**rGO/NC_uthwnSp_ (II)** **rGO/NC_uthwnSp_ (II)**	1400 1400	0.882 0.851	13 7^6^	pyrolysis	[845]
*N-APC-Gr-900* N-doped hybrid carbons	**NC 900/G**	400	0.890	13	carbonization	[848]
*mNC/rGO* mesoporous nitrogen-doped carbon/reduced graphene oxide	**NC_mpo_/rGO**	240	0.900	13	annealing	[843]
*NPC/G* N, P dual-doped carbon/graphene	**G/NCP (I)**	255	0.900	13	calcination	[869]
*rGO@PN\C-2* phosphorus- and nitrogen-doped carbon nanosheets	**(NCP/rGO/NPC)_nSh_ (II)**	200	0.910	13	carbonization	[870]
MTCG monoatomic-thick g-C3N4dots@ graphene	**C_3_N_4mtDot_/G**	80	0.915	13	hydrothermal process	[856]
*N-PC@G-0.02* N-doped porous carbon@graphene composites	**NC_po_/G/NC_po_ (I)**	407.64	0.920	13	pyrolysis	[838]
*N-MCS@rGO* N-doped mesoporous carbon spheres loaded on reduced graphene oxide nanosheets	**NC_mpoSp_/rGO_nSh_**	720	0.920	13	carbonization	[847]
*rGO/PANI/PVA* reduced GO polyvinyl alcohol polyaniline nanocomposites	**PANI/PVA/rGO** **PVA = polyvinyl alcohol** **PANI =** **polyaniline**	9	0.930	13.7	chemical reduction in solution	[881]
*PANRGO700* porous carbon nanoballs on graphene composite	**NC_nBl_ 700/rGO**	200	0.930	13	pyrolysis	[846]
*N-HC@G-900* ultrathin N-doped holey carbon/graphene hybrid	**CN_hl_900/G** **CN_hl_900/G**	250 250	0.940 0.770	13 0.3 ^6^	calcination	[842]
*N-MC/rGO-800* graphene-supported nitrogen-doped mesoporous carbons	**NC_mpo_/rGO/NC_mpo_ 900**	204	0.950	13	pyrolysis	[840]
*NPCS-850* 2D nitrogen-doped porous carbon sheet	**NC_2DpoSh_ 850/G**	250	0.970	13	pyrolysis	[841]
*G-GCN* graphitic carbon nitride immobilized on graphene	**g-C3N4/G**	714.29	0.979	13	solution chemistry	[851]

^1^ For a proper comparison, this table contains only data for samples whose catalyst loading can be defined. ^2^ The catalyst name in italic is the catalyst name reported in the paper with the best E_onset_, reported in the third column and below the explanation reported in the paper; in bold is the name set using the terms reported in the legend, following mostly the indications reported in [892]. ^3^ Catalyst loading, when not reported in the paper, has been calculated from data provided by the authors. ^4^ E_onset_ was generally extracted from the LSV curves analyzed via the WebPlot program. ^5^ The synthesis method is not fully described but it usually refers to the last treatment. ^6^ For a direct comparison, values at different pH have also been reported.

## Supplementary Materials

The following supporting information can be downloaded at https://www.mdpi.com/article/10.3390/molecules30102248/s1.
