# Supplementary material for: Metal-Free Graphene-Based Derivatives as Oxygen Reduction Reaction Electrocatalysts in Energy Conversion and Storage Systems: An Overview
Source: Molecules. 2025 May 21;30(10):2248. doi: 10.3390/molecules30102248 (PMC12542381; doi:10.3390/molecules30102248)
Supplement: Supplementary file 1 [file molecules-30-02248-s001.zip › molecules-3545008-supplementary.pdf]

Legenda

Activated= ac

Defective= df

Edge-rich=edr

Ultrathin= ut

Helical=he

Holey=hl

Hollow=hw

Few-layered= fl

Hierarchical=hi

Dendritic=dd

Black= b

Ultrathin= uth

Sub-micron=sμ

Monoatomic=mt

honeycomb-like=Hc

Vertically aligned=val

Hexagonal= hg

Nanoporous= npo

Macroporous= mapo

Mesoporous=mpo

Microporous= μpo

One or two or three-dimensional = 1D, 2D, 3D

Nanoscrolls=nSc

Fragment=Fg

Film= Fm

Aerogel= Ae

Hydrogel=Hy

NanoCapsule=nCs

Nanoribbons nR,

Nanoballs= nBl

Fiber=Fb

Few Layers=fL

Multi Layers=mL

Nanotube= nT

Diamond= Dd

Single Walled Carbon Nanotube= swCnT

Multi Walled Carbon Nanotube= mwCnT

Tetrapod= tPod

Multilayer= mL

Cage=Cg

Nanoclusters=nCl

Nanosphere=nSp

Nanosheet=nSh

Cloath=Cth

Dot=Dot

Quantum Dot= QDot

Framework=χ
